# A putatively extinct higher taxon of Spirotrichea (Ciliophora) from the Lower Cretaceous of Brazil

**DOI:** 10.1038/s41598-021-97709-2

**Published:** 2021-09-27

**Authors:** Thiago da Silva Paiva, Ismar de Souza Carvalho

**Affiliations:** 1 Departamento de Zoologia, Universidade Federal do Rio de Janeiro, Instituto de Biologia, Avenida Carlos Chagas Filho, 373, Bloco A, Sala 074, Ilha do Fundão, Cidade Universitária, Rio de Janeiro, RJ 21941-902 Brazil; 2 Departamento de Geologia, Universidade Federal do Rio de Janeiro, Instituto de Geociências, Avenida Athos da Silveira Ramos, 274, Bloco F, Ilha do Fundão Cidade Universitária, Rio de Janeiro, RJ 21949-900 Brazil

**Keywords:** Palaeontology, Microbiology

## Abstract

Fossil microeukaryotes are key elements for understanding ancient ecosystems at microscopic level and improving the knowledge on the diversification of microbial life as a whole. We describe *Palaeohypothrix bahiensis* gen. *et* sp. nov., an exceptionally well-preserved Lower Cretaceous (Berriasian–Barremian; 145–125 Mya) amber-entrapped microeukaryote, identified as a spirotrich ciliate. The preservation of structures interpreted as the nuclear apparatus and remains of the ciliature revealed a novel ground plan, not found in modern Spirotrichea, thus representing a putatively extinct higher taxon lineage, viz. the Palaeohypotricha nov. tax. Based on cladistic analysis, the new taxon is hypothesized as phylogenetically related to the Protohypotrichia.

## Introduction

The Spirotrichea is a broadly diversified class of intramacronucleate ciliates characterized by having a polyhymenophore adoral zone, complex specialization of the ventral ciliature forming compound cilia (= cirri) in most taxa, and macronuclear DNA replication bands during cell division^1^. The group encompasses the Discocephalida, Euplota, Hypotricha and Protohypotrichia, often referred to as hypotrichs *s. l.*^2^, plus the Licnophoria, Oligotricha, and the monotypic Phacodiniidia, all frequently regarded as higher taxa, but at variable ranks^1,3–5^. Albeit common in freshwater, marine and terrestrial environments worldwide, very little is known on the natural history of the Spirotrichea spanning the fathom of geological time. Recent molecular analysis estimates their origin in the Neoproterozoic, at approximately 850 Mya^6^, however, known fossil record is mostly restricted to tintinnid oligotrichs, of which unambiguous registry of preserved loricae first appeared in the Jurassic and continue to the Recent^7^. As the other taxa are formed by soft-bodied unicellular organisms, preservation is largely impaired. To our knowledge, fossils were only reported for euplotids, namely *Aspidisca eocenica*, *Cinetoconia crassa* and *Ploesconia cycloides* (all of which identity have been doubted^8^) found in Eocene lignite^9,10^, and one undetermined hypotrich in Cenomanian amber from France^11^.

### Systematic paleontology

Alveolata Cavalier-Smith, 1991

Ciliophora Doflein, 1901

Intramacronucleata Lynn, 1996

Spirotrichea Bütschli, 1889

Palaeohypotricha nov. tax. †

*Palaeohypothrix bahiensis* gen. *et* sp. nov.

#### Etymology

Greek: *παλαιός* (ancient, old) + *hupó* (under) + θρίξ (hair). Named for being an ancient hypotrich *s. l.* ciliate. The species-group name *bahiensis* refers to the State of Bahia, Brazil, from where the amber was collected.

#### Holotype

Universidade Federal do Rio de Janeiro, Departamento de Geologia, Coleção de Macrofósseis. Accession data: Holotype—UFRJ-DG 762 Pb. No further specimens found.

#### Stratigraphic horizon and location

Amber from lacustrine Lower Cretaceous (Berriasian–Barremian; 145 to 125 Mya) sandstones, Caruaçu Member, Maracangalha Formation, Recôncavo Basin, Ilha dos Frades, Bahia state, Brazil. Geographic coordinates: 12° 48′ 20.3″ S 38° 37′ 45.0″ W.

#### Diagnosis

**Figure 1:**
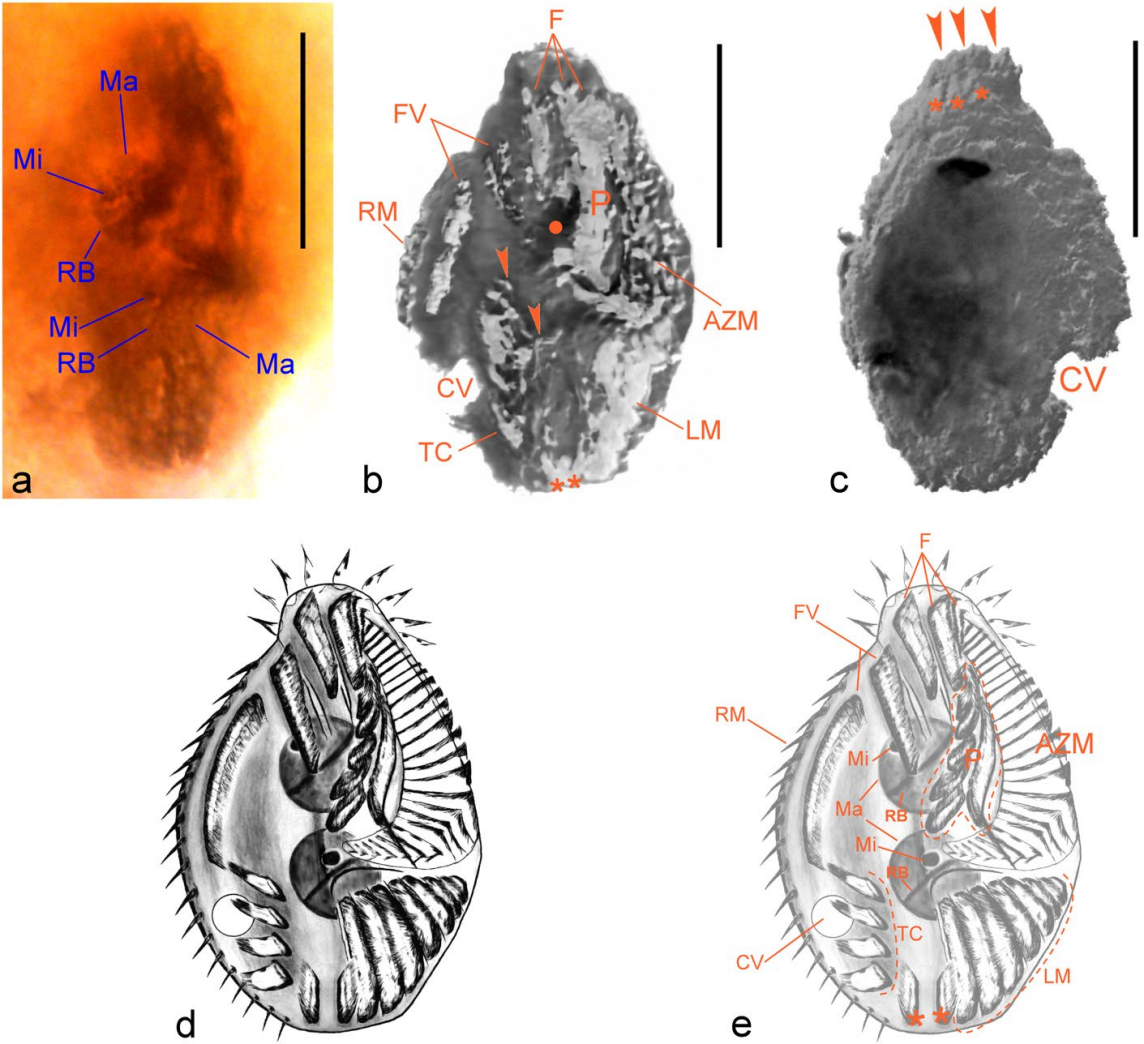
Morphology of *Palaeohypothrix bahiensis* gen. *et* sp. nov. (**a**) Photograph of holotype (ufrjdg762pb0d0019.png in Supplementary Data 1) showing remains of the nuclear apparatus. (**b**) Composition assembled from parts of volumetric reconstruction sections where the external amber layer could be virtually removed from the ventral side, showing presumed remains of ciliature elements and contractile vacuole. The dot shows darkened area where part of the anterior macronuclear nodule is seen by transparency; asterisks mark short ciliary brushes right of left marginal ciliature and arrowheads show spots with undetermined surface structures. **c**. Volumetric reconstruction of dorsal side with external amber layer removed, showing dorsal ribs (asterisks) and attachment sites of crown membranelles (arrowheads). (**d**,**e**) Interpretative reconstruction of live specimen depicting the ventral side; structures marked in (**e**). Legends: *AZM* adoral zone of membranelles, *CV* contractile vacuole, *F* frontal ciliary structures, *FV* fronto-ventral ciliary structures, *LM* left marginal ciliature, *Ma* macronuclear nodule, *Mi* micronucleus, *P* paroral membrane, *RB* vestige of macronuclear DNA replication band, *RM* right marginal ciliature, *TC* transverse cirri-like structures. Scale bars = 20 µm.

**Figure 2:**
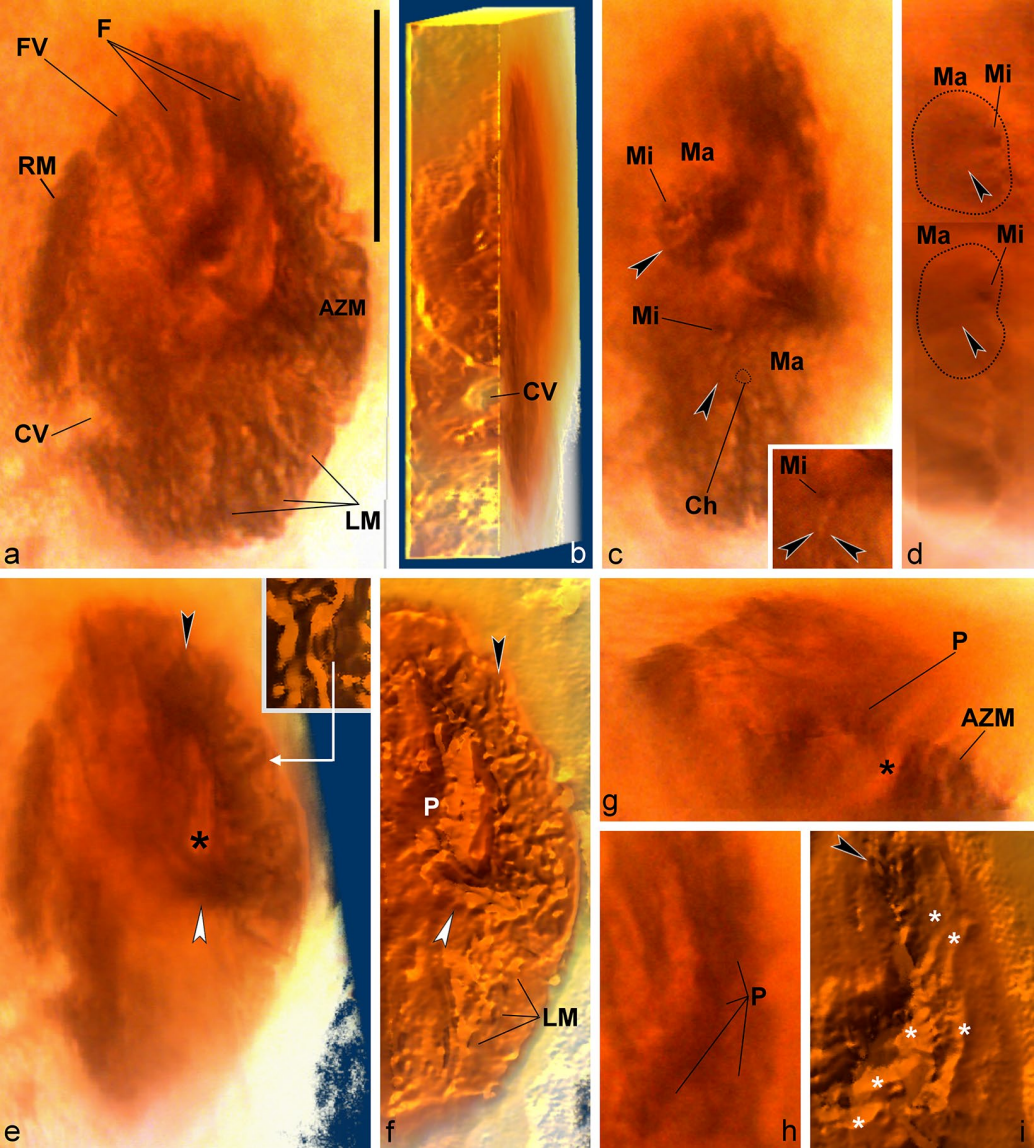
Light microscopy photographs (**a**,**c**,**h**) and volumetric reconstructions (**b**,**d**,**e–g**,**i**) of *Palaeohypothrix bahiensis* gen. *et* sp. nov. holotype. (**a**) Z-stack of photographs to compose the entire ventral surface. Ventral side. (**b**) Right side to show presumed contractile vacuole. (**c**,**d**) Detail of nuclear apparatus; bottom right inset in c. to show slightly different focal plane. (**c**) Ventral view. (**d**) Right lateral view assembled from two longitudinal sections to show both macronuclear nodules, circumscribed by dotted line. (**e**) Ventrolateral view to show the adoral zone of membranelles; figure inset shows detail of one lapel membranelle. Asterisk marks oral cavity; black and white arrowheads point to distal and proximal ends of lapel adoral zone, respectively (same in **f**). (**f**) A ventral focal plane under simulated oblique illumination. (**g**) Transversal section showing oral cavity (asterisk). (**h**,**i**) Detail of paroral. (**h**) Bright field photograph. (**i**) Simulated oblique illumination. Arrowhead shows insertion pits of frontal brushes; asterisks mark ciliary brushes which compose the paroral. Legends: *AZM* adoral zone of membranelles, *Ch* vestige of chromatin condensation, *CV* contractile vacuole, *F* frontal ciliary structures, *FV* fronto-ventral ciliary structures, *LM* left marginal ciliature, *Ma* macronuclear nodule, *Mi* micronucleus, *P* paroral membrane, *RM* right marginal ciliature. Scale bar = 20 µm.

**Figure 3:**
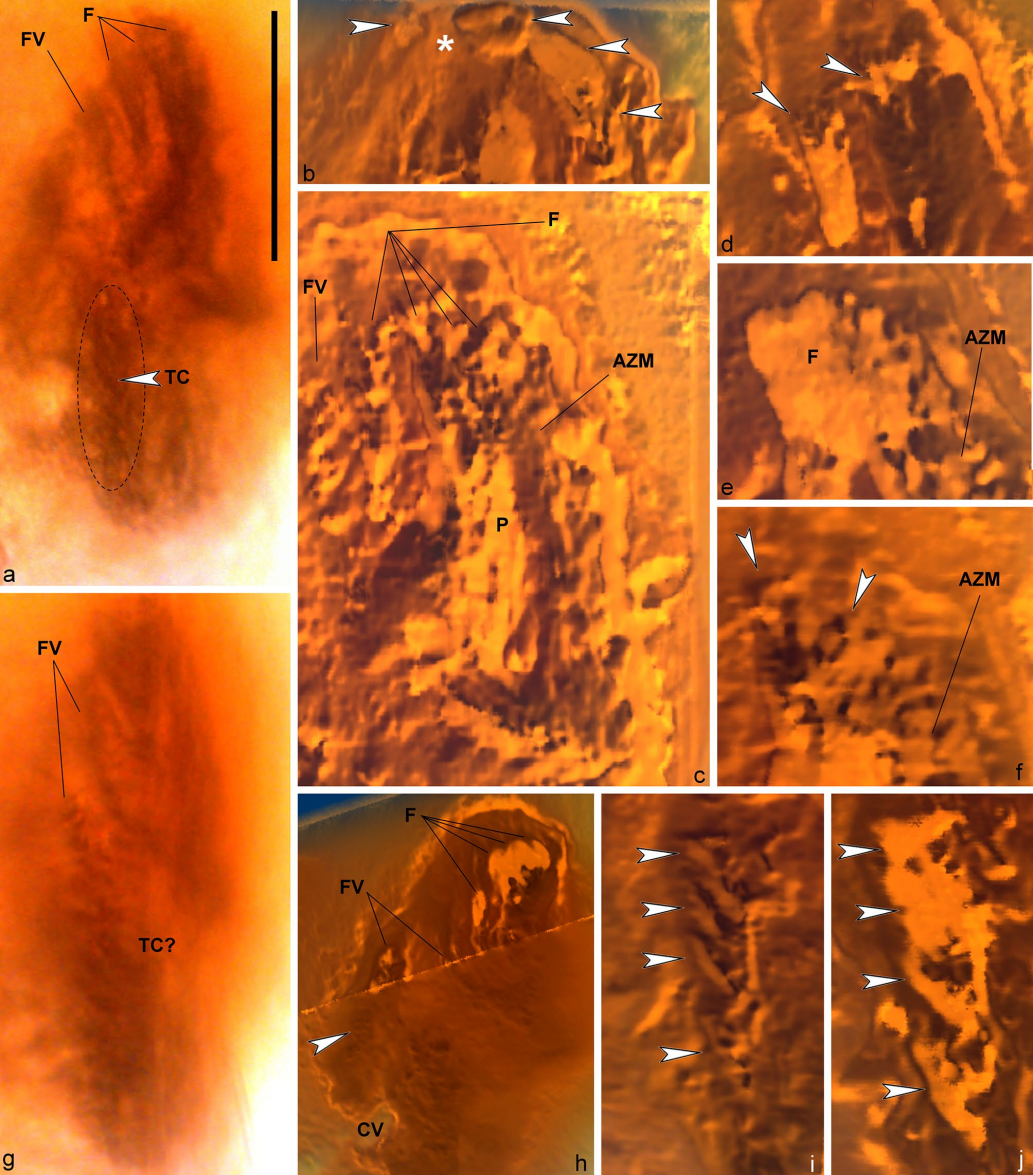
Light microscopy photograph (**a**) and volumetric reconstructions (**b–j**) of *Palaeohypothrix bahiensis* gen. *et* sp. nov. holotype. (**a**) Ventral side to show the transverse cirri-like structures (also in **i**,**j**) outlined. Arrowhead points to one of said structures. (**b**) Anterior region of body showing crown membranelles (arrowheads) and one intermembranellar ridge (asterisk). (**c**) Detail of right quadrant of peristome. Figure inset shows some insertion pores of ciliary structures forming the frontal brush immediately ahead of paroral. (**d–f**) Different focal planes of the anterior region to show frontal ciliature. Arrowheads indicate pits from where putative ciliary structures emerged. (**g**) Right lateral view. (**h**) Focal plane of the anterior region to show remnants of the frontal and fronto-ventral ciliature elements. Arrowhead shows insertion places of ciliary structures. (**i**,**j**) Insertion pits (i) and remnants of transverse cirri-like structures. Legends: *AZM* adoral zone of membranelles, *CV* contractile vacuole, *F* frontal ciliary structures, *FV* fronto-ventral ciliary structures, *P* paroral membrane, *TC(?)* possible transverse cirri-like structures. Scale bar = 20 µm.

**Figure 4:**
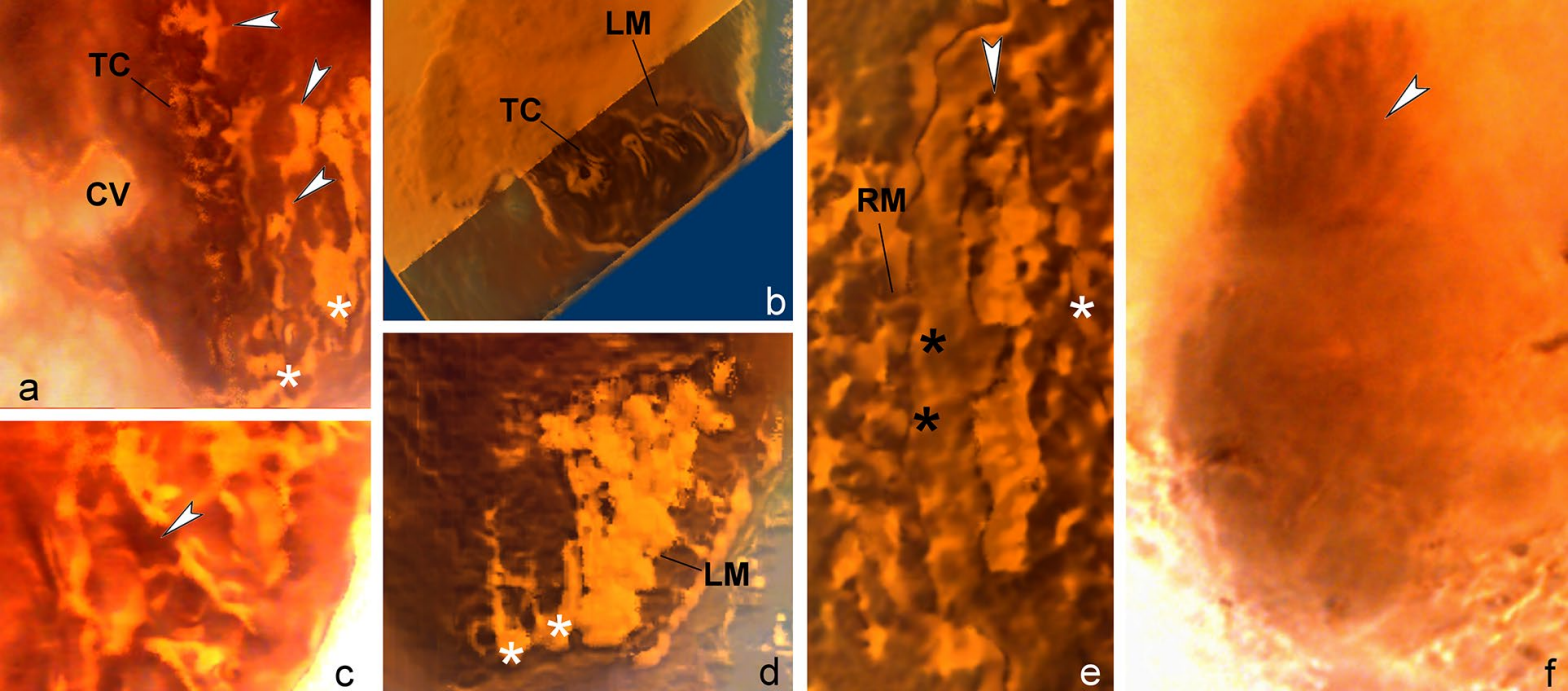
Volumetric reconstructions of *Palaeohypothrix bahiensis* gen. *et* sp. nov. holotype. (**a**) Posterior right quadrant of body showing undetermined surface structures (arrowheads) and part of the two ciliary brushes located between transverse cirri-like structures and left marginal ciliary brushes (asterisks). (**b**) Transversal section showing insertion of transverse cirri-like structures and left marginal ciliary brushes. (**c**) Insertion pores of left marginal ciliary elements (arrowhead). (**d**) Remnants of left marginal ciliature. Asterisks mark two ciliary brushes located between transverse cirri-like structures and left marginal rows set. (**e**) Detail of right margin of body. Black asterisks show putative marginal cirri; white asterisk shows supposed pellicular pores. Arrowhead points to rightmost ventral brush. (**f**) Dorsal side with arrowhead pointing to cortical ribs. Legends: *CV* contractile vacuole, *LM* left marginal ciliature, *RM* right marginal ciliature, *TC* transverse cirri-like structures.

The studied specimen differs from all known modern Spirotrichea for exhibiting the following combination of features: Body 45 × 30 µm, broadly elliptical, dorsoventrally flat about 2:1; contractile vacuole at posterior right quadrant of body; macronuclear DNA replication bands present; adoral zone of membranelles (AZM) distinctly separated in crown and lapel; paroral membrane (hereafter “paroral”) unusually complex, formed by multiple oblique segments adjacent to a long, thick file of cilia bordering a narrow peristome which occupies ca. 63% of body length; fronto-ventral-transverse (FVT) ciliature appearing mostly as anlagen-like brushes formed of tightly packed, roughly paired ciliary units. About four frontal brushes appearing contiguous with lapel adoral membranelles; distal end of paroral connecting with leftmost frontal brushes; two fronto-ventral brushes. About four transverse cirri-like structures as a row parallel to body length; left marginal ciliature formed of about ten oblique brushes of outwards decreasing length; right marginal ciliature as a cirral row; dorsal ribs present (Figs. 1, 2, 3, 4).

#### Remarks

As yet *Palaeohypothrix* is monotypic, therefore genus diagnosis is the same as for the type species *P. bahiensis*, and ditto that of Palaeohypotricha nov. tax., of which etymology borrows of the genus. The adopted name termination implies no formal Linnaean category^12^, but is meant to approximate subclass level.

### Morphology of the holotype

Possibly limnetic or terrestrial hypotrich *s. l*. Spirotrichea; body 45 × 30 µm, broad elliptical outline, narrowed anteriorly and slightly truncated at posterior end, dorsoventrally flat about 2:1, ventral surface roughly plane; dorsal convex (Fig. 1a–e). Surface (pellicle remnants?) apparently with numerous pores and undetermined structures; suggestive of extrusomes (Figs. 1b, 4e). Supposed contractile vacuole behind equatorial level of body, near right margin, with ventral overture (Figs. 1b,c, 2a,b, 4a).

Nuclear apparatus along midbody, composed of two roughly globular macronuclear nodules with supposed vestiges of chromatin condensations and discoid DNA replication bands. Anterior nodule 9.5 × 8.0 µm, 13.5 µm away from anterior end of body; posterior one 9.5 × 7.5 µm, ca. 2 µm away from the anterior nodule. At least one, ca. 1.5 µm in cross section, globular micronucleus attached to each macronuclear nodule (Figs. 1a, 2c,d).

Peristome occupying ca. 63% of body length; lapel and crown of AZM distinctly separated. Lapel falcate, containing ca. 35 membranelles; crown with about ten membranelles separate by inconspicuous ridges, possibly slightly overhanging lapel. Proximal lapel membranelles apparently sheathed in infundibulum; oral cavity narrow (Figs. 1b, 2a,e–g). Bases of widest membranelles about 7.5 µm wide. Putative paroral unusually complex, because formed by multiple oblique segments, adjacent to a long, thick (polystichomonad?) file of cilia adjacent to peristome border. Distal region of paroral appearing contiguous with leftmost frontal ciliature. Typical endoral membrane not recognized (Figs. 1b, 2f–i, 3b,c).

Fronto-ventral-transverse ciliature rather particular, because formed by ciliary units (indeterminable whether ordinary cilia or thin cirri) emerging from minute pits, roughly paired, arranged in tight rows resembling brushes (apparently anlagen-like; see Supplementary Discussion); exact number of ciliary units per row not determined (Figs. 1b, 2a, 3a,c–f). Frontal ciliature formed by apparently four of said brushes. Leftmost brushes (possibly three) hardly separable, appearing as a continuation of distal end of AZM lapel, possibly also connected posteriorly with paroral. Right frontal brush (i.e., fourth one) terminates at about the level of leftwards preceding frontal brush (Figs. 1b, 2i, 3c–f). Ventral region with two ciliary brushes: Leftmost one extending almost to body equator, wider anteriad, becomes thinner and composed of more spaced ciliary units towards distal end; right ventral brush running adjacent to left marginal row, possibly terminating slightly above the level of contractile vacuole (Figs. 2a, 3a,g,h, 4a).

At least four (five?) strong, transverse cirri-like structures emerging from conspicuous elongate, oblique pits longitudinally aligned below equatorial region of body, slightly displaced rightwards (Figs. 1b, 3g,i,j, 4b). Some undetermined surface structures present in the space between transverse cirri-like row and left marginal brush set (Figs. 1b, 4a). Left marginal ciliature with about ten ciliary brushes of decreasing length outwards, inserted in shallow furrows; outermost ones slightly oblique (Figs. 1b, 2a,f, 4e). One right marginal row, likely formed by cirri (Figs. 1b, 2a, 4b–d). Two short ciliary brushes (perhaps ventrally located caudal cirri-like structures) between transverse cirri-like structures and left marginal ciliature (Figs. 1b, 4a,d). Dorsal side with at least seven narrow cortical ribs slightly pronounced at anterior region of body, disappearing towards equatorial region of body (Figs. 1c, 4d). Dorsal ciliature not preserved. A tentative reconstruction of the live specimen is provided as Fig. 1d,e.

### Identification as a spirotrich ciliate

At first glance, the specimen roughly resembles a palynomorph because of its outline and size. However, this is readily refuted as one perceives the structures herein interpreted as remains of the nuclear apparatus (of which the anterior macronuclear nodule is the most evident) and of the AZM; both appearing concordant with “polyhymenophore” ciliates in shape and topology.

We identified the studied specimen as a spirotrich ciliate because it has both macro- and micronuclei (i.e., nuclear dualism), with morphological vestiges of discoid DNA replication bands in the macronuclear nodules. The former is a time-honored synapomorphy of the Ciliophora, and the latter of the Spirotrichea^1,13^. Moreover, the dorsoventrally flat body with putative remains of specialized ciliature concentrated on the ventral side, all indicate the specimen belongs to the hypotrichs *s. l*.^2^.

Among such structures, the nuclear apparatus is sometimes found in amber fossilized ciliates^14–16^, thus its preservation is not surprising. The DNA replication bands appear as conspicuous morphological features of the macronucleus in most spirotrichs, and are often easily visible under the light microscope even without the need of stain techniques (e.g., see Fig. 2 in Paiva et al.^17^). Hence, replication bands vestiges in fossilized macronuclear nodules are foreseeable, even though preservation of actual ancient DNA content is unlikely. Remarkably, recent findings, accompanied by experimental taphonomy results, suggest that fossilization of microeukaryote nuclei is more common than previously thought to be possible^18,19^. On the other hand, preservation of ciliary structures seems rare, as they tend degrade during resin embedding^14^, even though structures interpreted as flagella were found in inclusions identified as fossilized flagellates^16,20^.

### Comparison with related taxa

When compared to the main higher taxa of hypotrichs *s. l.* (viz., Discocephalida, Euplota, Hypotricha and Protohypotrichia), the absence of a prominent cephalization and the rightwards vertically aligned transverse cirri-like structures (vs. horizontally) likely exclude placement among the discocephalids^21,22^. Considering the remaining taxa, macronuclear DNA replication bands were not reported in protohypotrichs^2^, but regardless, the studied specimen presents notable protohypotrich features, as discussed further below. The nuclear apparatus composed of two ellipsoid nodules, associated with at least one small micronucleus is a recurrent feature among hypotrichs, and notably frequent in the “oxytrichids”, now assigned to the Postoralida^12^. On the other hand, the oral apparatus in the studied specimen is mostly reminiscent of the euplotids because of its large peristome to body length ratio and the presence of a multi rowed paroral membrane, which only barely resembles an euplotid polystichomonad pattern. In regard to this, the paroral configuration is worthy of attention for its uniqueness among the Spirotrichea. It represents a derived form due to its high complexity in relation not only to modern spirotrichs^21,23–25^, but also to the SAL supergroup, to where they belong^26^. Another possibility which cannot be disregarded is that the multiple oblique fragments interpreted as part of the paroral do not belong to such structure, but could actually be homologous to the buccal cirri of modern hypotrichs^3,27–29^. The absence of the endoral membrane, although not confirmed, is another common feature in euplotids, except for the diophryines^3,30^. However, the right portion of the complex paroral structure could be an atypical endoral. Unfortunately, shedding light on such matters would require investigation of ontogenetic processes.

Many euplotids and some protohypotrichs typically have the contractile vacuole positioned in the right side of body, below the equatorial region, as in the present specimen^31,32^. Conversely, in hypotrichs such vacuole(s) is(are) usually positioned along the left side of body^3,27–29^. The contractile vacuole in the present specimen was interpreted as such due to its position matching with those of euplotids, including the ventral overture. It cannot be excluded, however, that said structure is a cytoproct preserved during excretion. Anyhow, a fecal pellet was not found anywhere near the specimen, thus such interpretation is less favored.

The ventral ciliary pattern of the studied specimen has a unique feature among the Spirotrichea: Its basic organization displaying brushes of ciliary units at specific regions. Such pattern, and the connection of the paroral to the frontal brushes, are all reminiscent of the proter fronto-ventral-transverse (FVT) anlagen set of some modern hypotrichs, as developing during middle stage morphogenesis^33–35^. However, overall evidence seems to favor the interpretation of the specimen as a morphostatic cell: While the macronuclear replication bands indicate at least a very early stage of divisional morphogenesis, the absence of opisthe stomatogenesis (which normally precedes visible FVT anlagen differentiation)^27,36^ demonstrates the ciliature was still morphostatic when the ciliate was entrapped by the resin. It is worth to mention that we cannot completely exclude the possibility of the structures interpreted as the left marginal brushes being the oral anlage of the opisthe. However, the ciliary brushes and their insertion furrows can be distinguished, at least partially, either by direct observation under bright field or after reconstructions, and they appear different from typical euplotid (subsurface) or hypotrich (surface) opisthe oral anlage.

Physiological reorganization could also explain proter-like FVT anlagen, but it is overruled because macronuclear replication bands are uncommon during that process^37^. Therefore, a suitable explanation for the curious ventral ciliature pattern of the studied specimen could be neoteny, because it exhibits anlage-like organization of most structures in a morphostatic, mature cell. Said pattern is, perhaps, comparable to that of the protohypotrich *Kiitricha marina*, in which anlagen segregation into cirri is belated, occurring after cytokinesis^2^. However, contrarily to that species, the juvenile pattern of the ciliature in *P. bahiensis* was retained during interphase, to the point it was still present when the macronuclear replication bands appeared—hence the neoteny. Lastly regarding the FVT ciliature, the longitudinal alignment of the transverse cirri-like structures is also reminiscent of protohypotrichs^2,31,38–41^. Noteworthy, we observed some undetermined structures on the ventral surface, in the space between the transverse cirri-like structures and the left marginal brushes. Although suggestive of remains of scattered ciliary elements or perhaps extrusomes, they did not conform with the criteria used to discern actual anatomic features from artifacts (see “Methods”). Therefore, were not included in the live specimen reconstruction presented in Fig. 1d,e.

The presence of ciliary rows on both right and left body margins is consistent with all hypotrich *s. l*. groups, except euplotids, which normally lack the right marginal and display reduced number of left marginal cirri^1,32^. In protohypotrichs, possible right marginal cirri are not distinguishable from ventral ones, and the left marginal cirri are not ontogenetically homologous to those of the other groups^2^. Nevertheless, the arrangement of the left marginal ciliature of the studied specimen as brushes is also unique among hypotrichs *s. l.*

Dorsally, the specimen has cortical ribs, indicating it had a rigid pellicle. Cortical ribs are common in various euplotids^42,43^, but not in hypotrichs, albeit exceptions occur, such as in *Hemiholosticha kahli*^44^. It is worthy of note that the posterior shortening of dorsal ribs could be an artifact of preservation in the studied specimen, therefore, should not be over interpreted.

Although comparable fossil hypotrichs *s. l.* are presently unknown, a last common ancestor of the Hypotricha was hypothesized by Berger^3^ as resembling a typical non-dorsomarginalian 18-FVT cirri Hypotricha, thus being remarkably different from *P. bahiensis*.

### Phylogenetic position and systematic implications to the Spirotrichea

**Figure 5:**
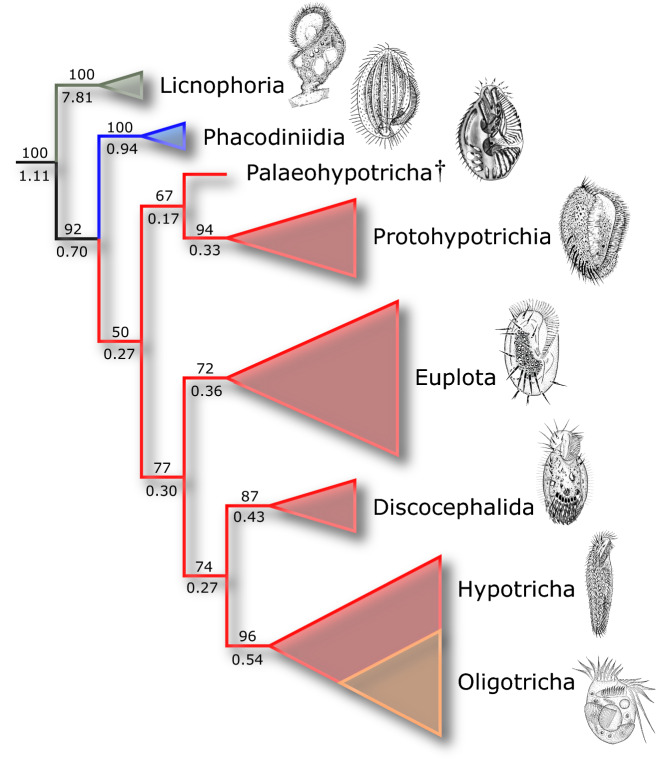
Simplified cladogram of the Spirotrichea showing resolution at higher taxa nodes, including the putatively extinct Palaeohypotricha nov. tax. Score (*k* = 4.765625) 189.07802; consistency index (CI) = 0.44; retention index (RI) = 0.51. The hypotrichs *s. l*. branches are shaded in red. Numbers above and below branches are symmetric resampling GC frequencies and Bremmer support values, respectively. Branches without numbering are monotypic. Outgroup omitted. Illustrative figures from top to bottom (modified from original sources listed in Supplementary Table S1, Supplementary Materials): *Licnophora lyngbycola* (~ 150 µm); *Phacodinium metchnikoffi* (~ 100 µm); *Palaeohypothrix bahiensis* (~ 45 µm); *Kiitricha marina* (~ 100 µm); *Euplotes aediculatus* (~ 128 µm); *Discocephalus ehrenbergi* (~ 110 µm); *Holosticha heterofoissneri* (~ 141 µm); *Propecingulum fistoleramalliei* (~ 55 µm).

A cladistic analysis of combined morphological and molecular characters (Supplementary Materials and Supplementary Data 2) resulted in one optimal cladogram (Fig. 5; Supplementary Fig. S1) in which the inclusion/exclusion of *P. bahiensis* from the analysis did not alter the resulting topology. The phylogenetic pattern is largely in agreement with previous studies, considering that the position and sometimes the monophyly of the main taxa vary slightly^4,5,45^. The placement of *P. bahiensis* (and consequently of the Palaeohypotricha) as sister of the marine Protohypotrichia is sustained by two morphological synapomorphies, namely: (i) the rightwards inclination of transverse cirri, tending to be verticalized—a pattern which also evolved independently in some euplotids, such as *Aspidisca*^32^; and (ii) persistence of ventral ciliature elements as anlagen-like structures after cytokinesis. The latter is transient in Protohypotrichia, since the all FVT anlagen eventually differentiate into cirri as postdividers maturate^2,39^. However, in *P. bahiensis* the fronto-ventral and the whole left marginal ciliature were retained as anlagen-like structures during interphase, because they occur concomitantly with the macronuclear replication bands. Hence, such ciliary structures are possibly neotenic. Traits such as (i) the complex arrangement of the paroral and its connection with the left most elements of the frontal ciliature; (ii) the continuity of the adoral membranelles of the lapel with the frontal ciliature; and (iii) the permanence of fronto-ventral and left marginal ciliary elements in anlage-like brushes during interphase are thus considered autapomorphies of *P. bahiensis,* and possible synapomorphies of Palaeohypotricha nov. tax. These are clearly ground plan defining traits not found in the more than 1300 known species of hypotrichs *s. l*.^46,47^, hence warranting the taxonomic acts herein established and the reasonable assumption of Palaeohypotricha as a putatively extinct ciliate higher taxon lineage.

## Methods

### Geological and paleobotanical context

The fossil analysed in this study is an amber inclusion found in fine sandstones of the Maracangalha Formation from the Recôncavo Basin (Frade Island, Bahia state), Northeastern Brazil. This lithostratigraphic unit was deposited in the geological context of Gondwana break up and first stages of Atlantic Ocean opening^48^. The depositional environments were under gravitational flows during the syn-rift phase of the Recôncavo Basin. The sediments of Maracangalha Formation are fine sandstones, siltstones and laminated mudstones with deformational structures attributed to the gravitational flows^49^. This lithostratigraphic unit is subdivided in the Caruaçu and Pitanga members. The amber samples came from fine sandstones and shales of the Caruaçu Member, considered as Berriasian–Barremian age, in a time interval of 145–125 Mya^50,51^. The sedimentary succession of the Caruaçu Member is interpreted as channel-fill, proximal levee facies and sheet sands in a deltaic complex of a lacustrine environment^49^.

The paleobotanical affinity of this amber based on the record of fossil plants and pollen is difficult, as hitherto there are no well documented studies on paleobotany on the Caruaçu Member. Gas chromatography-mass spectrometry (GC/MS) analysis from the extracts of this amber identified alkylbenzenes, alkylnaphtalenes, alkylhydronaphtalenes, parafins, phenols, carboxylic acids and terpenoids^52^. Based on biomarkers as fenchone, camphor, 16, 17, 19-trisnorabieta-8, 11, 13-triene and methyl 16, 17- bisnordehydroabietate, the botanical origin should be related to Araucariaceae^52^. Therefore, later studies on the chemical composition of this amber^50,53^ indicated that there are phyllocladanes and kauranes, also chemosystematic markers of other plant families besides Araucariaceae. The original resins could also be produced by plants from the Cupressaceae or Podocarpaceae families, but excludes Pinaceae. As triterpenoid compounds were not found, most angiosperms are not to be considered as the source of the resin.

Preservation quality of microscopic eukaryotes in amber may vary depending on the botanical origin of resin^14^, but other factors, in special temperature when embedded, appear to be determinant in structural preservation^54^. The resin in which the studied specimen was embedded stained the cellular structures we interpreted as the remains of nuclear apparatus and ciliature elements in the fashion of a faint protargol preparation—a rare event, herein reported for the first time in a ciliate amber inclusion.

### Morphology and taxonomy

Amber fragments of roughly 1 cm^3^ were cut in slices of about the thickness of a typical microscope glass slide and polished on both sides. Four slices were then searched for inclusions suggestive of microeukaryotes. To do so, the slides were covered with a water droplet to reduce the mirror effect caused by microscopic irregularities and cracks, and a coverslip for observation under the light microscope. We measured and photographed the studied specimen under bright field, 1.000 × (oil immersion), on various focal planes, based on which volume reconstructions were performed with Volume Viewer 2.01 plugin in Fiji^55^, using z-aspect of 2.0, varying alpha offsets, with and without oblique illumination. When applying the oblique illumination effect, presumed vestiges of ciliary structures appeared as cloudy masses at different focal planes. To discern from potential artifacts, we considered valid only those structures which (i) could be detected from different angles and were traceable through focal planes; (ii) were consistent with observations made under bright field; and (iii) homology with modern ciliates could be determined. The photographs used to make volume reconstructions are available as Supplementary Data 1. An interpretative reconstruction of the live specimen was made based on our observations of the fossil structures and comparison with related modern organisms.

Terminology of spirotrich ciliate morphology is basically according to Berger^3,28^ and systematics above subclass level follows Lynn^1^. Given the potentially misleading effect of excessive scale bars when fixation shrinkage and other preparation artifacts alter the actual size of cell structures in modern ciliates^56^, we limited the use of scale bars to avoid informing false precision due to size distortions after volume reconstruction, preferring to provide measurement values of taxonomically relevant structures in the main text.

### Cladistic analysis

Taxon sample contained species representing the Spirotrichea higher taxa, plus two species of *Blepharisma* (Heterotrichea) as outgroup, totalizing 29 terminals with *P. bahiensis* included (Supplementary Materials, Supplementary Table S1). We analysed a matrix of 26 morphological characters combined with the 18S rDNA (of modern species). Morphological characters were proposed as statements^57^, of which primary homologies rely on the interpretation of the studied specimen as a spirotrich ciliate. Characters were coded as either binary or multistate, treated as unordered. Issues of non-applicable character states were resolved via contingent coding^58,59^. The 18S matrix was aligned through the MUSCLE algorithm implemented in MEGA X^60^, and refined manually to minimize changes among nucleotides; leading and trailing gaps coded as missing data.

A cladistic analysis was performed in TNT 1.5^61^ with parsimony uninformative characters disabled and gaps treated as a fifth base. We analysed the combined matrix under extended implied weights regime^62^, selecting the value of Goloboff´s concavity constant *k* with the *setk.run* script written by Salvador Arias^63^. To search for optimal cladograms, we employed a combination of parsimony-ratchet, tree-drifting, tree-fusing and sectorial search routines^64,65^. Clade support was evaluated via 1000 symmetric resamples, expressed as GC frequencies (a metric which ranges from − 100 to 100), for which values < 0 were considered unreliable^66,67^, and the traditional Bremer index^68,69^, given in fit units to ensure consistency with analysis optimality criterion. Moreover, the ensemble consistency (CI) and retention (RI) indexes were calculated to measure character logical consistency and synapomorphy retention onto the tree, respectively^58^. Root placement was made *a posteriori*^70^*.* The analysed matrix is provided as a TNT formatted file (Supplementary Data 2).

## Supplementary Information

Supplementary Information 1.

Supplementary Information 2.

Supplementary Information 3.

## Data Availability

The analysed data used in this study are available as part of the Article and Supplementary Materials. The new genus and species in publication have been registered at ZooBank (LSID: D98CA5A2-B5B4-4264-B703-1EDE1EFF22BF).
