# Supplementary figures and images for: A putatively extinct higher taxon of Spirotrichea (Ciliophora) from the Lower Cretaceous of Brazil
Source: Sci Rep. 2021 Sep 27;11:19110. doi: 10.1038/s41598-021-97709-2 (PMC8476538; doi:10.1038/s41598-021-97709-2)

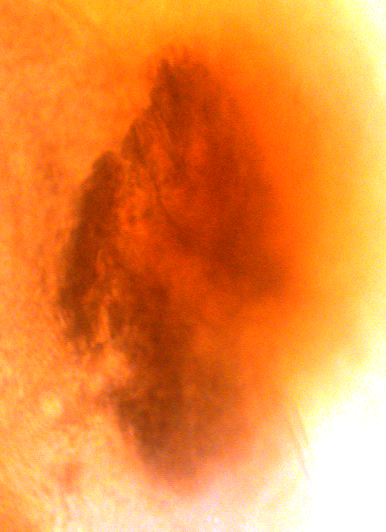

Supplement: Supplementary file 1 — Supplementary Information 1. [file 41598_2021_97709_MOESM1_ESM.zip › ufrjdg762pb/ufrjdg762pb0d0000.png]

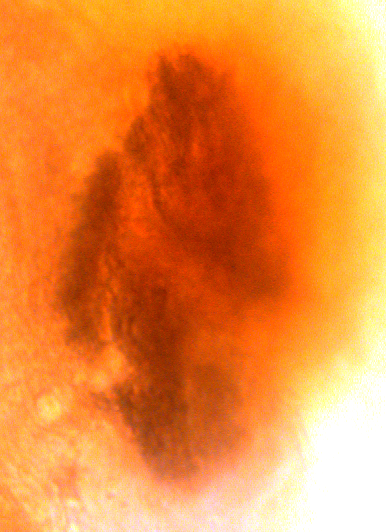

Supplement: Supplementary file 1 — Supplementary Information 1. [file 41598_2021_97709_MOESM1_ESM.zip › ufrjdg762pb/ufrjdg762pb0d0001.png]

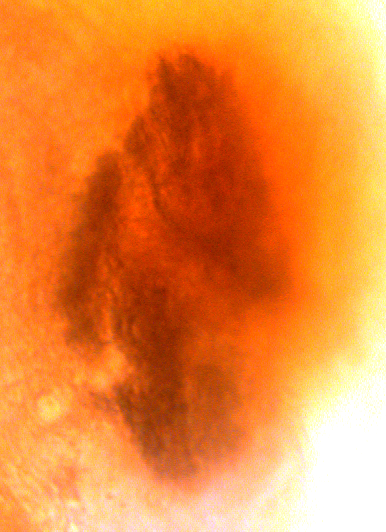

Supplement: Supplementary file 1 — Supplementary Information 1. [file 41598_2021_97709_MOESM1_ESM.zip › ufrjdg762pb/ufrjdg762pb0d0002.png]

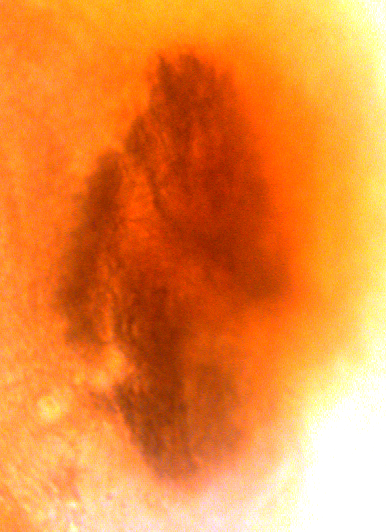

Supplement: Supplementary file 1 — Supplementary Information 1. [file 41598_2021_97709_MOESM1_ESM.zip › ufrjdg762pb/ufrjdg762pb0d0003.png]

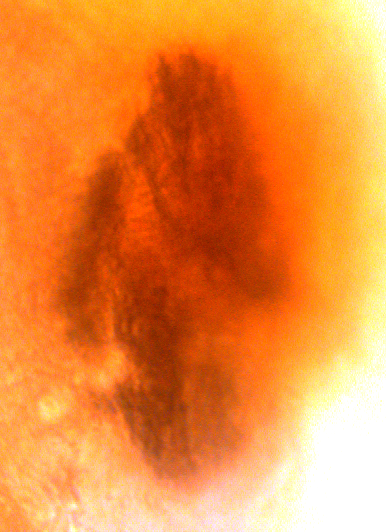

Supplement: Supplementary file 1 — Supplementary Information 1. [file 41598_2021_97709_MOESM1_ESM.zip › ufrjdg762pb/ufrjdg762pb0d0004.png]

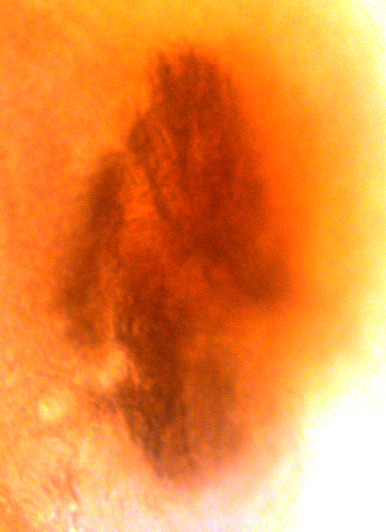

Supplement: Supplementary file 1 — Supplementary Information 1. [file 41598_2021_97709_MOESM1_ESM.zip › ufrjdg762pb/ufrjdg762pb0d0005.png]

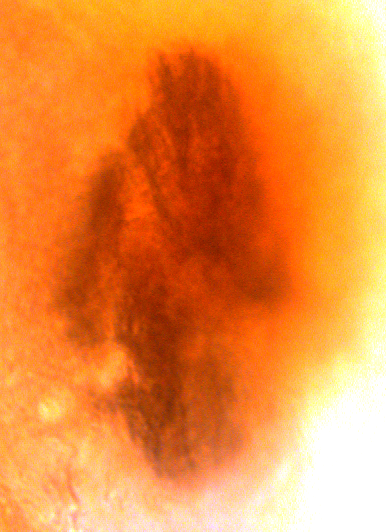

Supplement: Supplementary file 1 — Supplementary Information 1. [file 41598_2021_97709_MOESM1_ESM.zip › ufrjdg762pb/ufrjdg762pb0d0006.png]

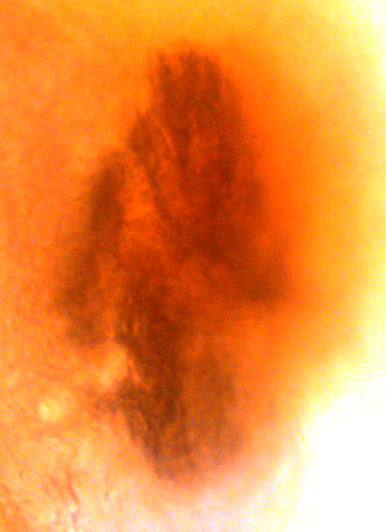

Supplement: Supplementary file 1 — Supplementary Information 1. [file 41598_2021_97709_MOESM1_ESM.zip › ufrjdg762pb/ufrjdg762pb0d0007.png]

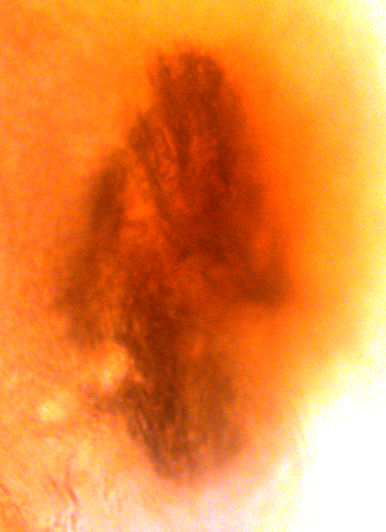

Supplement: Supplementary file 1 — Supplementary Information 1. [file 41598_2021_97709_MOESM1_ESM.zip › ufrjdg762pb/ufrjdg762pb0d0008.png]

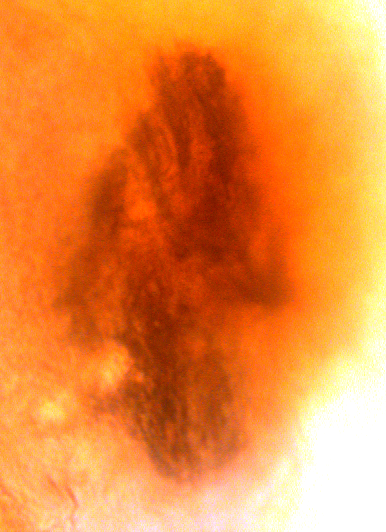

Supplement: Supplementary file 1 — Supplementary Information 1. [file 41598_2021_97709_MOESM1_ESM.zip › ufrjdg762pb/ufrjdg762pb0d0009.png]

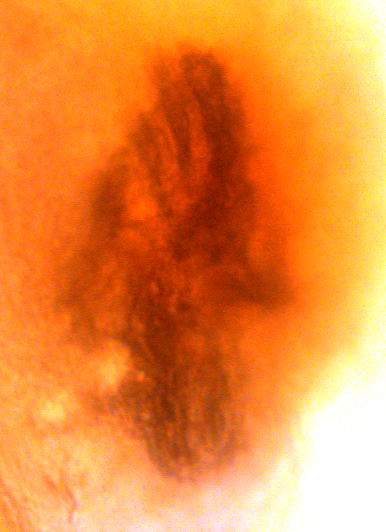

Supplement: Supplementary file 1 — Supplementary Information 1. [file 41598_2021_97709_MOESM1_ESM.zip › ufrjdg762pb/ufrjdg762pb0d0010.png]

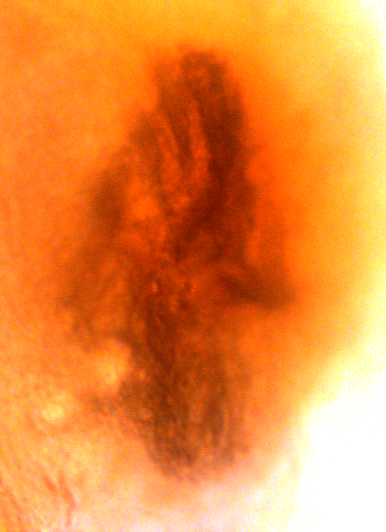

Supplement: Supplementary file 1 — Supplementary Information 1. [file 41598_2021_97709_MOESM1_ESM.zip › ufrjdg762pb/ufrjdg762pb0d0011.png]

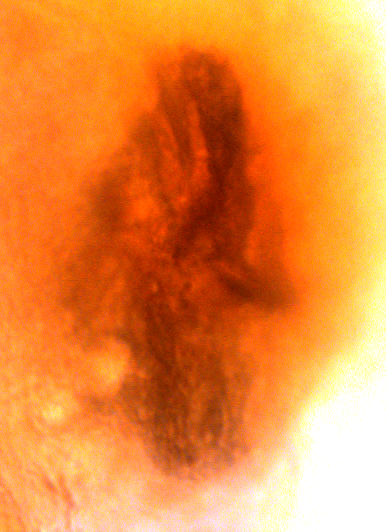

Supplement: Supplementary file 1 — Supplementary Information 1. [file 41598_2021_97709_MOESM1_ESM.zip › ufrjdg762pb/ufrjdg762pb0d0012.png]

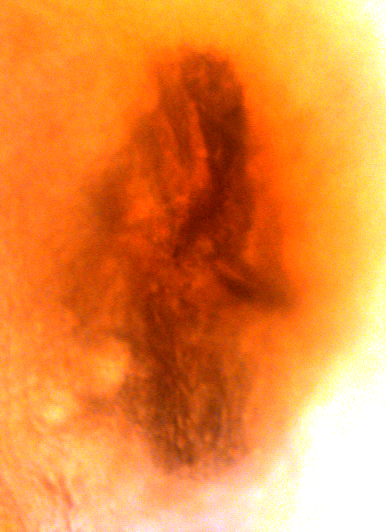

Supplement: Supplementary file 1 — Supplementary Information 1. [file 41598_2021_97709_MOESM1_ESM.zip › ufrjdg762pb/ufrjdg762pb0d0013.png]

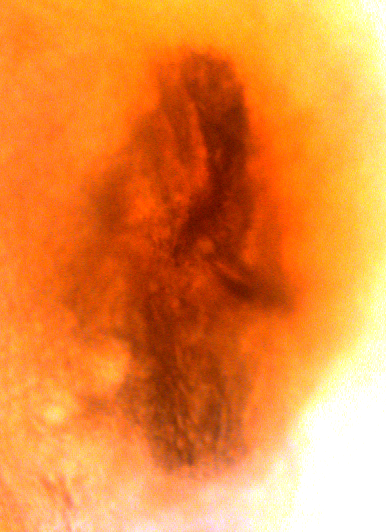

Supplement: Supplementary file 1 — Supplementary Information 1. [file 41598_2021_97709_MOESM1_ESM.zip › ufrjdg762pb/ufrjdg762pb0d0014.png]

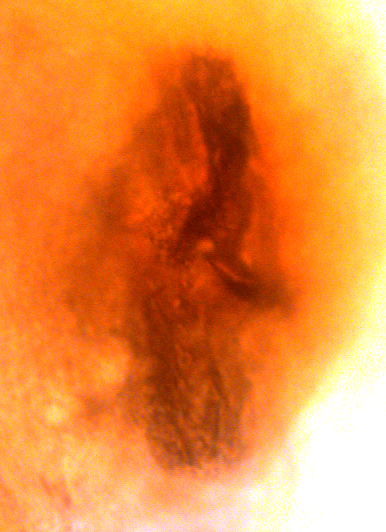

Supplement: Supplementary file 1 — Supplementary Information 1. [file 41598_2021_97709_MOESM1_ESM.zip › ufrjdg762pb/ufrjdg762pb0d0015.png]

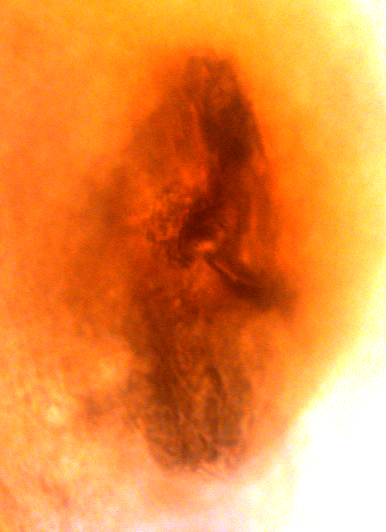

Supplement: Supplementary file 1 — Supplementary Information 1. [file 41598_2021_97709_MOESM1_ESM.zip › ufrjdg762pb/ufrjdg762pb0d0016.png]

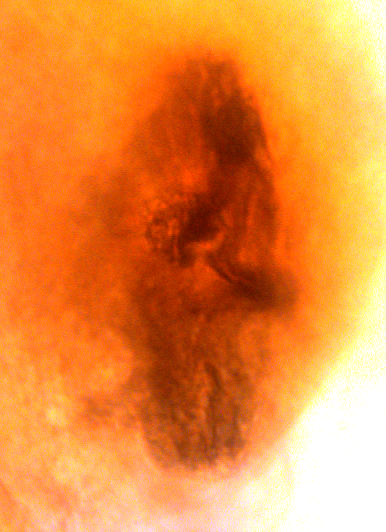

Supplement: Supplementary file 1 — Supplementary Information 1. [file 41598_2021_97709_MOESM1_ESM.zip › ufrjdg762pb/ufrjdg762pb0d0017.png]

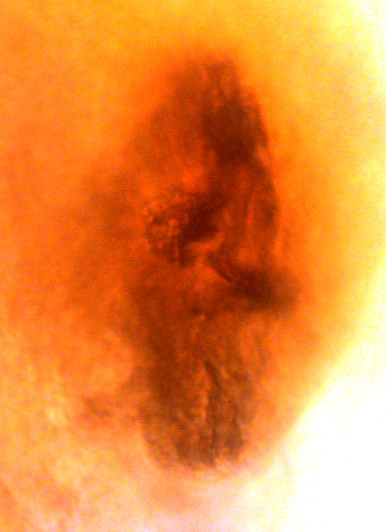

Supplement: Supplementary file 1 — Supplementary Information 1. [file 41598_2021_97709_MOESM1_ESM.zip › ufrjdg762pb/ufrjdg762pb0d0018.png]

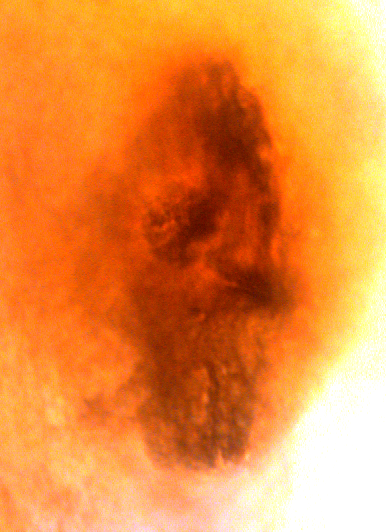

Supplement: Supplementary file 1 — Supplementary Information 1. [file 41598_2021_97709_MOESM1_ESM.zip › ufrjdg762pb/ufrjdg762pb0d0019.png]

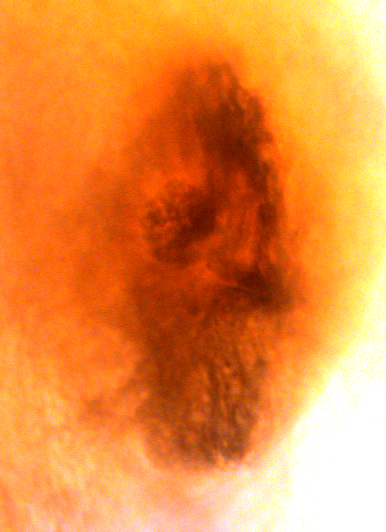

Supplement: Supplementary file 1 — Supplementary Information 1. [file 41598_2021_97709_MOESM1_ESM.zip › ufrjdg762pb/ufrjdg762pb0d0020.png]

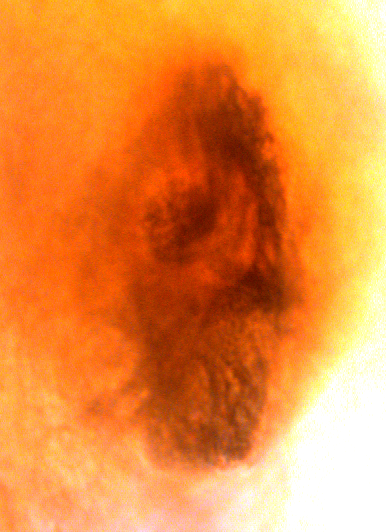

Supplement: Supplementary file 1 — Supplementary Information 1. [file 41598_2021_97709_MOESM1_ESM.zip › ufrjdg762pb/ufrjdg762pb0d0021.png]

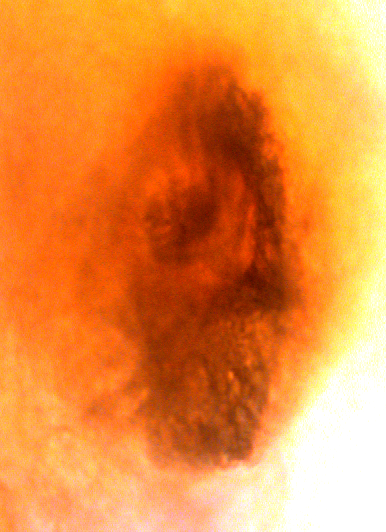

Supplement: Supplementary file 1 — Supplementary Information 1. [file 41598_2021_97709_MOESM1_ESM.zip › ufrjdg762pb/ufrjdg762pb0d0022.png]

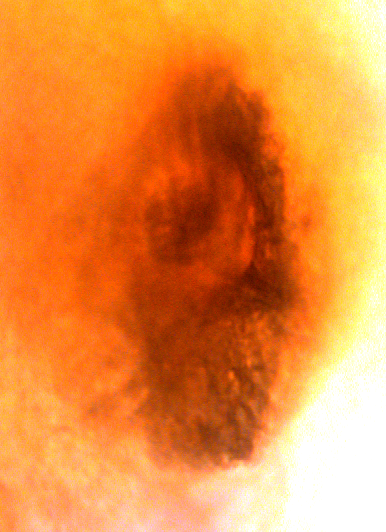

Supplement: Supplementary file 1 — Supplementary Information 1. [file 41598_2021_97709_MOESM1_ESM.zip › ufrjdg762pb/ufrjdg762pb0d0023.png]

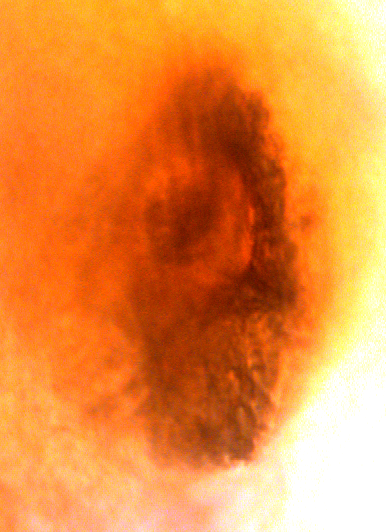

Supplement: Supplementary file 1 — Supplementary Information 1. [file 41598_2021_97709_MOESM1_ESM.zip › ufrjdg762pb/ufrjdg762pb0d0024.png]

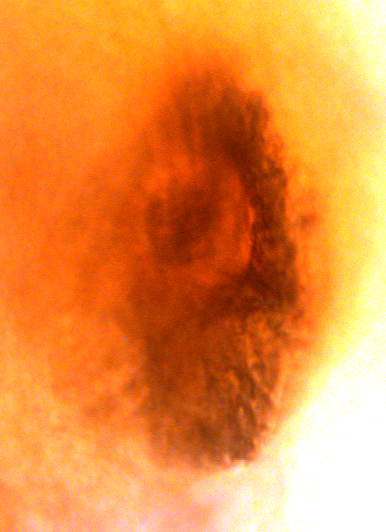

Supplement: Supplementary file 1 — Supplementary Information 1. [file 41598_2021_97709_MOESM1_ESM.zip › ufrjdg762pb/ufrjdg762pb0d0025.png]

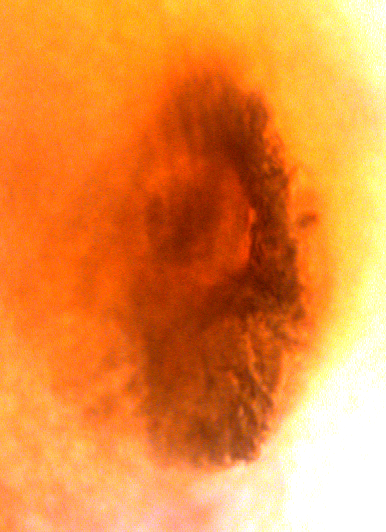

Supplement: Supplementary file 1 — Supplementary Information 1. [file 41598_2021_97709_MOESM1_ESM.zip › ufrjdg762pb/ufrjdg762pb0d0026.png]

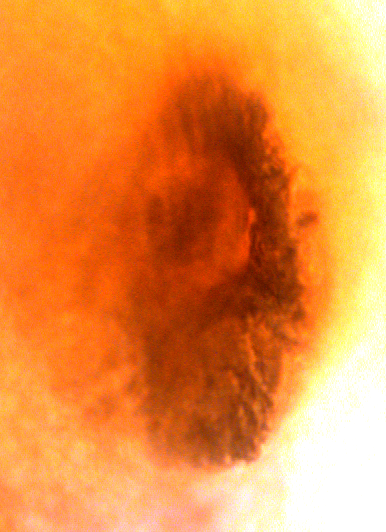

Supplement: Supplementary file 1 — Supplementary Information 1. [file 41598_2021_97709_MOESM1_ESM.zip › ufrjdg762pb/ufrjdg762pb0d0027.png]

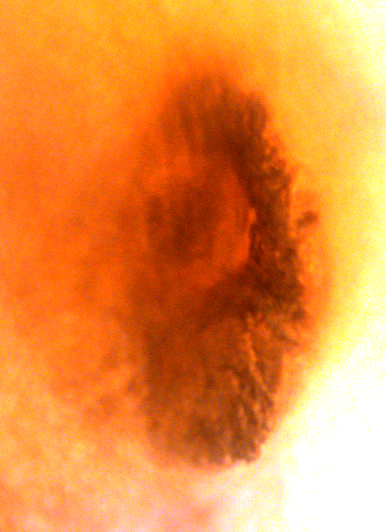

Supplement: Supplementary file 1 — Supplementary Information 1. [file 41598_2021_97709_MOESM1_ESM.zip › ufrjdg762pb/ufrjdg762pb0d0028.png]

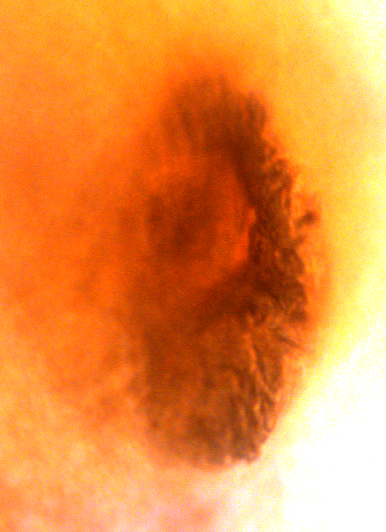

Supplement: Supplementary file 1 — Supplementary Information 1. [file 41598_2021_97709_MOESM1_ESM.zip › ufrjdg762pb/ufrjdg762pb0d0029.png]

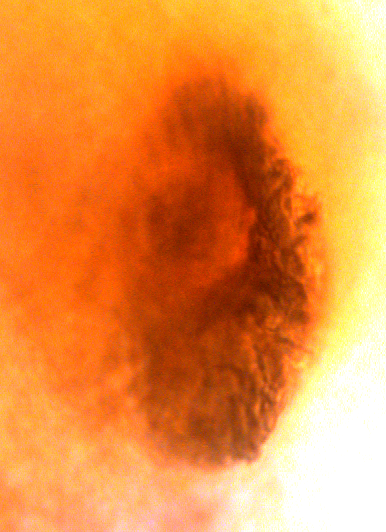

Supplement: Supplementary file 1 — Supplementary Information 1. [file 41598_2021_97709_MOESM1_ESM.zip › ufrjdg762pb/ufrjdg762pb0d0030.png]

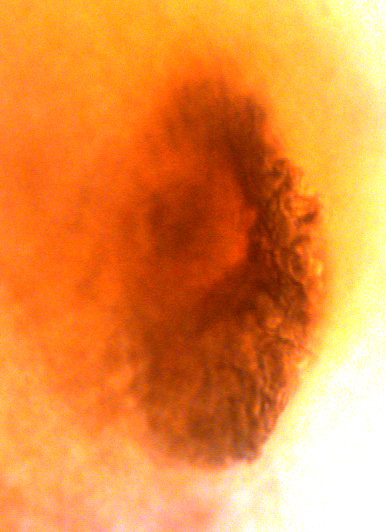

Supplement: Supplementary file 1 — Supplementary Information 1. [file 41598_2021_97709_MOESM1_ESM.zip › ufrjdg762pb/ufrjdg762pb0d0031.png]

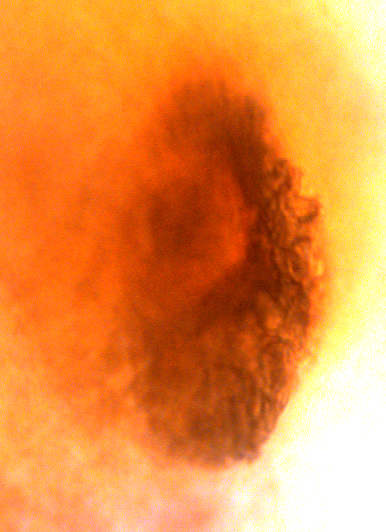

Supplement: Supplementary file 1 — Supplementary Information 1. [file 41598_2021_97709_MOESM1_ESM.zip › ufrjdg762pb/ufrjdg762pb0d0032.png]

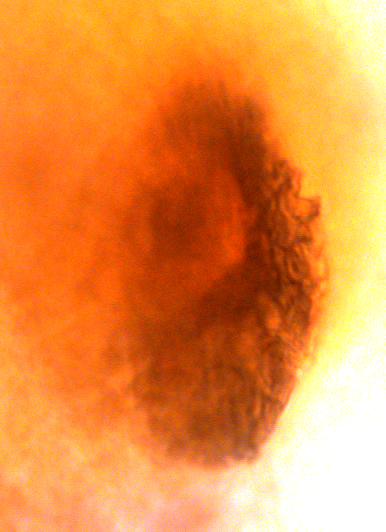

Supplement: Supplementary file 1 — Supplementary Information 1. [file 41598_2021_97709_MOESM1_ESM.zip › ufrjdg762pb/ufrjdg762pb0d0033.png]

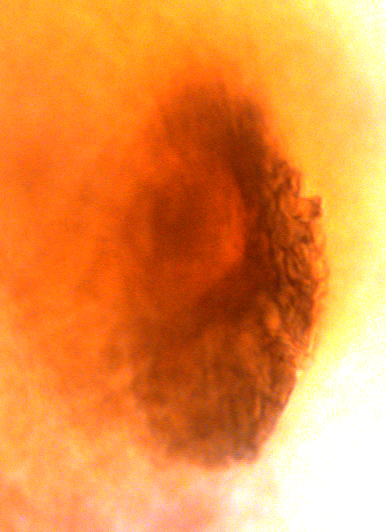

Supplement: Supplementary file 1 — Supplementary Information 1. [file 41598_2021_97709_MOESM1_ESM.zip › ufrjdg762pb/ufrjdg762pb0d0034.png]

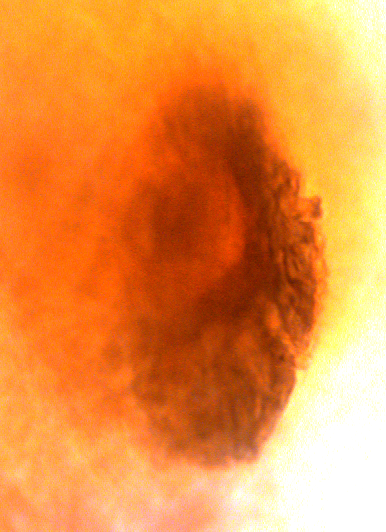

Supplement: Supplementary file 1 — Supplementary Information 1. [file 41598_2021_97709_MOESM1_ESM.zip › ufrjdg762pb/ufrjdg762pb0d0035.png]

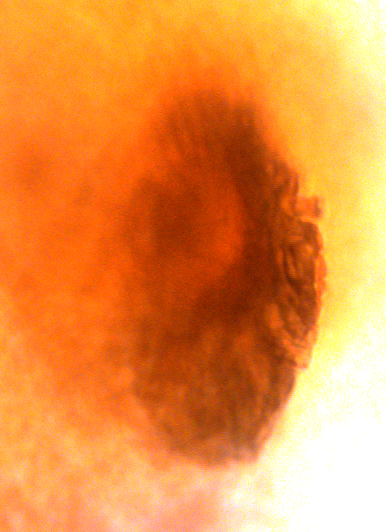

Supplement: Supplementary file 1 — Supplementary Information 1. [file 41598_2021_97709_MOESM1_ESM.zip › ufrjdg762pb/ufrjdg762pb0d0036.png]

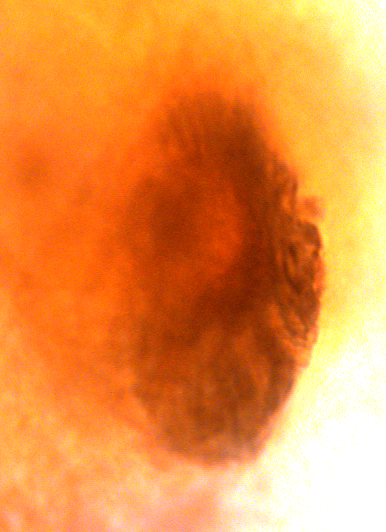

Supplement: Supplementary file 1 — Supplementary Information 1. [file 41598_2021_97709_MOESM1_ESM.zip › ufrjdg762pb/ufrjdg762pb0d0037.png]

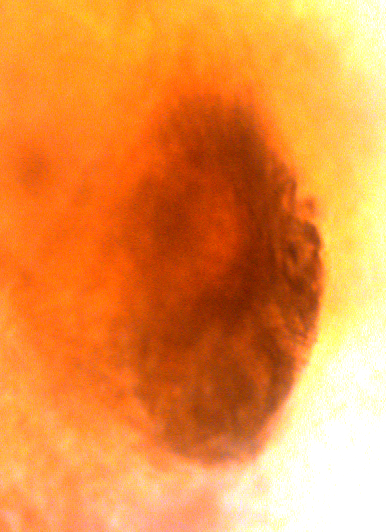

Supplement: Supplementary file 1 — Supplementary Information 1. [file 41598_2021_97709_MOESM1_ESM.zip › ufrjdg762pb/ufrjdg762pb0d0038.png]

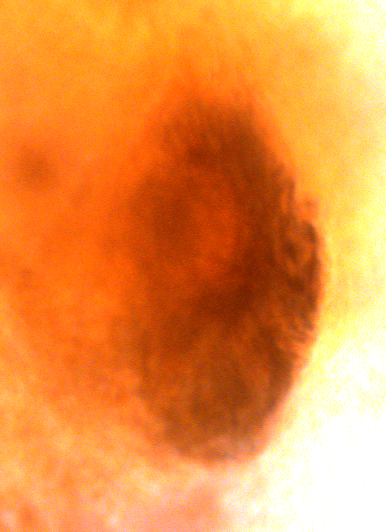

Supplement: Supplementary file 1 — Supplementary Information 1. [file 41598_2021_97709_MOESM1_ESM.zip › ufrjdg762pb/ufrjdg762pb0d0039.png]

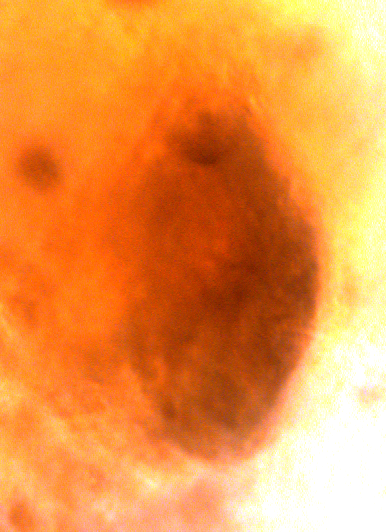

Supplement: Supplementary file 1 — Supplementary Information 1. [file 41598_2021_97709_MOESM1_ESM.zip › ufrjdg762pb/ufrjdg762pb0d0040.png]

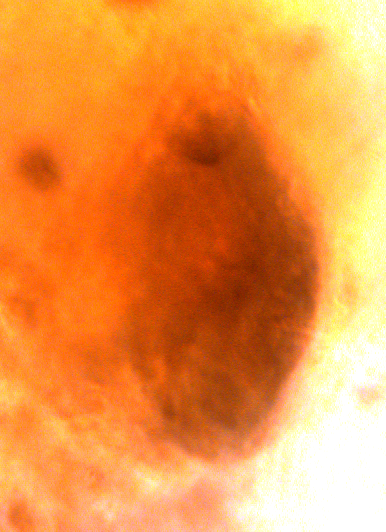

Supplement: Supplementary file 1 — Supplementary Information 1. [file 41598_2021_97709_MOESM1_ESM.zip › ufrjdg762pb/ufrjdg762pb0d0041.png]

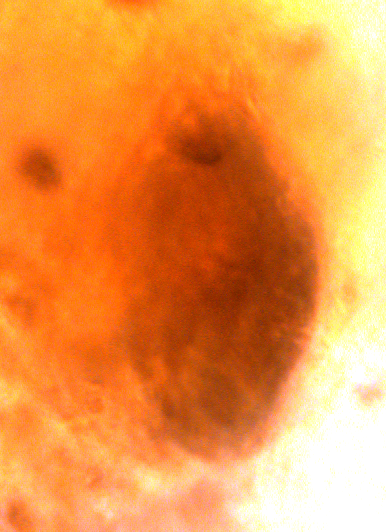

Supplement: Supplementary file 1 — Supplementary Information 1. [file 41598_2021_97709_MOESM1_ESM.zip › ufrjdg762pb/ufrjdg762pb0d0042.png]

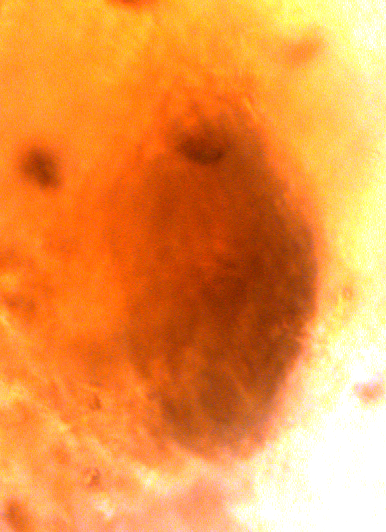

Supplement: Supplementary file 1 — Supplementary Information 1. [file 41598_2021_97709_MOESM1_ESM.zip › ufrjdg762pb/ufrjdg762pb0d0043.png]

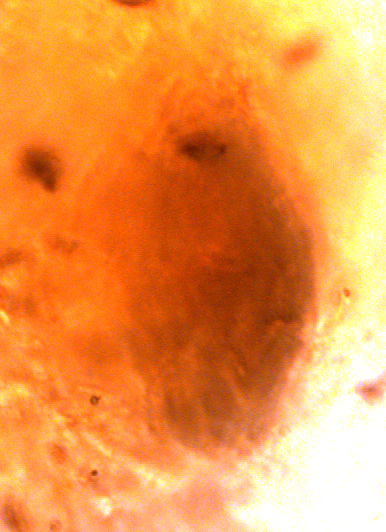

Supplement: Supplementary file 1 — Supplementary Information 1. [file 41598_2021_97709_MOESM1_ESM.zip › ufrjdg762pb/ufrjdg762pb0d0044.png]

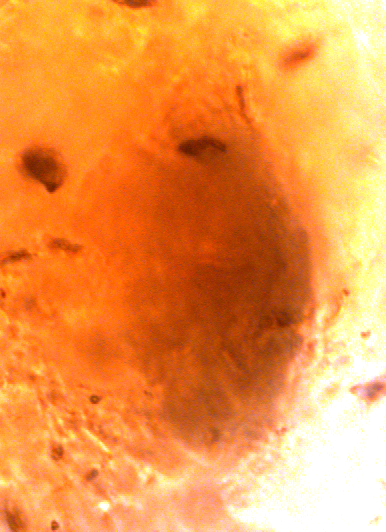

Supplement: Supplementary file 1 — Supplementary Information 1. [file 41598_2021_97709_MOESM1_ESM.zip › ufrjdg762pb/ufrjdg762pb0d0045.png]

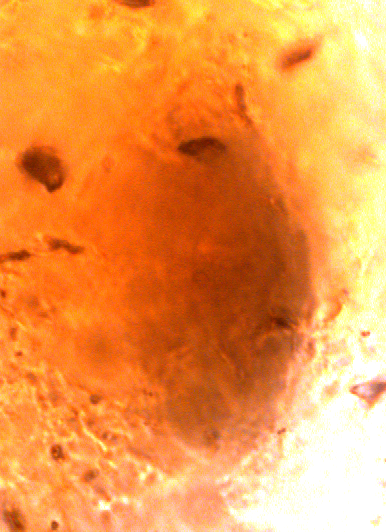

Supplement: Supplementary file 1 — Supplementary Information 1. [file 41598_2021_97709_MOESM1_ESM.zip › ufrjdg762pb/ufrjdg762pb0d0046.png]
