# Supplementary material for: A putatively extinct higher taxon of Spirotrichea (Ciliophora) from the Lower Cretaceous of Brazil
Source: Sci Rep. 2021 Sep 27;11:19110. doi: 10.1038/s41598-021-97709-2 (PMC8476538; doi:10.1038/s41598-021-97709-2)
Supplement: Supplementary file 2 — Supplementary Information 2. [file 41598_2021_97709_MOESM2_ESM.pdf]

nstates cont;

nstates 32;

xread 'Supplementary Data 2'

1884 29

&[num]

Anteholosticha_marimonilata 00120020010100101101011010

Antetintinnidium_mucicola 110*00*110*11****0*0*00011

Aspidisca_leptaspis 0011032000101010 1120*00010

Blepharisma_americanum 0010020001000*0*00*0*00000

Blepharisma_hyalinum 0010020001000*0*00*0*00000

Caryotricha_minuta 00110420011 1?11?01211100?0

Caryotricha_rariseta 000*03200111?11?01211100?0

Caryotricha_sinica 001103200111?11?01211100?0

Certesia_quadrinucleata 00100120011010101111000010

Diophrys_appendiculata 000*02200111010111 1001010

Discocephalus_ehrenbergi 000*11200101001011 11011010

Epiclintes_rarisetus 00?20020010100100101010010

Euplotes_aediculatus 00110220011010101111001010

Euplotidium_itoi 000*02200110001011 11000010

Gastrocirrhus_monilifer 100*02201110001011 10*00010

Holosticha_heterofoissneri 00120020010100101101010010

Kiitricha_marina 00??04200111111101211 10000

Licnophora_lyngbycola 000?1?*001000*1?*??0*00112

Licnophora_macfarlandi 000?12*001000*10*??0*00112

Metaurostylopsis_antarctica 00120020010100101101011010

Oxytricha_granulifera 00120020010100101101011010

Phacodinium_metchnikoffi 0010031001100*0000*0**0010

P_metchnikoffi_Wuhan 0010031001100*0000*0*00010

Prodiscocephalus_borrori 000*11200101001011 11011010

Propecingulum_fistoleramalliei 101200*010*11****0*0*00011

Pseudoamphisiella_alveolata 000*?1200101001011 01010010

Stenosemella_pacifica 11??00*110*11****0*0*00011

Uronychia_setigera 000*0220011010101111001010

UFRJ_DG_762_Pb   001102200110?0110121?1?010

&[DNA]

Anteholosticha_marimonilata ATG-
ATATACAGTGAAACTGCGAATGGCTCATTAAAACAGTTATAGTTTATTTGATAATCAAA-T-------------
TTACATGGATAACCGTGGTAATTCTAGAGCTAATACATGCTGTTTAGCCTGACTTTT--
TGAAGGGCTGTATTTATTAGATA-ACAAACCAATATTCC--TCG-TGTCTATTGTGACGACTCATAATAAC--
TGATCGAA-TCGCATGGACTCTGTCCGCGATACATCATTCAAGTTTCTGC--CCCATCA-
GCTTTCGATGGTAGTGTATTGGACTACCATGGCTTTAACGGG-
TAACGGAGGATTAGGGTTCGATTCCGGAGAGGGAGCCTGAGAAACGGCTACCACATCTAAGGAAGGC
AGCAGGCGCGTAAATTACCCAATCCTAATTCAGGGAGGTAGTGACAAGAAATAACGGACTGAGGCCAA
C--GCTTCAGGATTGCAAT-GAGTAGAATCTA-
AACCCCTTTGCAAGTATCAATTGGAGGGCAAGTCTGGTGCCAGCAGCCGCGGTAATTCCAGCTCCAATA
GCATATATTTAAGTTGTTGCAGTTA--AAAAGCTCGTAGTTGGATTTCTGG-----------GAGGATTC-
CAATG-TCCGC--TTG-TTGCGTGTTG-CAGCGGAGTCCTTCCATCCTTCTGTTAACT-TTTC-
TGGTATTTATTT----ACTGG--TTGAGGGCTCAGATATTTTACC--------------------------------------
TTGAGAAAATTAGAGTGTTTCAGGCAGGCTTGCGCC-
CGAATACATTAGCATGGAAGAATAGAATAGGACTT-----------------TGGTCTCCTTTGTTGGT-
TTGAGGGACTAGAG-TAATGATTAAT-AGGGACAGTT-----------
GGGGGCATTAGTATTTAATTGTCAGAGGTGAAATTCTCGGATTTGTTAAAGACTAACTTATGCGAAAGC
A---TTTGCCAAGGATGTTTTCATTAATCA-
AGAACGAAAGTTAGGGGATCAAAGACGATCAGATACCGTCGTAGTCTTAACCATAAACTATGCCGACT
AGGGATCGGAGG-CGCGT-TTTA--TCCGCCTTCGGAACC-TTATGAGAAATCAAAGTC-
TTTGGGTTCTGGGGGGAGTATGGTCGCAAGGCTGAAACTTAAAGGAATTGACGGAAGGGCACCACCA
GGAGTGGAGCTTGCGGCTCAATTTGACTCAACACGGGAAAACTTACCAGGTCCAGACATTGGAAGGAT
TGACAGATTGATAGCTCTTTCTTGATTCTATGGG-----
TGGTGGTGCATGGCCGTTCTTAGTTGGTGGAGTGATTTGTCTGGTTAATTCCGTT-
AACGAACGAGACCTTAGCCTACTAACTAGTCGT-TTCGATCTTTATCGT---
TCACGACTTCTTAGAGGGACTTTGTG-
GTTTCAAACACAAGGAAGTTTGAGGCAATAACAGGTCTGTGATGCCCTTAGATGTCCTGGGCCGCACG
CGTGCTACACTGACGCATACAGCGA-------GTACTT---------CCCAGCTCCGAGAGG-CAGCTGG-
TAATCA--GCAATATGCGTC-GTGCTGGGGATAGA-
TCTTTGGAATTATAGATCTTGAACGAGGAATTCCTAGTAAGCGCAGGTCAACAGCCTGCGCTGATTAAG
TCCCTGCCCTTTGTACACACCGCCCGTCGCTCCTACCGATTTCGAGTGGTCCGGTGAACCT-
TTTGGACCGCGT-TGAGT-CTCGTGCTCG--
ATGTGGGAAATCAAGTAAACCATATCACTTAGAGGAAGGAGAAGTCGTAACAAGGTT

Antetintinnidium_mucicola ATG-
CTATACAATGAAACTGCGAATGGCTCATTAAAACAGTTATAGTTTATTTGGTAATCGAA-T-------------
TTACATGGATAACCGTGGTAATTCTAGAGCTAATACATGCTGATAAGCCCGACTTCT-
CGGAAGGGCCGTATTTATTAGATA-ACAGACCAATATTCCC-TGG-
TGTCTATTGTGGTGACTCATAGTAAC--TGATCGGA-TCGCACGGGCGTG-
CCCGCGACAAACCATTCAAGTTTCTGC--CCCATCA-
GCTTTCGATGGTAGTGTATTGGACTACCATGGCTTTTACGGG-
TAACGGAGGATTAGGGTTCGATTCCGGAGAGGGAGCCTGAGAAACGGCTACCACATCTAAGGAAGGC

AGCAGGCGCGTAAATTACCCAATCCTGACTCAGGGAGGTAGTGACAAGAAATAACGGGCCGGGTATC
A---TATCCGGGACTGAAAT-GAGATGAATTTA-
CACCCCTTATTGAGGATCAATTGGAGGGCAAGTCTGGTGCCAGCAGCCGCGGTAATTCCAGCTCCAATA
GCGTATATTAAAGTTGTTGCAGTTA--AAAAGCTCGTAGTTGGATTTCTGG-----------TTGGGCGT-
CGATG-CCGGC--TCTGATGCCTG-TG-CAGAGATACCCTGCCATCCTTCTGTTAACC-
TGCGTTGGCATTCACTT----GCCGG--GTAGGGGCTCAGATATTTTACC--------------------------------------
TTGAGAAAATTAGAGTGTTTCAGGCAGGCTAGCGTC-
GGAATACATTAGCATGGAATAATGGAATAGGACTT------------------ATACCTCTTCTGTTGGT-
CTGAGGGGTGCAAG-TAATGATTAAT-AGGGATAGTT------------
GGGGGCATTAGTACTTAACTGTCAGAGGTGAAATTCTCGGATTTGTTAAAGACTAACTTATGCGAAAGC
A---TTTGCCAAGGATGTTTTCATTAATCA-
AGAACGAAAGTTAGGGGATCAAAGACGATCAGATACCGTCCTAGTCTTAACCATAAACTATGCCGACTC
GGGATCGGGAG-CGAGA-GTTA--TCCGCTTTCGGCACC-GTATGAGAAATCAAAGTC-
TTTGGGTTCTGGGGGGAGTATGGTCGCAAGGCTGAAACTTAAAGGAATTGACGGAAGGGCACCACCA
GGCGTGGAGCTTGCGGCTCAATTTGACTCAACACGGGGAAACTTACCAGGTCCAGACATAGTGAGGAT
TGACAGATTGATAGCTCTTTCTTGATTCTATGGG-----
TGGTGGTGCATGGCCGTTCTTAGTTGGTGGAGTGATTTGTCTGGTTAATTCCGTT-
AACGAACGAGACCTTAGCCTACTAAATAGTTCC-GATAA-TTCCATTAG---
CGGCAACTTCTTAGAGGGACTTTGT--AGT--
AAATACAAGGAAGTTTGAGGCAATAACAGGTCTGTGATGCCCTTAGATGTCCTGGGCCGCACGCGTGC
TACACTGACGCATACAGCGA-------GT--TA---------ACCGGCTCCGAGAGG-CAGCTGG-TAATCA--
GCAATATGCGTC-GTGATGGGGATAGA-
TCTCTGGAATTTTTGATCTTGAACGAGGAATGCCTAGTAAGCGCAGGTCATCAACCTGCGTTGATTAAG
TCCCTGCCCTTTGTACACACCGCCCGTCGCTCCTACCGATTTCGAGTGGTCCGGTGAACCG-
TTTGGACCGGGA-TCAGT-CTTGTGCTGA--
GCCTGGGAAGTTCAGTAAACCACATCACTTAGAGGAAGGAGAAGTCGTAACAAGGTT

Aspidisca_leptaspis ATC---------
TGAAACTGCGAATGGCCCATTAAAACAGTTATAGTTTACATGATACTGGGCT---------------
TTACATGGATATCCGTGGTAAA-CCAGAGCTAATACATGC-GTTACGTCCGACTTTT-
TGGAAGGACAGTATTTATTAGATT----AACCTATGCT----TTG--------
CATGGTGACTCACAATAACTTTGATCGAA-TCG--
TTGGCCGTGCCGACGATGAATCATTCAAGTTTCTGC--CCCTTCA-GCTA--
GATGGTAGTGTATTGGACTACCATGGCTTTTACGGG-
TAACGGGGGATTAGGGTTCGACACCGGAGAGGGAGCCTGAGAAACGGCTACCACATCTACGGAAGGC
AGCAGGCGCGTAAATTACCCAATCCTAATTCAGGGAGGTAGTGACAAGAAATAACAG-
ACCGGGTTTAC-----CCGGGTTTGCAAT-GGGCCACATTTA-
CACTAAGAAGCTAGGAACCATTGGAGGGCAAGTCTGGTGCCAGCAGCCGCGGTAATTCCAGCTCCAAT
AGCGTGTGTTAAAATTGTTGCAGTT---AAAAGCTCGTAGTTGGACTTCTGA-----------AGG------
CAACAGGCGTC--TTGTACGCTTG-TG-----------ACTTCATCCTTCTGTTAACA-GTTCTTGGCCTTCATTG---
-GCTGG--TTCTGGGCTCAGATTCTTTACC--------------------------------------
ATGAGGAAAATAGATTGTTTCAGGCAGGCTCGCGCC-GGAATACGTTAGCATGGAATA-TTT--
TACGACTT------------------CGTGTCTCTCATTTGG--TTGGGTG--CGAAG-TAATGGTCAC--
AGGGACAGTT-------------
GGGGCATTTGAATTCCATTGTCAGAGGTGAAATTCTTTGATTTGTGGAAGTCTAACTAATGCGAAAGCA
---TTTGCCAAGAATGTTTTCATTAATCA-
AGAACGAAAGTTAGGGGATCAAAGATGATCAGATACCGTCCTAGTCTTAACCATAATCTATGCCGACTA
GGGATCGGAGG-CGCGC-GCTT--TCCGCCTTCGGCACC-TTATGAGAAATCAAAGTC-TTCG-

GTTCTGGGGGAAGTATGGTCGCAAGGCTGAAACTTAAAGGAATTGACGGAAGGGCACCACCAGGAGT
GGAGCCTGCGGCTTAATTTGACTCAACACGGGAAAAATCACCAGGTCCAGACATAGGAAGGATTGACA
GATTGATAGCTCTTTCTTGATTCTATGGG-----
TGGTGGTGCATGGCCGTTCTTAGTTGGTGGAGCGATTTGTCTGGTTAATTCCGAT-
AACGAACGAGACCTTAGCCTGCTAACTAGTTGTCGACCTTCCGAGGCTG---
TATCAACTTCTTAGAGGGACTTTGTG-CG---
AATCACAAGGAAGTTTGAGGCAATAACAGGTCTGTGATGCCCTTAGATGTCCTGGGCCGCACGCGTGC
TACACTGATACGGACAAAAA-------GT--CC---------TCCTGAGCCGAAAGG-CCCCGGG-TAAACT--
GCAATCCGTATC-GTGATGGGGATAGA-
TCCTTGCAATTATGGATCTTGAACGAGGAATTCCTAGTAAGTGCAAGTCATCATCTTGCGCTGATTAAGT
CCCTGCCCTTTGTACACACCGCCCGTCGCTTCTACCGA--CAGAGTGGTCAGGTAAGCCT-
TTTGGACCGG---------CTGGTCTCC---
AGCCGAGAAATCAAGCAAGCCCAATCACTTGGAGGAAGAAGAAGTCGTAACAAGGTT

Blepharisma_americanum
GCAATTATACGGCGAGACTGCGAATGGCTCATTAAAACAGTTATAGTTTATTTGCTA---GACGT----------
----TTATATGGATAACCGTAGTAATTCTAGAGCTAATACATGCTGGTTACGCTG-----------
TGCGTAGTATTTATTAGAT--AAAACCCAACG------GGGGCGACCCTGTGGAGGAATCATAATAAC--
TTAGCGAACTCGA------------CGTAGTGAAGCATTCAAGTTTCTGC--CCTATCA-
GCTTTCGATGGTAGTGTATTGGACTACCATGGCGATGACGGG-
TGACGGAGAATTAGGGTTCGATTCCGGAGAGGGAGCCTGAGAAATGGCTACCACATCTAAGGAAGGC
AGCAGGCGCGCAAATTACCCAATCCTAACTCAGGGAGGTAGTGACAAGAAATAACAACGCGGGGCTTT
---GTCTTGCGATTGGAAT-GAGTTAAGTGTA-AAAGCC-TTTCGAGGACCCACTGGAGGGCAAGTCT-
GTGCCAGCAGCCGCGGTAATTCCAGCTCCAGTAGCGTATATTAAAGTTGTTGCAGTTA--
AAAAGCTCGTAGTTGAAGCTCTGC-----------GGGGCGGGTCGCTGGGCCTCGGTCTGGTGACCT---------
---TCCTCGCATCCATCTGAGCAAC-GGCTCCGGCATTAACTT----GTCGC--
TGTCGTGATCAGGTACTTTACC---------------------------------------
TTGAGCAAATGAGAGTGTTCCAGGCAGGCTTGGGCC-
TGAATCGTCCAGCATGGAATAATAGAAGAGGACTG------------------
GGCTCCATTTATTGGTGTTATGGCGCTTAG--TAATGATTAAT-AGGGATAGTT------------
GGGGGCATTTGTATTTAATTGTCAGAGGTGAAATTCTATGATTTATTAAAGACAAACTTATGCGAAAGC
A---TTTGCCAAGGATGTTTTCATTAATCA-
AGAACGAAAGTTAAGGGATCAAAGACGATCAGATACCGTCGTAGTCTTAACCATAAACTATGCCGACT
AGAGATTGGAGG-TGCCATTAAAAGTACTCCTTCAGCATC-TTCCGAGAAATCAAAGTC-
TTTGGGTTCTGGGGGGAGTATGGTCGCAAGACTGAAACTTAAAGGAATTGACGGAAGGGCACCACCA
GGAGTGGCA-
TGGCGGCTTAATTTGACTCAACACGGGGAAACTTACCAGGTCCAGACATGGGAAGGATTGACAGATTG
ATAGCTCTTTCTTGATTCTATGGG-----
TGGTGGTGCATGGCCGTTCTTAGTTGGTGGAGTGATTTGTCTGGTTAATTCCGAT-
AACGAACGAGACCTTAACCTGCTAACTAGTCGT---CTCTTGCCAAAGA---
GTCGGACTTCTTAGAGGGACTTTGGG-GGC--
TACTCCAAGGAAGTTTAAGGCAATAACAGGTCTGTGANGCCCTTAGANG-
CCTGGGCCGCACGCGCGCTACACTGATGCAGGCAGTAA-------GCCCTC------------
CGCGGCAGAAAATGCCCCGG-TAACCT--TCAAACTGCATC-GTGATGGGGATTGA-
CTCTTGGAATTATTGGTCATCAACGAGGAATTCCTAGTAAACGCAAGTCATCAACTTGCATTGACTACGT
CCCTGCCCTTTGTACACACCGCCCGTCGCTCCTACCGATTTCGAGTGATGAGGTGAATCC-
TCCGGACTGCACAC-----CTCGT-------
GTGTGGGAAGTTGAGTAAACCTTATCACTTAGAGGAAGGAGAAGTCGTAACAAGGTA

Blepharisma_hyalinum
GCAATTATACGGCGAGACTGCGAATGGCTCATTAAAACAGTTATAGTTTATTTGCTA---AACGT-----------
---TTATATGGATAACCGTAGTAATTCTAGAGCTAATACATGCTGGTTACGCTG-----------
TGCGTAGTATTTATTAGAT--AAAACCCAACG------GGGGCGACCCTGTGGAGGAATCATAATAAC--
TTAGCGAACTCGA------------CGTAGTGAAGCATTCAAGTTTCTGC--CCTATCA-
GCTTTCGATGGTAGTGTATTGGACTACCATGGCGATGACGGG-
TGACGGAGAATTAGGGTTCGATTCCGGAGAGGGAGCCTGAGAAATGGCTACCACATCTAAGGAAGGC
AGCAGGCGCGCAAATTACCCAATCCTAACTCAGGGAGGTAGTGACAAGAAATAACAACGCGGGGCTTT
---GTCTTGCGATTGGAAT-GAGTTAAGTGTA-
AAAGCCTTTTCGAGGACCCACTGGAGGGCAAGTCTGGTGCCAGCAGCCGCGGTAATTCCAGCTCCAGT
AGCGTATATTAAAGTTGTTGCAGTTA--AAAAGCTCGTAGTTGAAGCTCTGC-----------
GGGGCGGATAGCTGGGCTTCGGTCTGGT------TG------TCTTCCTCGCATCCACCTGAGCAGC-
AGCTCCGGCATTAACTT----GTCGC--AGTTGTGATCAGGTACTTTACC--------------------------------------
TTGAGCAAATGAGAGTGTTCCAGGCAGGCTCGGGCC-
TGAATCGTCCAGCATGGAATAATAGAAGAGGACTG-------------------
GGCTCCATTTATTGGTGTTATGGCGCTTAG--TAATGATTAAT-AGGGATAGTT------------
GGGGGCATTTGTATTTAATTGTCAGAGGTGAAATTCTATGATTTATTAAAGACAAACTTATGCGAAAGC
A---TTTGCCAAGGATGTTTTCATTAATCA-
AGAACGAAAGTTAAGGGATCAAAGACGATCAGATACCGTCGTAGTCTTAACCATAAACTATGCCGACT
AGAGATTGGAGG-TGCAATTTCA-GTACTCCTTCAGCATC-TTCCGAGAAATCAAAGTC-
TTTGGGTTCTGGGGGGAGTATGGTCGCAAGACTGAAACTTAAAGGAATTGACGGAAGGGCACCACCA
GGAGTGGAGCCTGCGGCTTAATTTGACTCAACACGGGGAAACTTACCAGGTCCAGACATGGGAAGGA
TTGACAGATTGATAGCTCTTTCTTGATTCTATGGG-----
TGGTGGTGCATGGCCGTTCTTAGTTGGTGGAGTGATTTGTCTGGTTAATTCCGAT-
AACGAACGAGACCTTAACCTGCTAACTAGTTGT---CTCTCGTCAGAGA---
GTCGAACTTCTTAGAGGGACTTTGGG-GGC--
TACTCCAAGGAAGTTTAAGGCAATAACAGGTCTGTGATGCCCTTAGATGTCCTGGGCCGCACGCGCGCT
ACACTGATGCAGGCAGTAA-------GCCCTC------------CGCGGCAGAAAT-GCCCCGG-TAACCT--
TCAAACTGCATC-GTGATGGGGATTGA-
CTCTTGGAATTATTGGTCATCAACGAGGAATTCCTAGTAAACGCAAGTCATCAACTTGCATTGACTACGT
CCCTGCCCTTTGTACACACCGCCCGTCGCTCCTACCGATTTCGAGTGATGAGGTGAATCC-
TCCGGACCGCACAC-----CTCGT-------
GTGTGGGAAGTTGAGTAAACCTTATCACTTAGAGGAAGGAGAAGTCGTAA???????

Caryotricha_minuta ATG-
TTATACAGTGAAACTGCGAATGGCTCATTAAAACAGTTATAGTTTATTTGATGTTGGAAGAT-------------
TTACATGGATAACCGTGGTAATTCTAGGGCTAATACATGCTTAAATGCCTTACTTTCGCGGAATGGCTGT
ATTTATTAGAT--AAAAACCTCTACCTT------CGGGTCTTGTGGTGAGTCATAATAAC--TGATCGAA-
TCGC-CGGGCACTGCCTGCGATAAATCATTCAAGTTTCTGC--CCCATCA-G-
TTTCGATGGTAGTGTATTGGACTACCATGGCTTTAACGGG-
TAACGGAGAATTAGGGTTCGATTCCGGAGAGGGAGCCTGAGAAACGGCTACCACATCTATGGATGGC
AGCAGGCGCGTAAATTACCCAATCCTGATTCAGGGAGGTAGTGACAAGAAATAACGATACAGACCTTT
G--GTTTTGTAATTGTAAT-GGGCCTAATTTA-
TACCTCTTAGTGAGTAGCAATTGGAGGGCAAGTCTGGTGCCAGCAGCCGCGGTAATTCTAGCTCCAATA
GCGTATATTAAAGTTGTTGCAGTTA--AAAAGCTCGTAGTTGGATTTCTGA-------------
GAGCGTTTCTTTCTCATTCCACTGGTTTGCA-CG-ATTGACAACTCTCTCATCCTTCTGTTAACC-
TGTTCAAGCATTCATTT----GCTTT--TCAGGGGCTCAGTTATTTTACC--------------------------------------
TTGAGAAGATTAGAGTGTTTCAAGCAAGTTTGTGCC-

TGAATACATTAGCGTGGAATAATAGAATAGGACAT------------------CCGTCTCG-TTGTTGGT---
ATGAGTTGGAAG-TAATGATTAAT-AGGGACAGTT------------GGGGGCATTAGTATTTAATTGTCAGA-
GTGAAATTCTTTGATTTGTTAAAGACTAACTTATGCGAAAGCA---TTTGCCAAG-ATG-TTTCATTAATCA-
AG-ACGAAAGTTA-
GGGATCAAAGACGATCAGATACCGTCCTAGTCTTAACCATAAACTATACCGACTAGGGATTGGAGGAA
GAAATTATA--TTCTCCTTCAGCACC-TTACGAGAAATCAAAGTC-
TTTGGGTTCTGGGGGGAGTATGGTCGCAAGGCTGAAACTTAAAGGAATTGACGGAAGGGCACCACCA
GGAGTGGAGCTTGCGGCTTAATTTGACTCAACACGGGAAAACTTACCAGGTCCAGACATGAGAAGGAT
TGACAGTTTGATAGCGCTTTCTTGATTGTATGGG-----
TGGTGGTGCATGGCCGTTCTTAGTTGGTGGAGTGATTTGTCTGGTTAATTCCGTT-
AACGAACGAGACCTTAACCTGCTAACTAGTTCTACCACTTTGTGGGAAG------
ACACTTCTTAGAGGGACAGTTGACATAAAAAGTCAAAGGAGGTTTGAGGCAATAACAGGTCTGTGATG
CCCTTAGATGTCCTGGGCCGCACGCGTGCTACACTGATACATACAACAA-------GTATTT----------
CTTGCCCCGAAAGG-TGGCGAG-TAATCT--CTAATATGTATC-GTGATGGGGATAGA-
TCTTTGTAATTATGGATCTTGAACGAGGAATTCCTAGTAAGTGCAGGTCATTAGCCTGTACTGATTAAGT
CCCTGCCCTTTGTACACACCGCCCGTCGCTCCTACCGA--TCGAGTGATCCGGTGAATCT-
TTTGGACCGTAA-----------------------
GGGAAATCAAGTAAACCTTGTCACTTAGAGGAAGGAGAAGTCGTAACAAGGTT

Caryotricha_rariseta ATG-
TTATACAGTAAAACTGCGAATGGCTCATTAAAACAGTTATAGTTTATTTGATGTTGGAAGA--------------
TTACATGGATAACCGTGGTAATTCTAGGGCTAATACATGCTTAAATGCCTTACTTTG-
CGGAATGGCTGTATTTATTAGAT--ACAAACCAATACCTTC-----GGGTCATTGTGGTGAGTCATAATAAC-
-TGATCGAA-TCGC-CGGGCCTTGCCTGCGATAAATCATTCAAGTTTCTGC--CCCATCA-G-
TTTCGATGGTAGTGTATTGGACTACCATGGCTTTAACGGG-
TAACGGAGAATTAGGGTTCGATTCCGGAGAGGGAGCCTGAGAAACGGCTACCACATCTATGGATGGC
AGCAGGCGCGTAAATTACCCAATCCTGATTCAGGGAGGTAGTGACAAGAAATAACTATACAGACCTTC
G--GTTTTGTAATTGTAAT-GGACCTAATTTA-
TACCTCTTAGTGAGCAGCCATTGGAGGGCAAGTCTGGTGCCAGCAGCCGCGGTAATTCCAGCTCCAAT
AGCGTATATTAAAGTTGTTGCAGTTA--AAAAGCTCGTAGTTGGATTTCTGA-----------GAGAGTTG-
CTTTC-TCATTCCCATGGTTTGCA-CG-ATTGCGGACTCTCTCATCCTTCTGTTAACC-
ATTGCAAGCATTGATTT----GCTTC--TTTGGGGCTCAGTTATTTTACC---------------------------------------
TTGAGAAAATTAGAGTGTTTCAAGCAAGTTTGTGCC-
TGAATACATTAGCATGGAATAATTGAATAGGACCT------------------CCGCCTCG-TTGTTGGT-
ATCCGAGTAGGAAG-TAATGATTAAT-AGGGACAGTT------------
GGGGGCATTAGTATTTAATTGTCAGAGGTGAAATTCTTTGATTTGTTAAAGACTAACTTATGCGAAAGC
A---TTTGCCAAGGATGTTTTCATTAATCA-
AGAACGAAAGTTAGGGGATCAAAGACGATCAGATACCGTCCTAGTCTTAACCATAAACTATACCGACTA
GGGATTGGAGGACGAAACTACA--TTCTCCTTCAGCACC-TTACGAGAAATCAAAGTC-
TTTGGGTTCTGGGGGGAGTATGGTCGCAAGGCTGAAACTTAAAGGAATTGACGGAAGGGCACCACCA
GGAGTGGAGCTTGCGGCTTAATTTGACTCAACACGGGAAAACTTACCAGGTCCAGACATTAGAAGGAT
TGACAGTTTGATAGCGCTTTCTTGATTGAATGGG-----
TGGTGGTGCATGGCCGTTCTTAGTTGGTGGAGTGATTTGTCTGGTTAATTCCGTT-
AACGAACGAGACCTTAACCTGCTAACTAGTGCT-GACATTCGTGGACAG------
ACACTTCTTAGAGGGACAGTTGACATAAAAAGTCAAAGGAGGTTTGAGGCAATAACAGGTCTGTGATG
CCCTTAGATGTCCTGGGCCGCACGCGTGCTACACTGATGCATACAACAA-------GCACTT----------
CTTGCCCCGATAGG-TGGCGAG-TAATCT--CTAATATGCATC-GTGATGGGGATAGA-
TCTTTGTAATTATGGATCTTGAACGAGGAATTCCTAGTAAGTGCAGGTCATTAGCCTGTACTGATTAAGT

CCCTGCCCTTTGTACACACCGCCCGTCGCTCCTACCGA--TCGAGTGATCCGGTGAATCT-
TTTGGACCGTAA----------------------
GGGAAATCAAGTAAACCTTGTCACTTAGAGGAAGGAAAAGTCGTAACAAGGTT

Caryotricha_sinica ATG-
TTATACAGTAAAACTGCGAATGGCTCATTAAAACAGTTATAGTTTATTTGATGTTGGAAGA-------------
TTACATGGATAACCGTGGTAATTCTAGGGCTAATACATGCTTAAATGCCTTACTTTG-
CGGAATGGCTGTATTTATTAGAT--AAAAACCAATACCTTC-----GGGTAATTGTGGTGAGTCATAATAAC-
-TGATCGAA-TCGC-CGGGCATTGCCTGCGATAAATCATTCAAGTTTCTGC--CCCATCA-G-
TTTCGATGGTAGTGTATTGGACTACCATGGCTTTAACGGG-
TAACGGAGAATTAGGGTTCGATTCCGGAGAGGGAGCCTGAGAAACGGCTACCACATCTATGGATGGC
AGCAGGCGCGTAAATTACCCAATCCTGATTCAGGGAGGTAGTGACAAGAAATAACGATACAGACCTTC
G--GTTTTGTAATTGTAAT-GGACCTAATTTA-
TACCTCTTAGTGAGAAGCAATTGGAGGGCAAGTCTGGTGCCAGCAGCCGCGGTAATTCCAGCTCCAAT
AGCGTATATTAAAGTTGTTGCAGTTA--AAAAGCTCGTAGTTGGATTTCTGA-----------AAGAGTAA-
CTTTC-TCATTCCCATGGTTTGCA-CG-ATTGTGGACTCTTTCATCCTTCTGTTAACC--
ATCACGGCATTGATTT----GTCGC--GTTGGGGCTCAGTTATTTTACC--------------------------------------
TTGAGAAAATTAGAGTGTTTCAAGCAAGTTTGTGCC-
TGAATACATTAGCATGGAATAATAGAATAGGACCT------------------CCGCCTCG-TTATTGGT-
ATCCGAGTTGGAAG-TAATGATTAAT-AGGGACAGTT------------
GGGGGCATTAGTATTTAATTGTCAGAGGTGAAATTCTTTGATTTGTTAAAGACTAACTTATGCGAAAGC
A---TTTGCCAAGGATGTTTTCATTAATCA-
AGAACGAAAGTTAGGGGATCAAAGACGATCAGATACCGTCCTAGTCTTAACCATAAACTATACCGACTA
GGGATTGGAGGACGAAACTACA--TTCTCCTTCAGCACC-TTACGAGAAATCAAAGTC-
TTTGGGTTCTGGGGGGAGTATGGTCGCAAGGCTGAAACTTAAAGGAATTGACGGAAGGGCACCACCA
GGAGTGGAGCTTGCGGCTTAATTTGACTCAACACGGGAAAACTTACCAGGTCCAGACATTAGAAGGAT
TGACAGTTTGATAGCGCTTTCTTGATTGCATGGG-----
TGGTGGTGCATGGCCGTTCTTAGTTGGTGGAGTGATTTGTCTGGTTAATTCCGTT-
AACGAACGAGACCTTAACCTGCTAACTAGTGCT-TCCATTCGTGGTTAG------
ACACTTCTTAGAGGGACAGTTGACATAAAAAGTCAAAGGAGGTTTGAGGCAATAACAGGTCTGTGATG
CCCTTAGATGTCCTGGGCCGCACGCGTGCTACACTGATGCATACAACAA-------GCACCT----------
CTTGCCCCGATAGG-TGGCGAG-TAATCT--CTAATATGCATC-GTGATGGGGATAGA-
TCTTTGTAATTATGGATCTTGAACGAGGAATTCCTAGTAAGTACAGGTCATTAGCCTGTGCTGATTAAGT
CCCTGCCCTTTGTACACACCGCCCGTCGCTCCTACCGA--TCGAGTGATCCGGTGAATCT-
TTTGGACCGTAA----------------------
GGGAAATCAAGTAAACCTTGTCACTTAGAGGAAGGAGAAGTCGTAACAAGGTT

Certesia_quadrinucleata
GCTTTTATATGGCGAAACTGCGAATGGCTCATTAAAACAGTTATCGTTTACTTGATATTTGAATT----------
----TTATATGGATAACCGTAGTAATTCTAGAGCTAATACATGC-GTTGAGGGTGAAAT---
TAGTAGCCCAGTATTTATTAGATATTCAGACCAATATTCCT-TCG-
GGTCTATTGTGATGATTCATAGTAAC--TGATCGGA-TCGC-
TGGGTTCTGCCCGCGACAAGTCATTCAAGTTTCTGC--CCCATCA-GCTT--
GTTGGTAGTGTATTGGACTACCAAGGCTTTCACGGG-
TAACGGAGGATTAGGGTTCGATTCCGGAGAGGGAGCCTGAGAAACGGCTACCACTTCTACGGAAGGC
AGCAGGCGCGTAAATTACCCAATCCTGATTTAGGGAGGTAGTGACAAGAAATAATAGTACCGGGTTCT-
----CCCGGGTTTATAAT-GGGACTAATCTA-
CACAACTTATCGAGGAACCATTGGAGGGCAAGTCTGGTGCCAGCAGCCGCGGTAACTCCAGCTCCAAT
AGCGTATATTAAAGTTGTTGCAGTTA--AAAAGCTCGTAGTTGGATTTCTGG-----------

AGGAGGGCTCGATGGGCAGC-TACTGTTGCCTG-TG-CG--AGCTCTTCTTCATCCTCCTGTCAACT-
TCGTTCGAGATTCGTTT----CTCGG--CGTTGGGCCCAGGAATTTTACC-------------------------------------
TTGAGGAAATTAGAGTGTTTCAGGCAGGCAATCGCC-
CGAATACATTAGCATGGAATAATGGAAGAGGACTG-----------------TTTCTCCTTTTGTTGGT-
TTGAGGAGCACAG--TAATGATTAAT-AGGGATAGTT------------
GGGGGCATTAGTATTCTGCAGTCAGAGGTGAAATTCTTTGATCTGTGGAAGACTGACTTATGCGAAAG
CT---TTTGCCAAGAATGTTTTCATTAATCA-
AGAACGAAAGTTAGGGGATCAAAGACGATCAGATACCGTCCTAGTCTTAACCATAAACTATGCCGACT
AGGGATCGGGGG-CGTGC-GTTT--TCCGCTCTCGGCACC-TTATGAGAAATCAAAGTC-
TTTGGGTTCTGGGGGGAGTATGGTCGCAAGGCTGAAACTTAAAGGAATTGACGGAAGGGCACCACCA
GGAGTGGAGCTTGCGGCTTAATTTGACTCAACACGGGAAAACTTACCAGGTCCAGACATAGGAAGGAT
TGACAGATTGATAGCTCTTTCTTGATTCTATGGG-----
TGGTGGTGCATGGCCGTTCTTAGTTGGTGGAGTGATTTGTCTGGTTAATTCCGTT-
AACGAACGAGACCTTAGCCTGCTAAATAGTCAC--ACGACCCTGGGTCG---
TGGTGTCTTCTTAGAGGGACTTTGTG-CGC--
AATCACAAGGAAGTTTAAGGCAATAACAGGTCTGTGATGCCCTTAGATGTCCTGGGCCGCACGCGTGC
TACACTGACACGTGCAATAA-------GTGCTG-----------CGACCTCGTGAGG--GGCCGC-TAATCT--
ATAATACGTGTC-GTGCTGGGGATAGA-
TCTTTGGAATTATGGATCTTGAACTAGGAATTCCTAGTATGTGCAAGTCATTAGCTTGCGCAGATTACGT
CCCTGCCCTTTGTACACACCGCCCGTCGCTGTTACCGATTTCGAGTGGTCCGGTGAATAG-
TTAGGATGAGGC-------TTCG--------
GCCCCAGAATTTCCGTAAACCTTATCACTTAGAGGAAGCAAAAGTCGTAACAAGGTT

Diophrys_appendiculata
GCGAATATACAGTGAAACTGCGAATGGCTCATTAAAACAGTTATAGTTTATTTGATAATGGAATT--------
------TTATATGGATAACCGTAGTAATTCTAGAGCTAATACATGCTGTCAAGCCCAACTTTT-
TGGACGGGCTGTGTTTATTAGAT--ACAAACCAATATTCC--CTA-GGTCTATTGT-ATGATTAATAATAAC-
-TGATCGAA-TCGCATGGGTTTT-CCCGCGATAAATCATTCAAGTTTCTGC--CCCATCA-GCTT--
GATGGTAGTGTATTGGACTACCATGGCGTTCACGGG-
TAACGGAGGATTAGGGTTCGATTCCGGAGAGGGAGCCTGAGAAACGGCTACCACATCTAAGGAAGGC
AGCAGGCGCGTAAATTACCCAATACTGACTCAGTGAGGTAGTGACAAGAAATAACAGACCGGAGCTTT
T--GCACCGGGATTGCAAT-GAGAACAATCTA-
AACCCCTTAACGAGGATCCATTGGAGGGCAAGTCTGGTGCCAGCAGCCGCGGTAATTCCAGCTCCAAT
AGCGTATATTAAAGTTGTTGCAGTTA--AAAAGCTCGTAGTTGGATTTCTGG-----------ATGGATGC-
CGATG-CCCGT--CTG-CGACGTG-TG-CAAAGGTGCCCGTTCATCCTTCTGTTAAGG-
TTTCTTAGTATTTATTT----ACTGG--TTTCCGGCTCAGATATTTTACC-------------------------------------
TTGAGTAAATTAGAGTGTTTCAGGCAGGCTTGGTTC-
TGAATACATTAGCATGGAATAATAGAATAGGATTC-----------------CAG-ATCCTTTGTTGGT-
TTCCGGATTTGGAG-TAATGATTAAT-AGGGATAGTT------------
GGGGGCATTAGTATTTAATTGTCAGAGGTAAAATTCTTGGATTAATTAAAGACTAACTTATGCGAAAGC
A---TTTGCCAAGGATGTTTTCATTAATCA-
AGAACGAAAGTTAGGGGATCGAAGACGATCAGATACCGTCGTAGTCTTAACCATAAACTATGCCGACT
AGGGATCGGAGG-CGGAT---TT--TACGCTTTCGGAACC-TTATGAGAAATCAAAGTC-
TTTGGGTTCTGGGGGGAGTATGGTCGCAAGGCTGAAACTTAAAGGAATTGACGGAAGGGCACCACCA
GGAGTGGAGCTTGCGGCTTAATTTGACTCAACGCGGGGAAACTTACCAGGTCCAGACATAGTGAGGAT
TGACAGATTGATAGCTCTTTCTTGATTCTATGGG-----
TGGTGGTGCATGGCCGTTCTTAGTTGGTGGAGTGATTTGTCTGGTTAATTCCGTT-
AACGAACGAGACCTTAGCCTGCTAAATAGTTGC-AGTCCTACTTAGGGC---

TGTAAACTTCTTAGAGGGACTTTGTG-CA---
AAACACAAGGAAGTTTGAGGCAATAACAGGTCTGTGATGCCCTTAGATGTCCTGGGCCGCACGCGTGC
TACACTGACACTTACAACGA-------GTA--T---------TCCTGTTCCGAAAGG-CTACAGG-TAATCT--
ATAATAAGTGTC-GTGATGGGGATAGA-
TCTTTGGAATTATAGATCTTGAACGAGGAATTCCTAGTAAGCGCAAGTCATCATCTTGCGCTGATTAAGT
CCCTGCCCTTTGTACACACCGCCCGTCGCTCCTACCGATTTCGAGTGATCCGGTGAACTA-
TTCGGACTGTG--------CTAGCAATA---
GCGTAGGAAGTCTCGTGAACCTTATCACTTAGAGGAAGGAGAAGTCGTAACAAGGTT

Discocephalus_ehrenbergi GCT-
TTATGTTGTGAAACTGCGAATGGCTCATTAAAACAGTTATAGTTTATTTGATATTCGAATT-------------
TTATATGGATAACCGTGGTAATTCTAGAGCTAATACATGC-TTCAAGGCCGACTTTT-
TGGAAGGTCTGTATTTATTAGATA-ACAAGCCAATATTCC--TCG-
TGTCTATTGTGATGATTCATAATAAC--TGATCGAA-TCGC------
ATTTTTTGCGATAAATCATTTAAGTTTCTGC--CCCATCA-
GCTTTCGATGGTAGTGTATTGGACTACCATGGCTTTTACGGG-
TAACGGAGGATTAGGGTTCGATTCCGGAGAGGGAGCCTGAGAAACGGCTACCACATCTAAGGAAGGC
AGCAGGCGCGTAAATTACCCAATCCCGATTCGGGGAGGTAGTGACAAGAAATAACGGACCGGAGCCT
CGT-GCACCGGGATTGCAAT-GATTACAATTTA-
AACCCCTTAATGAGGAACAATTGGAGGGCAAGTCTGGTGCCAGCAGCCGCGGTAATTCCAGCTCCAAT
AGCGTATATTAAAGTTGTTGCAGTTA--AAAAGCTCGTAGTTGGATTTCTGG-----------TCGAGTGC-
TGAGG-TCCGT-TAGAAATGCGTG-TG-CTAAAGCGCTCGGCCATCCTTCTGTTAACG-
GATCTTGGTATTAGTTT----ATCGG--TTCCGGGCTCAGATATTTTACC-------------------------------------
TTGAGAAAATTAGAGTGTTTCAGGCAGGCTCGCGCC-
CGAATACATTAGCATGGAATAATAGAATTGGAATT-----------------TTGTCTCTTTTGTTGGT-
TTGAGGGACTGAAT-TAATGATTAAT-AGGGATAGTT------------
GGGGGCATTAGTATTGAGCTGTCAGAGGTGAAATTCTTGGATTTGTTCAAGACTAACTTATGCGAAAGC
A---TTTGCCAAGGATGTTTTCATTAATCA-
AGAACGAAAGTTAGGGGATCAAAGACGATCAGATACCGTCCTAGTCTTAACCATAAACTATGCCGACT
AGAGATTGGAGG-GCAGC-TTCA--TCTGCCTTCAGCATC-TTATGAGAAATCAAAGTC-
TTTGGGTTCTGGGGGGAGTATGGTCGCAAGGCTGAAACTTAAAGGAATTGACGGAAGGGCACCACCA
GGAGTGGAGCTTGCGGCTCAATTTGACTCAACACGGGAAAACTTACCAGGTCCAGACATAGTAAGGAT
TGACAGATTGATAGCTCTTTCTTGATTCTATGGG-----
TGGTGGTGCATGGCCGTTCTTAGTTGGTGGAGTGATTTGTCTGGTTAATTCCGTT-
AACGAACGAGACCTTAGCCTACTAAATAGTTGC-ATTCTTTACGAAGGT---
TGCTAACTTCTTAGAGGGACTTTGTG-ATCT-
AAACACAAGGAAGTTTGAGGCAATAACAGGTCTGTGATGCCCTTAGATGTCCTGGGCTGCACGCGTGC
TACACTGACGTATACAACGA-------GTACCC---------TCCGGCTCCGCGAGG-CAGCCGG-TAATCT--
GCAATATACGTC-GTGATGGGGATAGA-
TCTTTGTAATTCTGGATCTTGAACGAGGAATTCCTAGTAAGCGCAGGTCATTAGCCTGCGCTGATTAAG
TCCCTGCCCTTTGTACACACCGCCCGTCGCTCCTACCGA--TCGAGTGTTCTGGTGAATCT-
TTTGGACCGTGGTGCCGGACTCGTGCCGTGCCCGTGGAAAATCATGTAAACTATAACACTTAGAGGAA
GGAGAAGTCGTAACAAGGTT

Epiclintes_rarisetus
?????????????GAAACTGCGAATGGCTCATTAAAACAGTTATAGTTTATTTGATA--GGGTTT-------------
TTACATGGATAACCGTGGTAATTCTAGAGCTAATACATGCT-TACAGCCCGACTTAC---GAAGGG-
TGTATTTATTAGATA-ACAAACCAATATTCC--TCG-TGTCTATTGTGGTGATTCATAATAAC--
TTGTCGGA-TCGCATGGACCTTGTCCGCGATAAGTCATTCAAGTTTCTGA--CCCATCA-

GCTTTCGATGGTAGTGTATTGGACTACCATGGCATTTACGGG-
TAACGGAGAATTAGGGTTCGATTCCGGAGAGGGAGCCTGAGAAACGGCTACCACATCTACGGAAGGC
AGCAGGCGCGTAAATTACCCAATCCTGACTCAGGGAGGTAGTGACAAGAAATAACGGACCGAGGCTCT
---GCTTCGGGATCGCAAT-GAGTACAATTTA-
AACCCCTTAACAATTAACAATTGGAGGGCAAGTCTGGTGCCAGCAGCCGCGGTAATTCCAGCTCCAATA
GCGTATACTAAAGTTGTTGCAGTTA--AAAAGCTCGTAGTTGGATTTCTGG----------CTTGTGCTT-
TTCGGACCCGC---CGTTGGTGTG-TG-TCCGAATGC-CGGCCATCTTTCTGCGGACG-
TTGTCTGACATTCATTT----GTTGGGTTTCTGCGAGCAGATATTTTACC-------------------------------------
TTGAGAAAATTAGAGTGTTT-AAGCAGGCTTA-GCC-
TGAATACATTAGCATGGAATAATAGAACAGGACTC-----------------CCGGTGTCATTTGTTGGT-
TTGAGGCAGTGGAG-TAATGATTAAT-AGGGATAGTT------------
GGGGGCATTAGTACTTAATTGTCAGAGGTGAAATTCTCGGATTTGTTAAAGACTAACTTATGCGAAAGC
A---TTTGCCAAGGATGTTTTCATTAATCA-
AGAACGAAAGTTAGGGGATCAAAGACGATCAGATACCGTCCTAGTCTTAACCATAAACTATGCCGACT
AGGGATCGGAGG-CGAGC--ATA--TCAGCCTTCGGCACCTTTATGAGAAATCAAAGTC-
TTTGGGTTCTGGGGGGAGTATGGTCGCAAGGCTGAAACTTAAAGGAATTGACGGAAGGGCACCACCA
GGCGTGGAGCTTGCGGCTCAATTTGACTCAACACGGGAAAACTTACCAGGTCCAGACATAGTTAGGAT
TGACAGATTGATAGCTCTTTCTTGATTCTATGGG-----
TGGTGGTGCATGGCCGTTCTTAGTTGGTGGAGTGATTTGTCTGGTTAATTCCGTT-
AACGAACGAGACCTTAGCCTACTAACTAGTTGA-TTCAATTTCCATTGA---
ATTTGACTTCTTAGAGGGACTATGTG-ACT--
AAACACAAGGAAGTTTGAGGCAATAACAGGTCTGTGATGCCCTTAGATGTCCTGGGCCGCACGCGTGC
TACACTGACGCATGCAGCGA-------GTACTT----------CTGGGTCCGAAAGGTTTCCCAG-TAATCA--
GCAATATGCGTC-GTGATGGGGATAGA-
CCTTTGGAATTCTTGGTCTTGAACGAGGAATGCCTAGTAAGCGCAAGTCATTAGCTTGCGTTGATTAAG
TCCCTGCCCTTTGTACACACCGCCCGTCGCTCCTACCGATTTCGAGTGATCCGGTGAACCT-
TTTGGACGGCGA-GGGTT-CCGGCCT-----
TTGTCGAAAATCAAGTAAACCACATCACTTAGAGGAAGGAGAAGTCGTAACAAGGTT

Euplotes_aediculatus AGG-
ATTTATAATGAAACTGCAAATGGCTCATTCAAACAGTTATAGTTTATTTGATATTCAAGCTAATATTCTTA
TTAGTTAAATGGATAACCGTAGTAATTCTAGAGCTAATACATGC-GTTACGGGGAACTTTA-
CGGGACCCCAGCGTTTATTAGAT--TTAAACCAATATTCCG-CAA-
GGTCTACTGAGATGATTCATGATAAC--TGATCGAA-TTGCTGGAACTCTA---
GCAATAAATCATGTAGGTTTCTGCTTCCCATCA-GCTT--
GATGGTAGTGTATTGGACAACCATGGCATTCACGGGCTATCGGGGGATTAGGGTTCGATTCCGGAGAG
GGAGCCTGATAAACGGCTACCACTTCTACGGAAGGCAGCAGGCGCGAAAATTATCCAATCCTGATTCA
GGGAGGTAGTGA-AAGAAATAATGAACTAGGATTT----ATCCTGGGATCACAAT-
GGGCTTGATTTGCAAACACTTAGCGAGGAACAATTGGAGGGCAAGTCTGGTGCCAGCAGCCGCGGTA
ATTCCAGCTCCAATAGAGTATATAAACGTTCCTGCAGTTATTCGATGCTCGTAGTTGGATTTCTGAGGTT
GAGACGGGAGGGTGG-CTATGGCCATC--------GCCCG--------ACTCTTTCCTCATCCACCTGTTTGCG-
AAGTCCGGGATTGATTT----CTCGG--CTTCGGGCGCAG-
TATCTTACCATTTCAATATTCGTAGTCTTCATCGATTATGTTGTTTCTTTGAGCAAATTATAGTGTTTCAG
GCAGGCGTGCGCC-GGAATACATTAGCATGGTATAAACGAATTGGACCG-
TGTGCCCGTAACTGGGCTTCTCCTTATTGTTGGT-TTGAAGGACACGGA--GATGGTTAAT-
AGGGACAGCGTTTATTTCACAAGGAGGCATTAGTATTTAATTTCCAGAGGTGAAATTCTTTGAAATATT
AAAGACTAACTTATGCGAAAGCATCGTTTGCCAATGATGTTTTCATTAATCATTGAACGAAAGTTAGGG
GATCAAAGACGATCAGATACCGTCCTAGTCTTAACCATAAACGTTGCCGACTAGGGATCGGAGGGCGT

GC-ACAA--TCCGCCTTCGGCACC-
TTACGAGAAATCAAAGTCTTTTGGGTTCTGTGGGTAGTATGGTCGCAAGGCTGAAACTTAAAGGAATT
GACGGAAGGGCACCACCAGGAGTGGAGCTTGCGGCTCAATTTGACTCAACACGGGAAATCTTACCAG
GTCCAGACATAGCGAGGATTGACAGATTGATAGCTCTTTCTTGATTCTATGGGTATTTTGGTGGTGCAT
GGCCGTTCTTAGTTCGTGGAGTGATTCGTCTGGTTAATTCCGTTAAACGAACGAGACCTCAGCCTGCTA
AATAGTTGCTCGCCTTTTCTATAAGACTTGACAACTTCTTAGAGGGACTTTGTG-TGC--
AACCACAAGGAAGTTTGAGGCAATAACAGGTCTGTGATGCCCTTAGATGTCCTGGGCCGCACGCGTGC
TACACTGATACGTACAACGAGGTATATGTACTTGTACATCAATGCTGCTCCGAAATA-
GACGAGCTTAATCTTATAAATACGTATC-GTGCTGCGGATAGA-
TCGTTGAAATTATGAATCTTGAAGGTGGAATTCCTAGTAAGCGCACGTCACTAACGTGCGTTGATTACG
TCCCTGCCCTTTGTACACACCGCCCGTCGCTCCTACCAATTTCGAGTGATCTGGTGAACCTCTTTGGACT
GT---CGAGCAATCGC------------
GAAATTAGAGTGAACCTGACTACTTAGAGGAAGGAGAAGTCGTAACAAGGTT

Euplotidium_itoi ATG-
TTATACAGTGAAACTGCGAATGGCTCATTAAAACAGTTATAGTTTATTTGATATTCAAG-T--------------
TTATATGGATAACCGTAGAAAAGCTAGAGCTAATACATGC-GTTACGCCTGACTTAT-
TGGAAGGGCAGTATTTATTAGATA-ACAATCCAATATTCCT-TCG-
GGTCTATTGTGATGATTCATAATAAC--TAATCGAA-TCGC-
TGGGCTTTGCCTGCGATAAATCATTCAAGTTTCTGC--CCCATCA-GCTT--
GTTGGTAGTGTATTGGACTACCATGGCGTTCACGGG-
TAACGGAGGATTAGGGTTCGATTCCGGAGAGGGAGCCTGAGAAACGGCTACCACTTCTACGGAAGGC
AGCAGGCGCGTAAATTACCCAATCCTGATTCAGGGAGGTAGTGACAAGAAATAACAGACCGGAGCCTC
GT-GCACCGGGGTTGCAAT-GAGTACAATTTA-
AATTCCTTAATGAGGACCAATTGGAGGGCAAGTCTGGTGCCAGCAGCCGCGGTAATTCCAGCTCCAAT
AGCGTATATTAAAGTTGTTGCAGTTA--AAAAGCTCGTAGTTGGATTTCTGG-----------ATATGCGC-
TGATG-TCGGC---CGTGTGCTCG-TG-CAGATGCGCGTTTCCATCCTTCTGTTAACG-
TTTCTTGGTATTCATTT----ACTGG--TTTCGGGCTCAGATATTTTACC--------------------------------------
TTGAGAAAATTAGAGTGTTTCAGGCAGGCTAGCGCC-
GGAATACATTAGCATGGAATAATAGAATAGGACTA------------------CGGTTCCTTTTGTTGGT-
TTGAGGG-CCGAAG-TAATGGTTAAT-AGGGATAGTT------------
GGGGGCATTAGTATTTAATTGTCAGAGGTGAAATTCTTTGATTTGTTAAAGACTAACCTATGCGAAAGC
A---TTTGCCAAGGATGTTTTCATTAATCA-
AGAACGAAAGTTAGGGGATCAAAGACGATCAGATACCGTCCTAGTCTTAACCATAAACTATGCCGACT
AGGGATTGGAGG-CGTGC-GAAA--TCCGCCTTCAGCACC-TTATGAGAAATCAAAGTC-
TTTGGGTTCTGGGGGGAGTATGGTCGCAAGGCTGAAACTTAAAGGAATTGACGGAAGGGCACCACCA
GGAGTGGAGCTTGCGGCTTAATTTGACTCAACACGGGAAAACTTACCAGGTCCAGACATAGTAAGGAT
TGACAGATTGATAGCTCTTTCTTGATTCTATGGG-----
TGGTGGTGCATGGCCGTTCTTAGTTGGTGGAGTGATTTGTCTGGTTAATTCCGTT-
AACGAACGAGACCTTAGCCTACTAAATAGTTAC-TACT-CCTTGGGAGT---
ATCTAACTTCTTAGAGGGACTTTGTG-TGC--
AACCACAAGGAAGTTTGAGGCAATAACAGGTCTGTGATGCCCTTAGATGTCCTGGGCCGCACGCGTGC
TACACTGACGCGTACACCAA-------GTA-TT----------CCTGCTCCGCGAGG-CAGCAGG-TAATCT--
ACAATACGCGTC-GTGATGGGGATAGA-
TCTTTGGAATTATAGATCTTGAACGAGGAATTCCTAGTAAGCGTAAATCATTAGTTTGCGCTGATTAAGT
CCCTGCCCTTTGTACACACCGCCCGTCGCTCCTACCGATTTCGAGTGATCCGGTGAACCT-
TTCGGACTGCGA-GCGTT-AGC---------
TTGTGGAATGTCAAGTAAACCTTATCACTTAGAGGAAGGAGAAGTCGTAACAAGGTT

Gastrocirrhus_monilifer ATG-
TTATACAGTGAAACTGCGAATGGCTCATTAAAACAGTTATAGTTTATTTGATATTCAAGT--------------
TTATATGGATAACCGTAGAAAAGCTAGAGCTAATACATGC-GTTACGCCTGACTTAC---
GAAGGGCAGTATTTATTAGATA-ACAAGCCAATATTCC--TCG-TGTCTATTGTGATGACTCATAATAAC--
TAATCGAA-TCGC-TGAGCTTTGCTTGCGATAAATCATTCAAGTTTCTGC--CCCATCA-GCTT--
GTTGGTAGTGTATTGGACTACCATGGCGTTCACGGG-
TAACGGAGGATTAGGGTTCGATTCCGGAGAGGGAGCCTGAGAAACGGCTACCACTTCTACGGAAGGC
AGCAGGCGCGTAAATTACCCAATCCTGACTCAGGGAGGTAGTGACAAGAAATAACAGACCGATGCCTC
GT-GCAGCGGGGTTGCAAT-GAGTACAATTTA-
AACTCCTTAATGAGGACCAATTGGAGGGCAAGTCTGGTGCCAGCAGCCGCGGTAATTCCAGCTCCAAT
AGCGTATATTAAAGTTGTTGCAGTTA--AAAAGCTCGTAGTTGGATTTCTGG-----------CAATGCGC-
TGATG-TCGGC---CGTGTGCTCG-CG-CAGATGCGCGTTTCCATCCTTCTGTTAACG-
TTTCTTGGTATTCATTT----ACTGG--TTTCGGGCTCAGATATTTTACC--------------------------------------
TTGAGAAAATTAGAGTGTTTCAGGCAGGCTTGCGCC-
GGAATACATTAGCATGGAATAATAGAATAGGACTA------------------CGGTTCCTTTTGTTGGT-
TTGAGGG-CCGAAG-TAATGATTAAT-AGGGATAGTT------------
GGGGGCATTAGTATTTAATTGTCAGAGGTGAAATTCTTTGATTTGTTAAAGACTAACCTATGCGAAAGC
A---TTTGCCAAGGATGTTTTCATTAATCA-
AGAACGAAAGTTAGGGGATCAAAGACGATCAGATACCGTCCTAGTCTTAACCATAAACTATGCCGACT
AGGGATCGGAGG-CGTGC-GAAA--TCCGCCTTCGGCACC-TTATGAGAAATCAAAGTC-
TTTGGGTTCTGGGGGGAGTATGGTCGCAAGGCTGAAACTTAAAGGAATTGACGGAAGGGCACCACCA
GGAGTGGAGCTTGCGGCTTAATTTGACTCAACACGGGAAAACTTACCAGGTCCAGACATAGTAAGGAT
TGACAGATTGATAGCTCTTTCTTGATTCTATGGG-----
TGGTGGTGCATGGCCGTTCTTAGTTGGTGGAGTGATTTGTCTGGTTAATTCCGTT-
AACGAACGAGACCTTAGCCTACTAAATAGTCAA-TGCTACCCTGGGTGC---
ATCTGACTTCTTAGAGGGACTTTGTG-TGC--
AACCACAAGGAAGTTTGAGGCAATAACAGGTCTGTGATGCCCTTAGATGTCCTGGGCCGCACGCGTGC
TACACTGACGCGTACATCGA-------GTATTC----------CCTGCTCCGCGAGG-TAGCAGG-TAATCT--
ACAATACGCGTC-GTGATGGGGATAGA-
TCTTTGGAATTATAGATCTTGAACGAGGAATTCCTAGTAAGCGCAAATCATTAGTTTGCGCTGATTAAGT
CCCTGCCCTTTGTACACACCGCCCGTCGCTCCTACCGATTTCGAGTGATCCGGTGAACCT-
TTCGGACTGCTG-------CGCAAGC-----
ATGCGGAATGTCAAGTAAACCTTATCACTTAGAGGAAGGAGAAGTCGTAACAAGGTT

Holosticha_heterofoissneri GCG-
ATTTATAGTGAAACTGCGAATGGCTCATTAAAACAGTTATAGTTTATTTGATAATCGAATT-------------
TTACATGGATAACCGTGGTAATTCTAGAGCTAATACATGCTGTTAAGCCCGACTTTT-
TGGAAGGGCTGTATTTATTAGAT--ACAAACCAATATTCCT-TCG-
GGTCTATTGTGATGATTCAATATAAC--TGATCGAA-
TCGCATGGTCTTGCACCGCGATAAATCATTCAAGTTTCTGC--CCCATCA-
GCTTTCGATGGTAGTGTATTGGACTACCATGGCTTTTACGGG-
TAACGGAGGATTAGGGTTCGATTCCGGAGAGGGAGCCTGAGAAACGGCTACCACATCTAAGGAAGGC
AGCAGGCGCGTAAATTACCCAATCCTGACTCAGGGAGGTAGTGACAAGAAATAACAGACCGGAGCCAT
CG-GTTCCGGGATTGCAAT-GAGTACAATTTA-
GACTCCTTAACAAGTAACAATTGGAGGGCAAGTCTGGTGCCAGCAGCCGCGGTAATTCCAGCTCCAAT
AGCGTATATTAAAGTTGTTGCAGTTA--AAAAGCTCGTAGTTGGATTTCTGT-----------GAGAGTGC-
AGGTG-TCGGC----TGATGCTTG-TG-CGCAGGCGCTCTTACATCCTTCTGTTAACT-
GATCTTGGCATTTTATT----GCTGG--TTCAGGGCTCAGATATTTTACC--------------------------------------

TTGAGAAAATTAGAGTGTTTCAGGCAGGCTTATGCC-
GGAATACATTAGCATGGAATAATGGAACAGGACCTGTGCGTCTTTCGGGGCGCCGGTCTCCGTTATTG
GT-TTCGGGGACTAAAG-TAATGATTAAT-AGGGATAGTT------------
GGGGGCATTAGTATTTAATAGTCAGAGGTGAAATTCTCGGATTTGTTAAAGACTAACTTATGCGAAAGC
A---TTTGCCAAGGATGTTTTCATTAATCA-
AGAACGAAAGTTAGGGGATCAAAGACGATCAGATACCGTCCTAGTCTTAACCATAAACTATGCCGACTC
GGGATCGGAGG-CGAGC-TCTA--ACAGCCTTCGGAACC-GTATGAGAAATCAAAGTC-
TTTGGGTTCTGGGGGGAGTATGGTCGCAAGGCTGAAACTTAAAGGAATTGACGGAAGGGCACCACCA
GGCGTGGAGCTTGCGGCTCAATTTGACTCAACACGGGAAAACTTACCAGGTCCAGACATAGGTAGGAT
TGACAGATTGATAGCTCTTTCTTGATTCTATGGG-----
TGGTGGTGCATGGCCGTTCTTAGTTGGTGGAGTGATTTGTCTGGTTAATTCCGTT-
AACGAACGAGACCTTAGCCTACTAACTAGTCGA-TTCAACTCCGGTTGA---
CTTTGACTTCTTAGAGGGACTTTGTGTTTT--
AAGCACAAGGAAGTTTGAGGCAATAACAGGTCTGTGATGCCCTTAGATGTCCTGGGCCGCACGCGTGC
TACACTGATGCATACAGCGA-------GCACTT---------CCCGGCCCCGAAAGG-CGGCTGG-TAATCA--
GCAATATGCATC-GTGATGGGGATAGA-
TCTTTGGAATTCTTGATCTTGAACGAGGAATGCCTAGTAGGCGCAGGTCATTACCCTGCGTCGATTAAG
TCCCTGCCCTTTGTACACACCGCCCGTCGCTCCTACCGA-TTCGAGTGATCCGGTGAACCT-
TTTGGACCGAGACTG----CTTGCAG-----
ACTTGGGAAATCAAGTAAACCACGTCACTTAGAGGAAGGAGAAGTCGTAACAAGGTT

Kiitricha_marina ATG-
ATCTACAGTGAAACTGCGAATGGCTCATTAAAACAGTTATAGTTTACTTGATAATGAATC--------------
TTACATGGATAACCGTAGTAAATCTAGGGCTAATACATGCGACCAAGCGGGACTTCA-
CGGAACCGCTGTATTTATTAGTT--ACGAGTCAATATTCCTCT---GGTCTA-TGTGGTGAGTCATAATAAC-
-TGATCGAA-TCGCCGGGTCTCCG--TGCGATACATCATTCAAGTTTCTGC--CCCATCA-GTTT--
GATGGTAGTGTATTGGACTACCATGGCATTAACGGG-
TAACGGAGAATTAGGGTTCGATTCCGGAGAGGGAGCCTGAGAAACGGCTACCACATCTACGGATGGC
AGCAGGCGCGTAAATTACCCAATCCTGATTCAGGGAGGTAGTGACAAGAAATCACGGCGAGGATGCA
CAGTCATTTTCGATTGTAAT-GTATGTAATTTA-
AATCTCTTATTGAGGAGCAATTGGAGGGCAAGTCTGGTGCCAGCAGCCGCGGTAATTCCAGCTCCAAT
AGCGTATATTAAAGTTGTTGCAGTTA--AAAAGCTCGTAGTTGGATTTCTGG-----------ATGAGATG-
CGGTGTTCATC-CTCTAAGGAAAG-AG-CTCGTGGTCTCGTTCATCCTTCTGATAACA-GTTCCGGG--
TTTAGTT----ATTCGGTTCTGCGATTCAGATATTTTACC--------------------------------------
TTGAGAAAATTAGAGTGTTTCAGGCAGGCTTGAGCCTTGAATACATTAGCATGGAATAATAGAAAAGG
ACTT------------------CTGTCT-ATTTGTTGGT-CTCGGAGACAGAAT-TAATGATTAAT-AGGGACAGTT-----
----
GGGGGCATTAGTATTTAATTGTCAGAGGTGAAATTCTTTGATTTATTAAAGACTAACTTGTGCGAAAGC
A---TTTGCCAAGGATGTTTTCATTAATCA-
AGAACGAAAGTTAGGGGATCAAAGACGATCAGATACCGTCCTAGTCTTAACCATAAACTATGCCGACT
AGGGATTGGAGGACGAAAAAAGT--TTCTCCTTCAGCACC-TTACGAGAAATCAAAGTC-
TTTGGGTTCTGGGGGGAGTATGGTCGCAAGGCTGAAACTTAAAGGAATTGACGGAAGGGCACCACCA
GGAGTGGAGCTTGCGGCTTAATTTGACTCAACACGGGAAAACTTACCAGGTCCAGACACGCGTAGGAT
TGACAGGTTGATAGTCCTTTCTTGATTGCTTGGG-----
TGGTGGTGCATGGCCGTTCTTAGTTGGTGGAGTGATTTGTCAGGTTAATTCCGTT-
AACGAACGAGACCTTAACCTACTAATTAGTTTT-TCTTGCCGAGGCAAG---
CTACAACTTCTTAGAGGGACAGAAGACA----
AAATCTTAGGAGGTTTGAGGCAATAACAGGTCTGTGATGCCCTTAGATGTCCTGGGCCGCACGCGTGC

TACACTGATGCATACAACGA-------GTAATA---------CCTTGCTCCGACAGG-AAGCGAG-TAATCC--
ATAATATGCATC-GTGATGGGGATAGA-
TCTTTGTAATTATGGATCTTGAACGAGGAATTCCTAGTAAGCGCAAGTCATTAGCTTGCGTTGATTAAGT
CCCTGCCCTTTGTACACACCGCCCGTCGCTCCTACCGA--TCGGGTGATCCGGTGAACCT-
TTTGGACCATTA-------------------
TTATGGGAAATTAAGTAAGCCTTGTCACTTAGAGGAAGGAGAAGTCGTAACAAGGTT

Licnophora_lyngbycola GCG-
ACTTACAGTGAAACTGCGAATGGCTCATTAAACCAGTTATAGTTTATATGATA---GATGT-------------
TTAGACGGATAACCGTGGTAATTCTAGAGCTAATACGTGCGTGAAGGCTCGACTCCT-
TGGTAGGGCTGCATTTATTAGATA---AAGCCAA--CTCCGCTTGCGGACG--
TGTGGTGAGTCATAGTAAC--TGAGCGAA-
TCGCAAGGCCGATGGCCGCGATAAGTCATGCAAGTTTCTGC--CCTATCA-
GCTGTCGATGGTAGTGTATTGGACTACCATGGCGTTAACGGG-
TAACGGAGAATTAGGGTTCGATTCCGGAGAGGGAGCCTGAGAAACGGCTACCACATCTAAGGAAGGC
AGCAGGCGCGTAAATTACCCAATCCTGACTCAGGGAGGTAGTGACAAGAAATAACGGAGCTTTGCCTC
ACGGCATTGTGATCGTAATCGCGGTTAATTTA-
CACCCCTTAACAAATATTCATTGGAGGGCAAGTCTGGTGCCAGCAGCCGCGGTAATTCCAGCTCCAATA
GCGTATATTAAAATTGTTGCAGTTA--AAAAGCTCGTAGTTGGATTTCTGC-----------GGGCGCTG-
CGTCGACTCCC------GAGTGGA-GA-
AGACGGGGCGCGCGCATCGTTCTGTCAGCACGGGTTTGATTCTCACGG----GTCGA--
GCTCGCGCGCAGATGTTTTACC-------------------------------------
TTGAGGAAATTAGAGTGTTTCAGGCAGGCTTA-GCC-GGAATACGCTAGCATGGAATAATGGCG-
AGGACTT-----------------GGGCCCCATTCGTTGGT-TTGGGGGCCAGAG--TAATGGTTAAT-
AGGGACAGTT------------
GGGGGCATTAGTATTTAATTGTCAGAGGTGAAATTCTTGGATTTTTTAAAGACTAACCTGTGCGAAAGC
A---TTTGCCAAGGATGTTTTCATTAATCA-
AGAACGAAAGTTAGGGGATCAAAGACGATCAGATACCGTCCTAGTCTTAACCATAAACTATGCCGACTC
GGGATTGGG---CGAGT--CTT--CACTCGTTCAGCACC-GTCCGAGAAATCAAAGTC-
TTTGGGTTCTGGGGGGAGTATGGTCGCAAGGCTGAAACTTAAAGGAATTGACGGAAGGGCACCACCA
GGAGTGGAGCCTGCGGCTTAATTTGACTCAACACGGGGAAACTCACCTGGTCAAGACACAGGAAGGA
TTGACAGATTGATAGCTCTTTCTCGATTCTGTGGT-----
AGGTGGTGCATGGCCGTCCTTAGTTGGTGGAGTGATTTGTCAGGTTAATTCCGTT-
AACGAACGAGACATGTGCCTGCTAACTAGTCCT-GTCTCCTTCTGGAGC---
AGTAGACTTCTTAGAGGGACTTTGCG-TGC--
AAGCGCAAGGAAGCTACATGCAATGACAGGTCTGTGATGCCCCCAGACGTCCAGGGCCGCACGCGTG
CTACATTGAGGCCATCAACAA-------GCACTG-----------CTGCGCCGAAAGG-ACGCGGC-TAATCT--
CTAGTTGGCCTC-GCGATGGGGATAGA-
TCTTTGCAATTTTAGATCTTGAACGAGGAATTCCTAGTAGGCGCGAGTTAGCATCTCGCGCCGATTACG
TCCCTGCCCTTTGTACACACCGCCCGTCGCTTCTACCGA--TTGAGTGGTCCGGTGAACCC-TTCGGAC----
---GCGCTCCCGCGC----------
AAAAGTCGAGTGAACCTTGCCACTTAGAGGAAGAAGAAGTCGTAACAAGGTA

Licnophora_macfarlandi GCG-
CTTTACAGTGAAACTGCGAATGGCTCATTAAAACAGTTATAGTTTATTTGATA---GATGT-------------
TTAGACGGATAACCGTGGTAATTCTAGAGCTAATACGTGCGTGAAGGCCCGACTCCT-
CGGAAGGGCTGCATTTATTAGATA---AAGCCAACTCCTC------CGGGAAGTGTGGTGAATCATAGTAAC-
-TGAGCGAA-TCGCATGGTCAATGACCGCGATAAGTCATTCAAGTTTCTGC--CCTATCA-
GCTGTCGATGGTAGTGTATTGGACTACCATGGCTTTAACGGG-

TAACGGAGAATTAGGGTTCGATTCCGGAGAGGGAGCCTGAGAAACGGCTACCACATCTAAGGAAGGC
AGCAGGCGCGTAAATTACCCAATCCTGACTCAGGGAGGTAGTGACAAGAAATAACGGAGCTTTGCCCT
AG-GCATTGTGATCGTAAT-GAGGTTAACTTA-AAGCCCTTACCGAA-
ACCTATTGGAGGGCAAGTCTGGTGCCAGCAGCCGCGGTAATTCCAGCTCCAATAGCGTATATTAAAGTT
GTTGCAGTTA--AAAAGCTCGTAGTTGGATTTCTGG--------------------
GGACACGCCGACTTGGTACGTTTACCA-CGGAAGCGTGGACTCATCGTTCCG-CAGCG---
AAAGTTAATCTCCGG----ATCGACTTCCCGCGCGGA--TGTTTTACC--------------------------------------
TTGAGGAAATTAGAGTGTTTCAGGCAGGCTTG-GCC-AGGATACGCTAGCATGGAATAATT-
AGGAGGACTT------------------AGGCCTCACTCGTTGGT-TTGGGGGTCCGAG--TAATGGTTAAT-
AGGGACAGTT------------
GGGGGCATTAGTATTTAATTGTCAGAGGTGAAATTCTTGGATTTTTTAAAGACTAACCTGTGCGAAAGC
A---TTTGCCAAGGATGTTTTCATTAATCA-
AGAACGAAAGTTAGGGGATCAAAGACGATCAGATACCGTCCTAGTCTTAACCATAAACTATGCCAACT
GGGGATCGGGAG----AGTTTCT--CACTCTTTCGGCACC-CTCCGAGAAATCAAAGTC-
TTTGGGTTCTGGGGGGAGTATGGTCGCAAGGCTGAAACTTAAAGGAATTGACGGAAGGGCACCACCA
GGAGTGGAGCCTGCGGCTTAATTTGACTCAACACGGGGAAACTCACCTGGTCCAGACACAGGAAGGAT
TGACAGATTGATAGCTCTTTCTCGATTCTGTGGG-----
TGGTGGTGCATGGCCGTTCTTAGTTGGTGGAGTGATTTGTCAGGTTAATTCCGTT-
AACGAACGAGACCTTAGCCTGCTAACTAGTCCAGTCCTCCTGCGGGAGA---
TTGCGACTTCTTAGAGGGACTTTGTGTGTTAAAAGCATAAGGAAGTTTGAGGCAATAACAGGTCTGTG
ATGCCCTTAGATGTCCAGGGCCGCACGCGTGCTACATTGAGGCCGTCAACAA-------GCATTG-----------
CTGCGCCGAAAGG-ACGCGGC-TAATCT--CTAGTCGGTCTC-GTGATGGGGATAGA-
TCTTTGTAATTATAGATCTTCAACGAGGAATTCCTAGTAGGCGTGAGTCAGCATCTCGCGCCGATTACGT
CCCTGCCCTTTGTACACACCGCCCGTCGCTCCTACCGA--TTGAGTGGTCCGGTGAATCC-TTCGGACA----
-CGTGTTCTCATGT----------
GAAAGTCGAGTGAACCTTACCACTTAGAGGAAGGAGAAGTCGTAACAAGGTA

Metaurostylopsis_antarctica ATA-
TTATACAGTGAAACTGCGAATGGCTCATTAAAACAGTTATAGTTTATTTGATAATCAAAT--------------
TTACATGGATACCCGTGGTAATTCTAGAGCTAATACATGCTGGTTAGCCCGACTTCT-
TGGAAGGGCTGTATTTATTAGATA-ACAAGCCAATATTCC--TCG-
TGTCTATTGTGACGACTCATAATAAC--TGATCGAA-
TCGCATGGACTTTGTCCGCGATACATCATTCAAGTTTCTGC--CCCATCA-
GCTTTCGATGGTAGTGTATTGGACTACCATGGCTCTTACGGG-
TAACGGAGGATTAGGGTTCGATTCCGGAGAGGGAGCCTGAGAAACGGCTACCACATCTAAGGAAGGC
AGCAGGCGCGTAAATTACCCAATCCTAATTCAGGGAGGTAGTGACAAGAAATAACGAGCTGAGGCCTT
T--GCTTCAGGATTGCAAT-GAGTAGAATTTA-
AACCCCTTTTCGAGGATCAATTGGAGGGCAAGTCTGGTGCCAGCAGCCGCGGTAATTCCAGCTCCAATA
GCGTATATTAAAGTTGTTGCAGTTA--AAAAGCTCGTAGTTGGATTTCTGA-----------GGGATTAT-
CAAAG-TCCGC----TGATGCGTG-TA-CTGAGAGGATCCCTCATCCTTCTGTTAACC-
CTTCTTGGTATTCATTT----ACTGG--TTGGGGGCTCAGATATTTTACC--------------------------------------
TTGAGAAAATTAGAGTGTTCCAGGCAGGCTTGCGCC-
GGAATACATTAGCATGGAATAATAGAAAAGGACTT------------------GGGTCTCCTTCGTTGGT-
TTGAGGGGCACTAG-TAATGATTAAT-AGGGACAGTT------------
GGGGGCATTAGTATTTAATTGTCAGAGGTGAAATTCTCGGATTTGTTAAAGACTAACTTATGCGAAAGC
A---TTTGCCAAGGATGTTTTCATTAATCA-
AGAACGAAAGTTAGGGGATCAAAGACGATCAGATACCGTCGTAGTCTTAACCATAAACTATGCCGACT
AGGGATCGGAGG-TGTGC-TATA--TCCGCCTTCGGCACC-TTATGAGAAATCAAAGTC-

TTTGGGTTCTGGGGGGAGTATGGTCGCAAGGCTGAAACTTAAAGGAATTGACGGAAGGGCACCACCA
GGAGTGGAGCTTGCGGCTCAATTTGACTCAACACGGGAAAACTTACCAGGTCCAGACATTTTGAGGAT
TGACAGATTGATAGCTCTTTCTTGATTCTATGGG-----
TGGTGGTGCATGGCCGTTCTTAGTTGGTGGAGTGATTTGTCTGGTTAATTCCGTT-
AACGAACGAGACCTTAGCCTGCTAACTAGTCAG-CGCAATCTC-ATTGC---
ATTTGACTTCTTAGAGGGACTTTGCGTTTT--
AAGCGCAAGGAAGTTTGAGGCAATAACAGGTCTGTGATGCCCTTAGATGTCCTGGGCCGCACGCGTGC
TACACTGATGCATACAGCGA-------GTACTT---------TCCAGCTCCGCGAGG-CTGCTGG-TAATCA--
GCAATATGCATC-GTGCTGGGGATAGA-
TCTTTGGAATTATAGATCTTGAACGAGGAATTCCTAGTAAGCGCAAGTCATTAGCTTGCGCTGATTAAG
TCCCTGCCCTTTGTACACACCGCCCGTCGCTCCTACCGATTTCGAGTGATTCGGTGAACCT-
TTTGGACAGCG--TCGGGTCTCGTGCCAG--
ATGCTGGAAGTCAAGTAAACCATATCACTTAGAGGAAGGAGAAGTCGT?????????

Oxytricha_granulifera ATG-
TTATACAGTGAAACTGCGAATGGCTCATTAAAACAGTTATAGTTTATTTGATAATCAAA-C--------------
TTACATGGATAACCGTGGTAATTCTAGAGCTAATACATGCTGTTTAGCCTGACTTTT-
AGGAAGGGCTGTATTTATTAGATA-ACAAACCAATATTCC--TTG-
TGTCTATTGTGATGACTCATAATAAC--TGATCGAA-
TCGCATGGACTTTGTCCGCGATACATCATTCAAGTTTCTGC--CCCATCA-
GCTTTCGATGGTAGTGTATTGGACTACCATGGCTCTTACGGG-
TAACGGAGGATTAGGGTTCGATTCCGGAGAGGGAGCCTGAGAAACGGCTACCACATCTAAGGAAGGC
AGCAGGCGCGTAAATTACCCAATCCTGATTCAGGGAGGTAGTGACAAGAAATAACGGACCGAAGCAC
AT--GTTTCGGGATTGCAAT-GAGTAGAATTTA-
AACCCCTTTACGAGGATCAATTGGAGGGCAAGTCTGGTGCCAGCAGCCGCGGTAATTCCAGCTCCAAT
AGCATATATTAAAGTTGTTGCAGTTA--AAAAGCTCGTAGTTGGATTTCTGG-----------GAGGGTGC-
TGATG-TCCGC--ATGTTTGCGTG-TG-CAG-AGCGCCCTCCCATCCTTCTGTTAACG-
TTTCTTGGTATTCATTT----ACTGG--TTTCGGGCTCAGATATTTTACC--------------------------------------
TTGAGAAAATTAGAGTGTTCCAGGCAGGCTTGCGCC-
GGAATACATTAGCATGGAATAATAGAATAGGACTT------------------TAGTCTCCTTTGTTGGT-
TTGAGGGATTGAAG-TAATGATTAAT-AGGGATAGTC------------
GGGGGCATTAGTACTTAATTGTCAGAGGTGAAATTCTCGGATTTGTTAAAGACTAACTTATGCGAAAGC
A---TTTGCCAAGGATGTTTTCATTAATCA-
AGAACGAAAGTTAGGGGATCAAAGACGATCAGATACCGTCCTAGTCTTAACCATAAACTATGCCGACT
AGGGATCGGAGG-CGCGC-ATTA--TCCGCCTTCGGCACC-TTATGAGAAATCAAAGTC-
TTTGGGTTCTGGGGGGAGTATGGTCGCAAGGCTGAAACTTAAAGGAATTGACGGAAGGGCACCACCA
GGAGTGGAGCTTGCGGCTCAATTTGACTCAACACGGGAAAACTTACCAGGTCCAGACATAGTGAGGAT
TGACAGATTGATAGCTCTTTCTTGATTCTATGGG-----
TGGTGGTGCATGGCCGTTCTTAGTTGGTGGAGTGATTTGTCTGGTTAATTCCGTT-
AACGAACGAGACCTTAGCCTACTAACTAGTCGA-ACCAATTCCGATTGG---
CTCCGACTTCTTAGAGGGACTTTGTG-ACT--
AAACACAAGGAAGTTTGAGGCAATAACAGGTCTGTGATGCCCTTAGATGTCCTGGGCCGCACGCGTGC
TACACTGACACATACAACGA-------GTACAT---------CCCAGCTCCGAGAGG-CAGCTGG-TAATCA--
GCAATATGTGTC-GTGATGGGGATAGA-
TCTTTGGAATTATAGATCTTGAACGAGGAATTCCTAGTAAGCGCAAGTCATTACCTTGCGCTGATTAAGT
CCCTGCCCTTTGTACACACCGCCCGTCGCTCCTACCGATTTCGAGTGATCCGGTGAACCT-
TTTGGACTGCGC-GGAGT-CTCGTGCTCT--
GTGTGGAAAATCAAGTAAACCATATCACTTAGAGGAAGGAGAAGTCGTAACAAGGTT

P_metchnikoffi_Wuhan
??????????????????????????????ATTAAAACAGTTATAGTTTATTTGATAATGGAAAT--------------
TTACATGGATAACCGTGGTAATTCTAGAGCTAATACATGCTGTTAAGCCTGACTTTG-
CGGAAGGGCTGTATTTATTAGATA-
AAAGACCAATATTCCCGCAAGGGTCTATTGTGATGATTCATAATAAC--TGATCGAA-
TCGCATAGGCTTTGCCTGCGATAAATCATTCAAGTTTCTGC--CCTATCA-
GCTTTCGATGGTAGTGTATTGGACTACCATGGCTTTAACGGG-
TAACGGGGAATTAGGGTTCGATTCCGGAGAGGGAGCCTGAGAAACGGCTACCACTTCTAAGGAAGGC
AGCAGGCGCGTAAATTACCCAATCCTAACTCAGGGAGGTAGTGACAAGAAATAACAACGCAAGGCTAT
---GCCTTGCGATTGGAAT-GAGTACAATTTA-
AACCCCTTAACGAGGATCAATTGGAGGGCAAGTCTGGTGCCAGCAGCCGCGGTAATTCCAGCTCCAAT
AGCGTATATTAAAGTTGTTGCAGTTA--AAAAGCTCGTAGTTGGATTTCTGG-----------GAGGGTGG-
TCTCGCTCCGC--CTGCGGGCGTG-AG-TGAGATTGCCCTCCCATCCTTCTGTTAACG-
ATTCTTGGTATTCATTT----ACTGG--TTTCGGGCTCAGATATTTTACC--
TTGAGAAAATTAGAGTGTTTAAGGCAGGCTTGCGCC-
GGAATACATTAGCATGGAATAATAGAATAGGACTT------------------
TAGTCCCTTTTGTTGGTCTTCAGGGGCTGAAG-TAATGATTAAT-AGGGATAGTT------------
GGGGGCATTAGTATTTAATTGTCAGAGGTGAAATTCTCGGATTTGTTAAAGACTAACATATGCGAAAGC
A---TTTGCCAAGGATGTTTTCATTAATCA-
AGAACGAAAGTTAGGGGATCAAAGACGATCAGATACCGTCGTAGTCTTAACCATAAACTATGCCGACT
CGGGATTGGAGA-TGTTA--TTA--TACTTCTTCAGCACC-GTATGAGAAATCAAAGTC-
TTTGGGTTCTGGGGGGAGTATGGTCGCAAGGCTGAAACTTAAAGGAATTGACGGAAGGGCACCACCA
GGAGTGGAGCTTGCGGCTTAATTTGACTCAACACGGGAAAACTTACCAGGTCCAGAAGTAGGTAGGAT
TGACAGATTGATAGCTCTTTCTTGATTCTATGGG-----
TGGTGGTGCATGGCCGTTCTTAGTTGGTGGAGTGATTTGTCTGGTTAATTCCGAT-
AACGAACGAGACCTTAACCTGCTAACTAGTTCT-GCGAATTGCGATTTG---
CAGCAACTTCTTAGAGGGACAGTGTA-CGC--
AAGTACAAGGAAGTTTGAGGCAATAACAGGTCTGTGATGCCCTTAGATGTCCTGGGCCGCACGCGTGC
TACACTGATGCATACAACGA-------GC-CTA---------TCCTGCCCCGAGAGG-CGGCGGG-TAATCT--
TCAATATGCATC-GTGATGGGGATAGA-
TCTTTGGAATTATAGATCTTCAACGAGGAATTCCTAGTAAGCGCGAGTCATTAGCTCGCGCTGATTACG
TCCCTGCCCTTTGTACACACCGCCCGTCGCTCCTACCGATTTCGAGTGATGAGGTGAACCT-
TTAGGACTGCG--------CTCAC-------
GTGCGGAAATTCAAGTAAACCTTATCACTTAGAGGAAGGAGAAGTCGTAACAAGGTT

Phacodinium_metchnikoffi ATG-
GTATACAGTGAAACTGCGAATGGCTCATTAAAACAGTTATAGTTTATTTG-TAATGGAAAT--------------
TTACATGGATAACCGTGGTAATTCTAGAGCTAATACATGCTGTTAAGCCTGACTTTG-
CGGAAGGGCTGTATTTATTAGATA-AAAGACCAATATTCCCGCAA-
GGTCTATTGTGATGATTCATAATAAC--TGATCGAA-
TCGCATAGGCTTTGCCTGCGATAAATCATTCAAGTTTCTGC--CCTATCA-
GCTTTCGATGGTAGTGTATTGGACTACCATGGCTTTAACGGG-
TAACGGGGAATTAGGGTTCGATTCCGGAGAGGGAGCCTGAGAAACGGCTACCACTTCTAAGGAAGGC
AGCAGGCGCGTAAATTACCCAATCCTAACTCAGGGAGGTAGTGACAAGAAATAACAACGCAAGGCTAT
---GCCTCGCGATTGGAAT-GAGTACAATTTA-
AACCCCTTAACGGGGCTCAATTGGAGGGCAAGTCTGGTGCCAGCAGCCGCGGTAATTCCAGCTCCAAT
AGCGTATATTAAAGTTGTTGCAGTTA--AAAAGCTCGTAGTTGGATTTCTGG-----------GAGGGTGG-
TCTCGCTCCGCCTGCGGGCGTGAG-TG-AG--ATTGCCCTCCCATCCTTCTGTTAACG-

ATTCTTGGTATTCATTT----ACTGG--TTTCGGGCTCAGATATTTTACC--------------------------------------
TTGAGAAAATAAGAGTGTTTAAGGCAGGCTTGCGCC-
GGAATACATTAGCATGGAATAATAGAATAGGAACT------------------
TTAGTCCTTTTGTTGGTCTTCAGGGTCTGAAGTTAATGATTAATAAGGGATAGTT------------
GGGGGCATTAGTATTTAATTGTCAGAGGTGAAATTCTCGGATTTGTTAAAGACTAACATATGCGAAAGC
A---TTTGCCAAGGATGTTTTCATTAATCA-
AGAACGAAAGTTAGGGGATCAAAGACGATCAGATACCGTCGTAGTCTTAACCATAAACTATGCCGACT
CGGGATTGGAGA-TGTTATTATA--CTTTCTTTCAGCACC-GTATGAGAAATCAAAGTC-
CTTGGGTTCTGGGGGGAGTATGGTCGCAAGGCTGAAACTTAAAGGAATTGACGGAAGGGCACCACCA
GGAGTGGAGCTTGCGGCTTAATTTGACTCAACACGGGAAAACTTACCAGGTCCAGAAGTAGGTAGGAT
TGACAGATTGATAGCTCTTTCTTGATTCTATGGG-----
TGGTGGTGCATGGCCGTTCTTAGTTGGTGGAGTGATTTGTCTGGTTAATTCCGAT-
AACGAACGAGACCTTAACCTGCTAACTAGTTCT-GCGAATTGCGATTTG---
CAGCAACTTCTTAGAGGGACAGTGTA-CGC--
AAGTACAAGGAAGTTTGAGGCAATAACAGGTCTGTATTGCCCTTAGATGTCCTGGGCCGCACGCGTGC
TACACTGATGCATACAACGA-------GCCTAT----------CCTGCCCCGAGAGGCCGGCGGGTTAATCT--
TCAATATGCATTAGTGATGGGGATAGATTCTTTGGAATTATAGATCTTCAACGAGGAATTCCTAGTAAG
CGCGAGTTATTA-
CTCGCGCTGATTACGTCCCTGCCCTTTGTACACACCGCCCGTCGCTCCTACCGATTTCGAGTGATGAGGT
GAACCT-TTAGGACTGCG--------CTCAC-------
GTGCGGAAATTCAAGTAAACCTTATCACTTAGAGGAAGGAGAAGTCGTAACAAGGTT

Prodiscocephalus_borrori
GCGATTATACAGTGAAACTGCGAATGGCTCATTAAAACAGTTATAGTTTATTTGATATCCGAATT---------
-----TTATATGGATAACCGTGGTAATTCTAGAGCTAATACATGCATTCAGGCCCGATT----
CGGTAAGGCTGTATTTATTAGATA-ACAAATCAATATTCCT-TCG-
GGTCTATTTTGATGATTCATAATAAC--TGATCGAA-TCGC--GATCTTT---
CGCGATAAATCATTCAAGTTTCTGC--CCCATCA-
GCTTTCGATGGTAGTGTATTGGACTACCATGGCTCTTACGGG-
TAACGGAGGATTAGGGTTCGATTCCGGAGAGGGAGCCTGAGAACCGGCTACCACATCTAAGGAAGGC
AGCAGGCGCGTAAATTACCCAATCCTGATTCAGGGAGGTAGTGACAAGAAATAACGGACCGGAGCTTT
GT-GCACCGGGATTGCAAT-GAATACAATTTA-
AACCCCTTAATGAGGAACAATTGGAGGGCAAGTCTGGTGCCAGCAGCCGCGGTAATTCCAGCTCCAAT
AGCGTATATTAAAGTTGTTGCAGTTA--AAAAGCTCGTAGTTGGATTTCTGG-----------GTTAGTGT-
CAAAG-TCGATCAGACGATCTGTG----CTGCGATACTGGTCCATCCTTCTGTTAACG-
ATTCTTGGTATTCATCT----ACTGG--TTTCGGGCTCAGATATTTTACC--------------------------------------
TTGAGAAAATTAGAGTGTTCCAGGCAGGCTTGCGCC-
GGAATACATTAGCATGGAATAGTAGAATAGGACTT------------------TTGTCTCTTTTGTTGGT-
TTGAGGGACTGAAG-TAATGATTAAT-AGGGATAGTT------------
GGGGGCATTAGTATTTAATTGTCAGAGGTGAAATTCTTGGATTTGTTAAAGACTAACTTATGCGAAAGC
A---TTTGCCAAGGATGTTTTCATTAATCA-
AGAACGAAAGTTAGGGGATCAAAGACGATCAGATACCGTCCTAGTCTTAACCATAAACTATGCCGACTC
GGGATTGGAGG-TGATC-TTCA--TATACCTTCAGCACC-GTATGAGAAATCAAAGTC-
TTTGTGTTCTGGGGGGAGTATGGTCGCAAGGCTGAAACTTAAAGGAATTGACGGAAGGGCACCACCA
GGCGTGGAGCTTGCGGCTTAATTTGACTCAACACGGGAAAACTTACCAGGTCCAGACATAGTGAGGAT
TGTCAGATTGATAGCTCTTTCTTGATTCTATGGG-----
TGGTGGTGCATGGCCGTTCTTAGTTGGTGGAGTGATTTGTCTGGTTAATTCCGTT-
AACGAACGAGACCTTAGCCTACTAAATAGTTAC-CCCGATCTCGATTGG---

TGTAATCTTCTTAGAGGGACTTTGTGCA----
AAACACAAGGAAGTTTGAGGCAATAACAGGTCTGTGATGCCCTTAGATGTCCTGGGCCGCACGCGTGC
TACACTGACAAGTACAGCGA-------GTACGT---------CCCTGCTCCGCGAGG-CAGCAGG-TAATCT--
ACAATACTTGTC-GTGATGGGGATAGA-
TCTTTGTAATTCTTGATCTTGAACGAGGAATGCCTAGTAAGCGCGAGTCATTAACTCGCGTTGATTAAGT
CCCTGCCCTTTGTACACACCGCCCGTCGCTCCTACCGATTTCGAGTGTTCCGGTGAGTCC-
TTAGGACTGTCGGCCGGC-CTTGTGTCGCG-
TCGTGGAAATTTGTGCAAACCATAACACTTAGAGGAAGGAGAAGTCGTAACAAGGTT

Propecingulum_fistoleramalliei
??
??
??
??TGGACTACCATGGCATTTACGGG-
TAACGGAGAATTAGGGTTCGATTCCGGAGAGGGAGCCTGAGAAACGGCTACCACATCTAAGGAAGGC
AGCAGGCGCGTAAATTACCCAATCCTGATTCAGGGAGGTAGTGACAAGAAATAACGGACCGAGGCCTT
CGGGCTTCGGGATTGCAAT-GAGCCGAATTTA-
CACCCCTTAGCGAGGATCGATTGGAGGGCAAGTCTGGTGCCAGCAGCCGCGGTAATTCCAGCTCCAAT
AGCGTATATTAAAGTTGTTGCAGTTA--AAAAGCTCGTAGTTGGATTTCTGG-----------GAGGGTGC-
CTATG-TCCGC--CAACGTGCGTG-TG-CAGCGGCGCCCTTCCATCCTTCTGTTAACG-
CTTCTCGGCCTTTACTG----GCTGG--TTGCGGGCTCAGATATTTTACC--------------------------------------
TTGAGAAAATTAGAGTGTTTCAGGCAGGCTAG-GCC-
GGAATACATTAGCATGGAATAATGGAATAGGACTA-----------------CGGTCT-CTTTGTTGGT-
TTGAGGGACTGCAG-TAATGATTAAT-AGGGATAGTT------------
GGGGGCATTAGTACTTAACTGTCAGAGGTGGAATTCTCGGATTTGTTAAAGACTAACTTATGCGAAAGC
A---TTTGCCAAGGATGTTTTCATTAATCA-
AGAACGAAAGTTAGGGGATCAAAGACGATCAGATACCGTCCTAGTCTTAACCATAAACTATGCCGACTC
GGGATTGGGGG-CGCGA-ACTA--TCCGCCTTCAGCACC-GTATGAGAAATCAAAGTC-
TTTGGGTTCTGGGGGGAGTATGGTCGCAAGGCTGAAACTTAAAGGAATTGACGGAAGGGCACCACCA
GGCGTGGAGCTTGCGGCTCAATTTGACTCAACACGGGAAAACTTACCAGGTCCAGACATAGTGAGGAT
TGACAGATTGATAGCTCTTTCTTGATTCTATGGG-----
TGGTGGTGCATGGCCGTTCTTAGTTGGTGGAGTGATTTGTCTGGTTAATTCCGTT-
AACGAACGAGACCTTAGCCTGCTAACTAGACGC-GGCGATCTCGATCGT---
CGTCGTCTTCTTAGAGGGACTTTGTG-ATCT-
AAACACAAGGAAGTTTGAGGCAATAACAGGTCTGTGATGCCCTTAGATGTCCTGGGCCGCACGCGTGC
TACACTGACGCCTGCAGCGA-------GTAGTT---------CCCAGCCCCGAGAGG-TGGCTGG-TAATCA--
GCAATCGGCGTC-GTGATGGGGATAGA-
TCTTTGGAATTATGGATCTTGAACGAGGAATGCCTAGTAAGCGTAGGTCATTAGCCTACGCTGATTAAG
TCCCTGCCCTTTGTACACACCGCCCGTCGCTCCTACCGATTTCGAGTGGTCCGGTGAATCT-
TTCGGACTGCGG-TG-GC-CTCGTGCT-G--
TCGCGGAAAGTCTGGTAAACC????????????????????????????????????

Pseudoamphisiella_alveolata GCG-
TTATACTGTGAAACTGCGAATGGCTCATTAAAACAGTTATAGTTTCTGTGA-ATTCGAATT-------------
TTATATGGATAACCGTGGTAAATCTAGAGCTAATACATGCTGTCTGGCCTGTCTTCT-CGG-
AGGGCCGTATTTATTAGATA-ACAAACCAATATTCCT-CAC-GGTCTATTGTGGTGATTCATAATAAC--
TGATCGGA-TCGCATGGGCCTTGCCCGCGATAGTTCATTCAAGTTTCTGC--CCCATCA-
GCTCTCGATGGTAGTGTATTGGACTACCATGGCTTTCACGGG-
TAACGGAGGATTAGGGTTCGATTCCGGAGAGGGAGCCTGAGAAACGGCTACCACATCTAAGGAAGGC

AGCAGGCGCGTAAATTACCCAATCCTGATTCAGGGAGGTAGTGACAAGAAATAACAGACTAG----
TTAACAAACTAGGATTGCAAT-GAACCGAATTTA-
AAACCCTTAGTGAGGAGCAATTGGAGGGCAAGTCTGGTGCCAGCAGCCGCGGTAATTCCAGCTCCAAT
AGCGTATATTAAAGTTGTTGCAGTTA--AAAAGCTCGTAGTTGGATTTCTGA-----------TGGAGTTC-
CAAAG-TCCGG--TCA--CACGTG-CG-CTGCGGAGCTCTATCATCCTTCTGTTAACG-
TTTCTTGGTATTCACTT----ACTGG--TTACGGGCTCAGATACTTTACC--------------------------------------
TTGAGAAAATTAGAGTGTTTCAGGCAGGCCTGCGCC-
GGAATACATTAGCATGGAATAATAGAATAGGACTG------------------TTGTCTCTTTTGTTGGT-TC-
AGGGATGATAG-TAATGATTAAT-AGGGATAGTT------------
GGGGGCATTAGTATTTAATTGTCAGAGGTGAAATTCTTGGATTTGTTAAAGACTAACTTATGCGAAAGC
A---TTTGCCAAGGATGTTTTCATTAATCA-
AGAACGAAAGTTAGGGGATCAAAGACGATCAGATACCGTCCTAGTCTTAACCATAAACTATGCCGACT
AGGGATCGGAGG-TGAGC--TAA--TCTACCTTCGGCACC-TTATGAGAAATCAAAGTC-
TTTGGGTTCTGGGGGGAGTATGGTCGCAAGGCTGAAACTTAAAGGAATTGACGGAAGGGCACCACCA
GGAGTGGAGCTTGCGGCTTAATTTGACTCAACACGGGAAAACTTACCAGGTCCAGACATAGTGAGGAT
TGACAGATTGATAGCTCTTTCCTGATTCTATGGG-----
TGGTGGTGCATGGCCGTTCTTAGTTGGTGAAGTGATTTGTCTGGTTAATTCCGTT-
AACGAACGAGACCTTAGCCTGCTAACTAGTAGCGCTCGGCTCTTGCCGA---
CGCAGACTTCTTAGAGGGACTTTGTG-
GTCATAAACACAAGGAAGTTTGAGGCAATAACAGGTCTGTGATGCCCTTAGATGTCCTGGGCCGCACG
CGTGCTACACTGACGCATACAACGA-------GTAATC---------GCCTGCTCCGCGAGG-CTGCAGG-
TAATCT--ACAATATGCGTC-GTGATGGGGATAGA-
TCTTTGGAATTATAGGTCTTGAACGAGGAATTCCTAGTAAGCGCAAGTCATTAGCTTGCGCTGATTAAG
TCCCTGCCCTTTGTACACACCGCCCGTCGCTCCTACCGATTTCGAGTGATCCGGTGAACCT-
CTCGGACTGCGC-GCGGCGCTCGTCGTCGC-
GAGCGGGAAGTTAAGTAAACCAAATCACTTAGAGGAAGGAGAAGTCGTAACAAGGTT

Stenosemella_pacifica ATG-
GTATACAATGAAACTGCGAATGGCTCATTAAAACAGTTATAGTTTATTTGGTAATCAAAC---------------
TTACATGGATAACCGTGGTAATTCTAGAGCTAATACATGCTGTTGTGCCCGACTCAC---
GAAGGGCCGTATTTATTAGAT--ATCAGCCAATAAGCAT-----CTGCTATTGTGGTGACTCATAGTAAC-
TTAATCGGA-TCGCATGGGCTTGTCCCGCGACAAACCATTCAAGTTTCTGC--
CCCATCATGCTTTCGATGGTAGTGTATTGGACTACCATGGCTTTCACGGG-
TAACGGAGGATTAGGGTTCGATTCCGGAGAGGGAGCCTGAGAAACGGCTACCACTTCCACGGAAGGC
AGCAGGCGCGTAAATTACCCAATCCTGATTCAGGGAGGTAGTGACAAGAAATAACGGGTCGGGGTTTT
---GCCCCGGGACTGCAAT-GAGACGAATTTA-
GACCCCTTATCGATCAACAATTGGAGGGCAAGTCTGGTGCCAGCAGCCGCGGTAATTCCAGCTCCAATA
GCGTATATTAAAGTTGTTGCAGTTA--AAAAGCTCGTAGTTGGATTTCTGG-----------
TAGTGGGTTCGAGC--------GCACATGCGTGATGTCTCATGCCCGTTGCCATCCTTCTGTTAACC-
TATCAGGGCATTCATTT----GTCAG--TTAGGGGCTCAGATATTTTACC--------------------------------------
TTGAGAAAATTAGAGTGTTTCAGGCAGGCTTGTGCC-
CGGATACATTAGCATGGAATAATGGAATAGGACTT------------------TAGCCTCTTCTGTTGGT-
CTGAGGGGTTGAAG-TAATGATTAAT-AGGGATAGTT------------
GGGGGCATTAGTACTTAACTGTCAGAGGTGAAATTCTCGGATTTGTTAAAGACTAACTTATGCGAAAGC
A---TTTGCCAAGGATGTTTTCATTAATCA-
AGAACGAAAGTTAGGGGATCAAAGACGATCAGATACCGTCCTAGTCTTAACCATAAACTATGCCGACTC
GGGATCGGGGG-CCAGA-GTTA--ACTGCCTTCGGCACC-GTATGAGAAATCAAAGTC-
TTTGGGTTCTGGGGGGAGTATGGTCGCAAGGCTGAAACTTAAAGGAATTGACGGAAGGGCACCACCA

GGCGTGGAGCTTGCGGCTCAATTTGACTCAACACGGGGAAACTTACCAGGTCCAGACATAGTGAGGAT
TGACAGATTGATAGCTCTTTCTTGATTCTATGGG-----
TGGTGGTGCATGGCCGTTCTTAGTTGGTGGAGTGATTTGTCTGGTTAATTCCGTT-
AACGAACGAGACCTTAGCCTACTAAATAGTCGC-AACAATTTCTATTGG---
CTGCGTCTTCTTAGAGGGACTTTGCA-AGC--
AACTGCAAGGAAGTTTGAGGCAATAACAGGTCTGTGATGCCCTTAGATGTCCTGGGCCGCACGCGTGC
TACACTGACGCATACAGCGA-------GTATCA----------CCAGCTCCGTGAGG-CTGCTGG-TAATCA--
GCAATATGCGTC-GTGCTGGGGATAGA-
TCTTTGGAATTTTTGATCTTGAACGAGGAATGCCTAGTAAGCGCAGGTCATCAGCCTGCGTTGATTAAG
TCCCTGCCCTTTGTACACACCGCCCGTCGCTCCTACCGATTTCGAGTGGTCCGGTGAACCC-
TTTGGACCGAGAGCGC---CTCGTGCGC---
TTTTGGAAAGTCGAGTAAACCACATCACTTAGAGGAAGGAAAA?????????????

Uronychia_setigera GCG-
ATATACAGTGAAACTGCGAATGGCTCATTAAAACAGTTATAGTTTATTTGATAATGGAATT-------------
TTATATGGATAACCGTAGTAATTCTAGAGCTAATACATGCTGTTTAGCCCGACTTTT-
CGGAAGGGCTGTATTTATTAGATA-ACAAACCAATATTCCT-TCG-
GGTCTATTGTGATGATTCATAGTAAC--TGTTCGAA-
TCGCATGGGCCTTGCCCGCGATAAATCATTCAAGTTTCTGC--CCCATCA-GCTT--
GATGATAATGTATTGGACTACCATGGCGCTCACGGG-
TAACGGAGAATTAGGGTTCGATTCCGGAGAGGGAGCCTGAGAAACGGCTACCACATCTAAGGAAGGC
AGCAGGCGCGTAAATTACCCAATCCTAATTCAGGGAGGTAGTGACAAGAAATAACAGGCCGGTGCTTC
---GCATCGTGTTTGCAAT-GAGTTTAATCCA-
AAACCCTTTTCTAGTAACAATTGGAGGGCAAGTCTGGTGCCAGCAGCCGCGGTAATTCCAGCTCCAATA
GCGTATATTAAAGTTGTTGCAGTTA--AAAAGCTCGTAGTTGGATTTCTGA-----------AAGCGCGA-
CAATG-TCCGC---TGATTGCGTG-TG-CAGCGTTGCTCTTTCATCCTTCTGTTAACG-
TTTCTTGGTATTCATTT----ACTGG--TTTCGGGCTCAGATATTTTACC---------------------------------------
TTGAGAAAATTAGAGTGTTTCAGGCAGGCTTGTGCT-
GGAATACATTAGCATGGAATAATGGAAAAGGACTT------------------TGGTCTCTTTTATTGGT-
TTAAGGGACCTCAG-TAATGATTAAT-AGGGATAGTT------------
GGGGGCATTAGTATTTAATTGTCAGAGGTGAAATTCTTAGATTTGTTAAAGACTAACTTATGCGAAAGC
A---TTTGCCAAGGATGTTTTCATTAATCA-
AGAACGAAAGTTAGGGGATCAAAGACGATCAGATACCGTCCTAGTCTTAACCATAAACTATGCCGACT
AGGGATCGGAGG-TGTGTGTTTT--TCCACCTTCGGCACC-TTATGAGAAATCAAAGTC-
TTTGGGTTCTGGGGGGAGTATGGTCGCAAGGCTGAAACTTAAAGGAATTGATGGAAGGGCACCACCA
GGAGTGGAGCTTGCGGCTTAATTTGACTCAACGCGGGGAAACTTACCAGGTCCAGACATAGGAAGGAT
TGACAGATTGATAGCTCTTTCTTGATTCTATGGG-----
TGGTGGTGCATGGCCGTTCTTAGTTGGTGGAGTGATTTGTCTGGTTAATTCCGTT-
AACGAACGAGACCTTAGCCTGCTAAATAGTCAC-TGTCCTTTC-AGGAC---
TTTCGACTTCTTAGAGGGACTTTGTG-ATTT-
AAACACAAGGAAGTTTGAGGCAATAACAGGTCTGTGATGCCCTTAGATGTCCTGGGCCGCACGCGTGC
TACACTGACGCATACAACGA-------GCACCT---------TCCTGCACCGCGAGG-TTGCAGG-TAATCT--
GCAATATGCGTC-GTGATGGGGATAGA-
TCTTTGGAATTATAGATCTTGAACGAGGAATTCCTAGTAAGCATAAGTCATCATCTTGTGCTGATTAAGT
CCCTGCCCTTTGTACACACCGCCCGTCG???
???

;

proc/;
