# Supplementary material for: A putatively extinct higher taxon of Spirotrichea (Ciliophora) from the Lower Cretaceous of Brazil
Source: Sci Rep. 2021 Sep 27;11:19110. doi: 10.1038/s41598-021-97709-2 (PMC8476538; doi:10.1038/s41598-021-97709-2)
Supplement: Supplementary file 3 — Supplementary Information 3. [file 41598_2021_97709_MOESM3_ESM.docx]

**Supplementary Materials**

**Character list**

Morphological characters were proposed with emphasis on the higher taxa relationships, with primary homologies based on our interpretation of the literature sources listed in Table S1, and considering the identification of *P. bahiensis* as a spirotrich ciliate to be correct. After aligned and trimmed, the 18S rDNA matrix had 1,858 characters (including gaps), of which 599 were parsimony informative. Character numbering follows TNT 1.5.^1^ default system. The combined matrix is available as Supplementary Data 2.

**0.** **Body, overall shape:**

0. Dorsoventrally or laterally flat;

1. Obovoid or obconical.

**1.** **Lorica, presence:**

0. Absent;

1. Present.

**2.** **Contractile vacuole, presence:**

0. Absent;

1. Present.

**3.** **Contractile vacuole, position:**

0. Posterior region of body;

1. Right side of body;

2. Left side of body.

**4.** **Frontal scutum, cephalization:**

0. Inconspicuous;

1. Conspicuous.

**5.** **Peristome, length relative to body length:**

Remarks: See character 0 in Paiva^2^. The measured values were rescaled to five integer states in the present study. The lower the value, the larger the relative peristome length.

0–4.

**6.** P**eristome, somatic ciliature close to left border, specialization level:**

0. Without evident specialization;

1. Moderately specialized, elements in tightly packed rows;

2. Highly specialized, forming cirri or brushes.

**7.** **Adoral zone, shape:**

0. Open;

1. Closed.

**8.** **Adoral zone, position:**

0. Ventrolateral;

1. Apical.

**9.** **Paroral membrane, presence:**

0. Absent;

1. Present.

**10.** **Paroral membrane, complexity:**

0. Thin or simple, usually formed by two rows of basal body at most of its extension.

1. Thick or complex, usually formed by many rows or polystichomonad.

**11.** **Endoral membrane, presence:**

0. Absent;

1. Present.

**12.** **Stomatogenesis, oral anlage development site:**

0. At or only slightly below cell surface.

1. Below cell surface, may occur in a pouch or tube.

**13.** **Frontal ciliature, spanning to the dorsal side:**

0. Absent;

1. Present.

**14.** **Fronto-ventral-transverse ciliature, differentiation from dorsal ciliature:**

0. Absent;

1. Present.

**15.** **Fronto-ventral-transverse anlagen, maturation, time:**

0. Before cytokinesis;

1. After cytokinesis or neotenic.

**16.** **Fronto-ventral-transverse anlagen, fronto-ventral product, topological differentiation:**

0. Ciliary elements with little or no topologic differentiation;

1. Ciliary elements topologically differentiated, forming specific frontal and fronto-ventral cirral groups.

**17.** **Transverse cirri or cirri-like structures, presence:**

0. Absent;

1. Present.

**18.** **Transverse cirri or cirri-like structures, inclination:**

Remarks: This character is calculated similarly to the "posterior fringe ratio" for odontostomes^3^.

0. Inclined to the left;

1. More or less perpendicular to body length or not leaning to either left or right;

2. Inclined to the right.

**19.** **Left marginal ciliature, presence:**

0. Absent;

1. Present.

**20.** **Left marginal ciliature, origin:**

Remarks: In Protohypotrichia, the left marginal cirral row is called migratory cirral row^4^ because it originates from a detaching patch of basal bodies migrating from the rightmost ventral anlage (herein interpreted as the right marginal anlage)^5^. In the other taxa, the left marginal ciliature originates from actual anlagen produced near or from the parental structures. A curious exception is *Diophryopsis* *hystrix* (not included here), where the sole left marginal cirrus is formed by dorsal kinety anlage 1^6^.

0. From actual left marginal anlagen;

1. Migratory from right marginal anlagen.

**21.** **Right marginal ciliature, presence:**

0. Absent;

1. Present.

**22.** **Caudal cirri, presence:**

0. Absent;

1. Present.

**23.** **Aboral disc, presence:**

0. Absent;

1. Present.

**24.** **Macronucleus, replication bands, presence:**

Remarks: Macronuclear replication bands do occur in *Phacodinium metchnikoffi*^7^, although highly modified^8^.

0. Absent;

1. Present.

**25.** **Cytokinesis, orientation:**

Remarks: The enantiotropy of divisional morphogenesis in tintinnids is less pronounced than in oligotrichids and not clear in many cases. In the present study, we code it as enantiotropic following the definition by Corliss^9^. The reader is referred to this matter in Petz & Foissner^10^.

0. Homotropic;

1. Enantiotropic;

2. Roughly symmetrogenic.

**Table S1**. Taxon sample used in the present study, literature sources and NCBI/GenBank retrieving codes for the 18S rDNA sequences. D = Discocephalida; E = Euplota; He = Heterotrichea (outgroup); Hy = Hypotricha; L = Licnophoria; O = Oligotricha; Pa = Palaeohypotricha nov. tax.; Ph = Phacodiniidia; Pr = Protohypotrichia. * = source reference for illustrative drawings in Figure 5 of the main text.

| **Terminal** | **Higher taxon** | **Morphology** | **18S rDNA** |
| --- | --- | --- | --- |
| *Anteholosticha marimonilata* | Hy | Xu et al.^11^ | FJ870075 |
| *Antetintinnidium mucicola* | O | Ganser & Agatha^12^ | KU715767 |
| *Aspidisca leptaspis* | E | Song & Wilbert^13^; Song^14^ | EU880597 |
| *Blepharisma americanum* | He | Aescht & Foissner^15^ | M97909 |
| *Blepharisma hyalinum* | He | Aescht & Foissner^15^ | AM713184 |
| *Caryotricha minuta* | Pr | Miao et al.^16^ Xu et al.^17^ | EU275202 |
| *Caryotricha rariseta* | Pr | Jiang et al.^18^ | FJ876978 |
| *Caryotricha sinica* | Pr | Lian et al. ^4^ | MN543654 |
| *Certesia quadrinucleata* | E | Wicklow^19^; Lin & Song^20^ | DQ059581 |
| *Diophrys appendiculata* | E | Hill^21^; Song & Packroff ^22^ | AY004773 |
| *Discocephalus ehrenbergi* | D | Wicklow^23^; Li et al^24^. * | FJ196397 |
| *Epiclintes auricularis rarisetus* | Hy | Hu et al.^25^ | FJ008722 |
| *Euplotes aediculatus* | E | Zhang et al.^26^* | LT628497 |
| *Euplotidium itoi* | E | Giambelluca et al.^27^ | HE775110 |
| *Gastrocirrhus monilifer* (ontogeny from *G. stentoreus*) | E | Hu & Song^28^ | DQ864734 |
| *Holosticha heterofoissneri* | Hy | Berger^29^; Song et al.^30^* | KP717082 |
| *Kiitricha marina* | Pr | Shao et al.^5^; Li et al.^31^ (2009); Song & Wilbert^32^ | AY896768 |
| *Licnophora lyngbycola* | L | Song et al.^33^* | DQ445606 |
| *Licnophora macfarlandi* | L | Balamuth^34^ | AF527758 |
| *Metaurostylopsis antarctica* | Hy | Jung et al.^35^ | JF906730 |
| *Oxytricha granulifera* | Hy | Foissner & Adam^36^ | AM412768 |
| *Palaeohypotrhix bahiensis* gen. *et* sp. nov. | Pa | Present study |  |
| *Phacodinium metchnikoffi* | Ph | Kahl^37^*; De Calvo^7^ | AJ277877; MN176351 |
| *Prodiscocephalus borrori* | D | Wicklow, 1982^23^; Shao et al.^38^ | DQ646880 |
| *Propecingulum fistoleramalliei* | O | Küppers et al.^39^* | MT124525 |
| *Pseudoamphisiella alveolata* | D | Shao et al.^40^ | DQ503583 |
| *Stenosemella pacifica* | O | Agatha & Tsai^41^ | JN831793 |
| *Uronychia setigera* | E | Song et al.^42^ | HQ380021 |


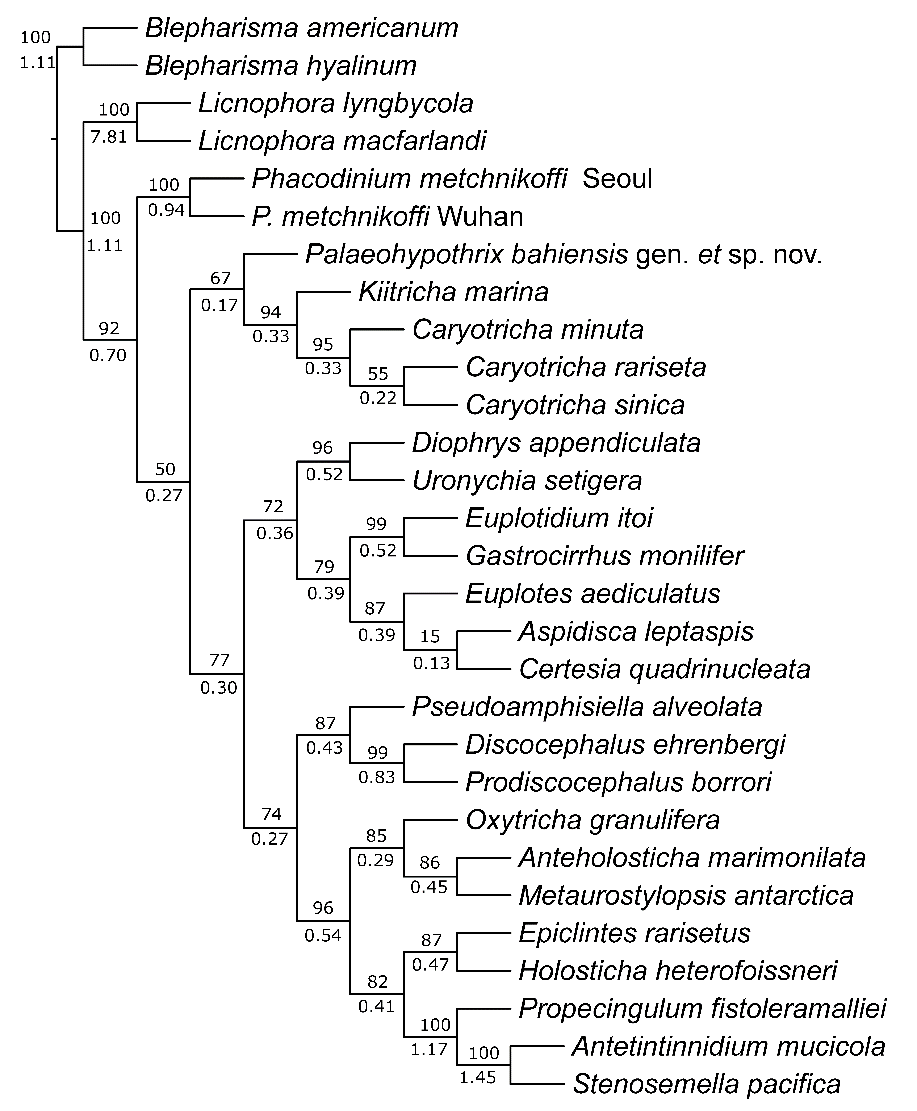


**Figure S1.** Optimal cladogram resulting from extended implied weights cladistic analysis (matrix in TNT format available as Supplementary Data 2). Score (*k* = 4.765625) 189.07802; CI = 0.44; RI = 0.51. The values above and below internal branches are GC frequencies and Bremer support, respectively.

**Data S1 (separate file).**

Unedited sequential photographs of *Palaeohypothrix bahiensis* gen. et sp. nov. (holotype) used to build volumetric reconstructions, given in a ZIP file.

**Data S2 (separate file).**

Matrix of morphological and molecular (18S rDNA) characters in TNT format.

**References**

1. Goloboff, P. A. & Catalano, S. A. TNT version 1.5, including a full implementation of phylogenetic morphometrics. *Cladistics* **32**, 221–238 (2016).

2. da Silva Paiva, T. Systematic redefinition of the Hypotricha (Alveolata, Ciliophora) Based on Combined Analyses of Morphological and Molecular Characters. *Protist* **171**, (2020).

3. da Silva Paiva, T., Küppers, G. C., Lahr, D. J. G., Schweikert, M. & da Silva-Neto, I. D. *Discomorphella pedroeneasi* sp. nov. (Ciliophora, Odontostomatida): An anaerobic ciliate hosting multiple cytoplasmic and macronuclear endocytobionts. *Eur. J. Protistol.* **58**, (2017).

4. Lian, C., Luo, X., Warren, A., Zhao, Y. & Jiang, J. Morphology and phylogeny of four marine or brackish water spirotrich ciliates (Protozoa, Ciliophora) from China, with descriptions of two new species. *Eur. J. Protistol.* **72**, 125663 (2020).

5. Shao, C., Song, W., Warren, A. & Al-Rasheid, K. A. Morphogenesis of *Kiitricha marina* Nozawa, 1941 (Ciliophora, Spirotrichea), a possible model for the ancestor of hypotrichs s. l. *Eur. J. Protistol.* **45**, 292–304 (2009).

6. Shao, C., Zhang, Q., Al-Rasheid, K. A., Warren, A. & Song, W. Ontogenesis and molecular phylogeny of the marine ciliate *Diophryopsis hystrix*: Implications for the Systematics of the Diophrys-like species (ciliophora, spirotrichea, euplotida). *J. Eukaryot. Microbiol.* **57**, 33–39 (2010).

7. Calvo, P. P. de. *“ Phacodinium Metchnikoffi ”: Análisis Comparativo de Datos Morfológicos*. (Universidad Complutense de Madrid, 2010).

8. Irwin, N. A. T. *et al.* The function and evolution of motile DNA replication systems in ciliates. *Curr. Biol.* **31**, 66-76.e6 (2021).

9. Corliss, J. O. *The ciliated protozoa. Characteization, classification, and guide to the literature. 2nd. ed.* (Pergamon Press, 1979).

10. Petz, W. & Foissner, W. Morphogenesis in some freshwater tintinnids (Ciliophora, Oligotrichida). *Eur. J. Protistol.* **29**, 106–120 (1993).

11. Xu, Y. *et al.* Taxonomy, ontogeny and molecular phylogeny of *Anteholosticha marimonilata* spec. nov. (Ciliophora, hypotrichida) from the yellow sea, China. *Int. J. Syst. Evol. Microbiol.* **61**, 2000–2014 (2011).

12. Ganser, M. H. & Agatha, S. Redescription of *Antetintinnidium mucicola* (Claparède and Lachmann, 1858) nov. gen., nov. comb. (Alveolata, Ciliophora, Tintinnina). *J. Eukaryot. Microbiol.* **66**, 802–820 (2019).

13. Song, W. & Wilbert, N. Morphological investigations on some free living ciliates (Protozoa, Ciliophora) from China Sea with description of a new hypotrichous genus, *Hemigastrostyla* nov. gen. *Arch. für Protistenkd.* **148**, 413–444 (1997).

14. Song, W. Reconsideration of the morphogenesis in the marine hypotrichous ciliate, *Aspidisca leptaspis* Fresenius, 1865 (Protozoa, Ciliophora). *Eur. J. Protistol.* **39**, 53–61 (2003).

15. Aescht, E. & Foissner, W. Divisional morphogenesis in *Blepharisma americanum*, *B. undulans*, and *B. hyalinum* (Ciliophora: Heterotrichida). *Acta Protozool.* **43**, 71–92 (1998).

16. Miao, M. *et al.* *Caryotricha minuta* (Xu et al., 2008) nov. comb., a unique marine ciliate (Protista, Ciliophora, Spirotrichea), with phylogenetic analysis of the ambiguous genus *Caryotricha* inferred from the small-subunit rRNA gene sequence. *Int. J. Syst. Evol. Microbiol.* **59**, 430–438 (2009).

17. Xu, K., Lei, Y. & Choi, J. K. *Kiitricha minuta* n. sp., a peculiar hypotrichous ciliate (Ciliophora, Spirotrichea) from the Yellow Sea. *J. Eukaryot. Microbiol.* **55**, 201–206 (2008).

18. Jiang, J. *et al.* Two new marine ciliates, *Caryotricha rariseta* n. sp. and *Discocephalus pararotatorius* n. sp. (Ciliophora, Spirotrichea), with phylogenetic analyses inferred from the small subunit rRNA gene sequences. *J. Eukaryot. Microbiol.* **60**, 388–398 (2013).

19. Wicklow, B. J. Ultrastructure and cortical morphogenesis in the euplotine hypotrich *Certesia quadrinucleata* Fabre-Domergue, 1885 (Ciliophora, Protozoa) 1. *J. Protozool.* **30**, 256–266 (1983).

20. Lin, X. & Song, W. Redescription of the marine ciliate, *Certesia quadrinucleata* (Protozoa: Ciliophora) from Qingdao, China. *J. Mar. Biol. Assoc. United Kingdom* **84**, 1131–1136 (2004).

21. Hill, B. F. The cortical morphogenetic cycle associated with cell division in diophrys Dujardin, 1841 (Ciliophora, Hypotrichida). *J. Protozool.* **28**, 215–221 (1981).

22. Song, W., Packroff, G. Beitrag zur Morphogenese des marinen Ciliaten *Diophrys scutum* (Dujardin, 1841) (Ciliophora, Hypotrichida). *Zool. Jb. Anat.* **123**, 85–95 (1997).

23. Wicklow, B. J. The Discocephalina (n. subord): Ultrastructure, morphogenesis and evolutionary implications of a group of endemic marine interstitial hypotrichs (Ciliophora, Protozoa). *Protistologica* **18**, 299–330.

24. Li, L. *et al.* Two discocephalid ciliates, *Paradiscocephalus elongatus* nov. gen., nov. spec. and *Discocephalus ehrenbergi* Dragesco, 1960, from the Yellow Sea, China (Ciliophora, Hypotrichida, Discocephalidae). *Acta Protozool.* **42**, 353–362 (2009).

25. Hu, X., Fan, X., Lin, X., Gong, J. & Song, W. The morphology and morphogenesis of a marine ciliate, *Epiclintes auricularis rarisetus* nov. sspec. (Ciliophora, Epiclintidae), from the Yellow Sea. *Eur. J. Protistol.* **45**, 281–291 (2009).

26. Zhang, X. *et al.* Morphology, ontogeny and molecular phylogeny of *Euplotes aediculatus* Pierson, 1943 (Ciliophora, Euplotida). *Biodivers. Sci.* **25**, 549–560 (2017).

27. Giambelluca, M. A., Gabrielli, S., Erra, F. & Rosati, G. Morphogenetic study during cell division in *Euplotidium itoi* (ciliata, hypotrichida). *Eur. J. Protistol.* **31**, 286–291 (1995).

28. Hu, X. & Song, W. Redescription of Two Known Species, *Gastrocirrhus monilifer* (Ozaki et Yagiu, 1942) and *Gastrocirrhus stentoreus* Bullington, 1940, with Reconsideration of the Genera *Gastrocirrhu*s and *Euplotidium*. *Acta Protozool.* **42**, 345–355 (2003).

29. Berger, H. Monograph of the Urostyloidea (Ciliophora, Hypotricha). *Monogr. Biol.* **85**, 1–1304 (2006).

30. Song W., Wilbert N., Warren A. (2002): New contribution to the morphology and taxonomy of four marine hypotrichous ciliates from Qingdao, China (Protozoa: Ciliophora). *Acta Protozool*. **41**, 145–162 (2002)

.31. Li, L. *et al.* Does *Kiitricha* (Protista, Ciliophora, Spirotrichea) belong to Euplotida or represent a primordial spirotrichous taxon? With suggestion to establish a new subclass Protohypotrichia. *Int. J. Syst. Evol. Microbiol.* **59**, 439–446 (2009).

32. Shao, C., Song, W., Al-Rasheid, K. A. S. & Berger, H. Redefinition and reassignment of the 18-cirri genera *Hemigastrostyla, Oxytricha, Urosomoida,* and *Actinotricha* (ciliophora, hypotricha), and description of one new genus and two new species. *Acta Protozool.* **50**, 263–287 (2011).

33. Song, W., Warren, A., Daode, J. I., Wang, M. & Al-Rasheid, K. A. S. New contributions to two heterotrichous ciliates, *Folliculina simplex* (Dons, 1917), *Condylostoma curva* Burkovsky, 1970 and One licnophorid, *Licnophora lyngbycola* Fauré-Fremiet, 1937 (Protozoa, Ciliophora): Descriptions of morphology and infraciliature. *J. Eukaryot. Microbiol.* **50**, 449–462 (2003).

34. Balamuth, W. Studies on the organization of ciliate Protozoa I. Microscopic anatomy of *Licnophora macfarlandi*. *J. Morphol.* **68**, 241–277 (1941).

35. Jung, J. H., Baek, Y. S., Kim, S., Choi, H. G. & Min, G. S. A new marine ciliate, *Metaurostylopsis antarctica* nov. spec. (Ciliophora, Urostylida) from the Antarctic Ocean. *Acta Protozool.* **50**, 289–300 (2011).

36. Foissner, W., Adam, H. Morphologie und Morphogenese des Bodenciliaten *Oxytricha granulifera* sp.n. (CiIiophora, Oxytrichidae). *Zool. Scr.* **12**, 1–11 (1983).

37. Kahl, A. Urtiere oder Protozoa I: Wimpertiere oder Ciliata (Infusoria) 3. Spirotricha. *Tierwelt Dtl.* **25**, 399–650 (1932).

38. Shao, C. *et al.* Systematic position of Discocephalus-like ciliates (Ciliophora: Spirotrichea) inferred from SSU rDNA and ontogenetic information. *Int. J. Syst. Evol. Microbiol.* **58**, 2962–2972 (2008).

39. Küppers, G. C., da Silva Paiva, T., do Nascimento Borges, B., Alfaro, E. R. & Claps, M. C. A new oligotrich (Ciliophora, Oligotrichia) from Argentina, with redefinition of *Novistrombidium* Song and Bradbury. *Eur. J. Protistol.* **69**, 20–36 (2019).

40. Shao, C. *et al.* Morphogenesis of the marine ciliate, *Pseudoamphisiella alveolata* (Kahl, 1932) Song & Warren, 2000 (Ciliophora, Stichotrichia, Urostylida) during binary fission. *J. Eukaryot. Microbiol.* **53**, 388–396 (2006).

41. Agatha, S. & Tsai, S.-F. Redescription of the tintinnid *Stenosemella pacifica* Kofoid and Campbell, 1929 (Ciliophora, Spirotricha) based on live observation, protargol impregnation, and scanning electron microscopy. *J. Eukaryot. Microbiol.* **55**, 75–85 (2008).

42. Weibo Song, Norbert Wilbert, Zigui Chen, X. S. Considerations on the systematic position of *Uronychia* and related euplotids based on the data of ontogeny and 18S rRNA gene sequence analyses, with morphogenetic redescription of *Uronychia setigera* Calkins, 1902 (Ciliophora: Euplotida). *Acta Protozool.* **43**, 313–328 (2004).
